# Programmable Photocatalytic Activity of Multicomponent Covalent Organic Frameworks Used as Metallaphotocatalysts

**DOI:** 10.1002/chem.202202967

**Published:** 2022-12-01

**Authors:** Michael Traxler, Susanne Reischauer, Sarah Vogl, Jérôme Roeser, Jabor Rabeah, Christopher Penschke, Peter Saalfrank, Bartholomäus Pieber, Arne Thomas

**Affiliations:** ^1^ Department of Chemistry/Functional Materials Technische Universität Berlin Hardenbergstraße 40 10623 Berlin Germany; ^2^ Department of Biomolecular Systems Max Planck Institute of Colloids and Interfaces Am Mühlenberg 1 14476 Potsdam Germany; ^3^ Department of Chemistry and Biochemistry Freie Universität Berlin Takustraße 3 14195 Berlin Germany; ^4^ Leibniz Institute for Catalysis (LIKAT Rostock) Universität Rostock Albert-Einstein-Straße 29a 18059 Rostock Germany; ^5^ Institut für Chemie Universität Potsdam Karl-Liebknecht Straße 24–25 14476 Potsdam Germany

**Keywords:** **Keywords**: catalysis, covalent organic frameworks, cross-coupling, photoredox, tuneable activity

## Abstract

The multicomponent approach allows to incorporate several functionalities into a single covalent organic framework (COF) and consequently allows the construction of bifunctional materials for cooperative catalysis. The well‐defined structure of such multicomponent COFs is furthermore ideally suited for structure‐activity relationship studies. We report a series of multicomponent COFs that contain acridine‐ and 2,2’‐bipyridine linkers connected through 1,3,5‐benzenetrialdehyde derivatives. The acridine motif is responsible for broad light absorption, while the bipyridine unit enables complexation of nickel catalysts. These features enable the usage of the framework materials as catalysts for light‐mediated carbon−heteroatom cross‐couplings. Variation of the node units shows that the catalytic activity correlates to the keto‐enamine tautomer isomerism. This allows switching between high charge‐carrier mobility and persistent, localized charge‐separated species depending on the nodes, a tool to tailor the materials for specific reactions. Moreover, nickel‐loaded COFs are recyclable and catalyze cross‐couplings even using red light irradiation.

## Graphical abstract

**Various multicomponent COFs** bearing both a moiety for photosensitization and complexation of nickel catalysts were synthesized using reticulation with different 1,3,5‐triformylbenzene nodes. This enabled the switching between persistent, charge‐separated species or efficient charge‐carrier mobility for different metallaphotocatalytic cross‐coupling reactions based on the node unit. The frameworks showed recyclability and the possibility to drive the reactions using red light irradiation.

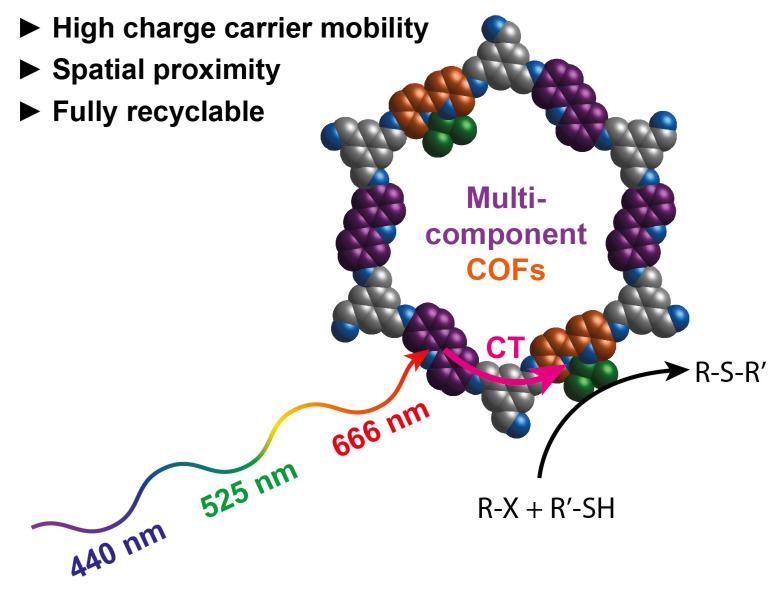

## Introduction

Combining two or more catalysts that work in concert to enable the formation of a chemical bond (cooperative catalysis, dual catalysis) has recently become a powerful addition to the synthetic chemist's toolbox.^[1]^ In particular, the combination of nickel‐ and photocatalysis (metallaphotocatalysis) has led to the discovery of a number of carbon−heteroatom (C−X) and carbon−carbon (C−C) cross‐coupling methods that are carried out under mild conditions using visible‐light.[^2^, ^3^, ^4^, ^5^] These reactions are typically carried out using a photocatalyst in combination with a molecular, homogeneous nickel catalyst. The efficacy of such complex systems depends on a multitude of parameters that are individually optimized to maximize product formation. These include irradiation wavelength, photon flux, activity/selectivity of the nickel catalyst, photoelectronic properties of photocatalysts, base, temperature, solvent and stoichiometry, among others.

The interaction between the catalysts in solution requires persistent excited state lifetimes of the photocatalyst as it must be longer than the time it takes to diffuse to the nickel complex to induce catalysis. Thus, these transformations are limited to photocatalysts that populate triplet excited states with high quantum yields, such as Ir or Ru polypyridyl complexes,^[6]^ and organic compounds that show thermally activated delayed fluorescence.^[7]^ Similarly, certain semiconducting materials generate sufficiently long‐lived charge‐separated species.^[8]^

Close spatial proximity between the two catalysts is arguably beneficial for electron (or energy) transfer events between the two catalytic species, but this parameter is difficult to tune using homogeneous catalysts. Immobilizing the photo‐ and the nickel catalyst on a support enables controlling the distance between the individual catalysts at the nanoscale. For example, iridium polypyridyl‐ and nickel bipyridine complexes were integrated in metal‐organic frameworks (MOFs),[^9^, ^10^] covalent organic frameworks (COFs),[^11^, ^12^] and linear polymers.^[13]^ Indeed, these bifunctional catalysts often showed synergistic effects in terms of higher turnover numbers and catalytic activities in C−C and C−X cross‐couplings as compared to using the individual catalysts in solution. A similar trend was observed for MOFs that have incorporated catalysts for dual photo‐/Lewis acid catalysis.[^14^, ^15^] Decoupling the interaction between the photocatalyst and the nickel catalyst from the limiting rate of diffusion was also shown to increase the arsenal of suitable photocatalysts for metallaphotocatalytic reactions.[^16^, ^17^] However, these concepts rely on the immobilization of photo‐ and nickel catalysts on a supporting material that potentially influences transmission of the generated charge carriers to the active metal site.

Another appealing strategy is to integrate a nickel catalyst directly into a photocatalytically active polymeric material. Seminal approaches include the incorporation of nickel atoms into carbon nitride materials,[^18^, ^19^, ^20^, ^21^] and a conjugated microporous polymer that contains a bipyridine motif in its repeating unit that can ligate nickel atoms.[^22^, ^23^] However, these ill‐defined macrostructures do not allow for detailed structure‐activity relationship studies to better understand the underlying processes, which renders knowledge‐guided improvement of the bifunctional materials difficult.

Covalent organic frameworks (COFs) are well‐defined, crystalline, highly porous polymers with tunable structures that are prepared by covalently attaching multiconnected nodes with linear linkers, which can introduce functional groups into the backbone.[^24^, ^25^, ^26^, ^27^, ^28^] When a three or higher connected node component is allowed to react with more than one linker unit, several functionalities can be integrated into the backbone of a single COF material (multicomponent approach).[^29^, ^30^, ^31^, ^32^, ^33^, ^34^] Owing to their conjugated backbones, COF materials have been shown to serve as valuable photocatalysts.[^35^, ^36^, ^37^, ^38^, ^39^] Using the modularity of COFs the optoelectrical properties can be influenced through, for example, the choice of the node component.[^40^, ^41^] We have shown that COFs bearing acridine linkers are promising metal‐free, heterogeneous photocatalyst that can be combined with homogeneous nickel complexes for metallaphotocatalysis.^[39]^ Our results suggested that the *β*‐ketoenamine to imine ratio on the node that connects the linkers is a key parameter influencing charge‐carrier separation. Others have reported the integration of nickel complexes into a photocatalytically active COF structure to realize visible‐light mediated cross‐couplings[^42^, ^43^] or hydroxylation of aryl chlorides with water.^[44]^

Inspired by these studies, we hypothesized that a multicomponent COF prepared from an acridine linker, a linking unit that provides coordination sites for nickel, and benzene‐1,3,5‐tricarbaldehyde derivatives as 3‐connected (3‐c) nodes would enable detailed structure‐activity relationship studies that shine light on parameters that influence the activity of such bifunctional multicomponent COF metallaphotocatalysts. Here, we show that the *β*‐ketoenamine to imine ratio significantly varies the efficacy of such COFs as catalysts for C‐S and C‐N cross‐couplings. Our results show that the imine‐form leads to high charge‐carrier mobility that is ideal for activating nickel sites that are ligated at the COF backbone. In contrast, *β*‐ketoenamine tautomerized COFs have localized, persistent charge‐separated species that are key for diffusion limited interaction with homogeneous nickel complexes.

## Results and Discussion

**Figure 1:**
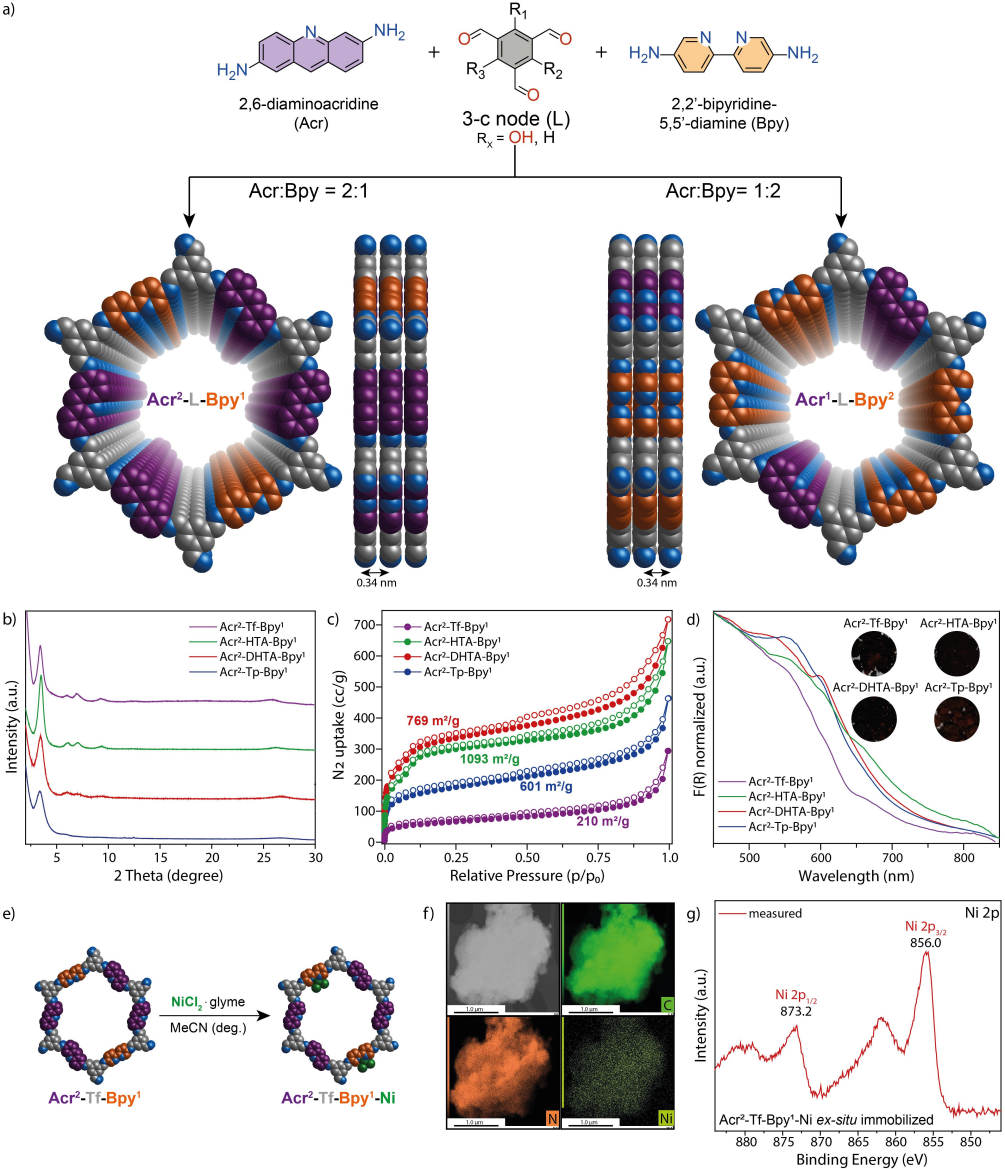
Synthesis and characterization of Acr^x^‐L‐Bpy^y^ (x : y=1 : 2 or 2 : 1; L=Tp, DHTA, HTA or Tf). a) Scheme of the synthesis of the COFs; top and side views of Acr^x^‐L‐Bpy^y^ COFs showing the ideal eclipsed (AA) structures. b) Experimental X‐ray diffraction patterns for Acr^2^‐L‐Bpy^1^. c) N_2_ sorption isotherms for Acr^2^‐L‐Bpy^1^ COFs, calculated BET surface areas are shown in the inlets. d) UV‐vis diffuse reflectance spectra for Acr^2^‐L‐Bpy^1^ COFs. The inset shows optical images of the COF powders. e) Schematic illustration of the preparation of Acr^2^‐Tf‐Bpy^1^‐Ni. f) TEM image of Acr^2^‐Tf‐Bpy^1^ and the elemental mapping of carbon, nitrogen and nickel. g) XPS Ni 2p core‐level spectrum of Acr^2^‐Tf‐Bpy^1^‐Ni.

**Figure 2:**
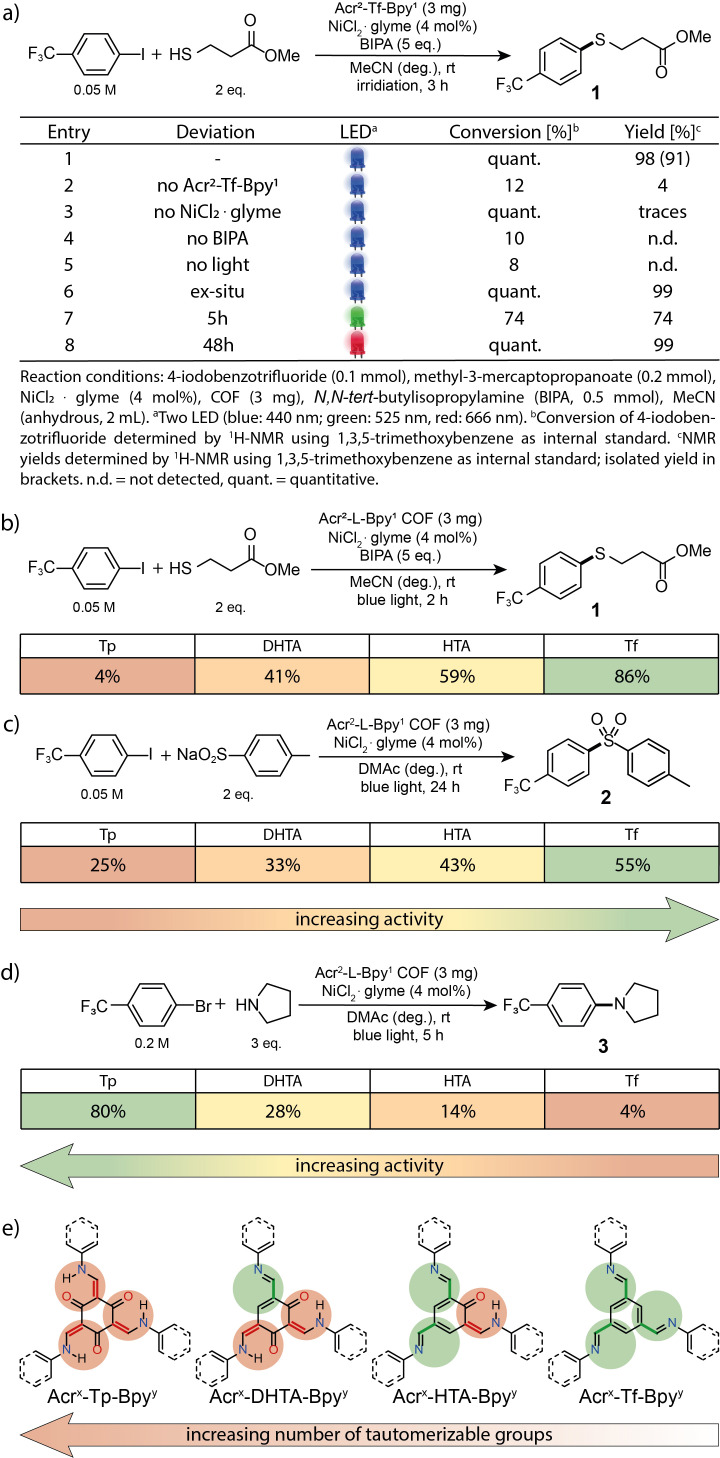
a) Optimized conditions and control experiments using blue, green and red light for the C−S between 4‐iodobenzotrifluoride and 3‐mercaptopropionate. Screening of Acr^2^‐L‐Bpy^1^ COFs for the coupling of 4‐iodobenzotrifluoride with b) 3‐mercaptopropionate, c) sodium *p*‐toluensulfinate and d) 4‐bromobenzotrifluoride with pyrrolidine (NMR yields determined by ^1^H NMR using internal standard). e) COFs bearing linkers with different tautomerizable groups.

Our investigations started with the synthesis of a set of eight multicomponent COFs using 2,6‐acridinediamine (Acr) as chromophoric linker and 2,2’‐bipyridine‐5,5’‐diamine (Bpy) as metal binding site (Figure 1a). Since both linkers are varying in length, an Acr/Bpy ratio of 2 : 1 and 1 : 2 was used to ensure the formation of extended structures of the expected honeycomb topology.^[30]^ The number of hydroxy groups on the 3‐connected benzene‐1,3,5‐tricarbaldehyde nodes was varied within each series of Acr/Bpy COFs to investigate the influence of the *β*‐ketoenamine to imine ratio on charge‐carrier mobility. 1,3,5‐triformylphloroglucinol (Tp) was used to synthesize the irreversible keto tautomeric COFs, whereas 1,3,5‐triformylbenzene (Tf) carries no tautomerizable group and thus yields a fully imine linked COF (Figure 2e). 2,4‐dihydroxybenzene‐1,3,5‐tricarbaldehyde (DHTA) and 2‐hydroxybenzene‐1,3,5‐tricarbaldehyde (HTA) were used to prepare COF backbones that have reversible tautomeric forms.^[40]^ All multicomponent Acr^x^‐L‐Bpy^y^ COFs (where x : y=2 : 1 or 1 : 2 and L=Tp, DHTA, HTA or Tf) were synthesized by an acid catalyzed Schiff base reaction (detailed information for the synthesis of all COFs can be found in Section S3.2 in the Supporting Information).

The crystallinity of the synthesized COFs was determined by powder X‐ray diffraction (PXRD) analyses using Cu K_α_ radiation (Figures 1b and S5). All materials show an intense reflection in the low angle area at around 2*θ*=3.5°, which can be assigned to the (100) facet of a primitive hexagonal lattice. Weaker reflections in the 2*θ*=5–10° range and a broad peak at 2*θ*=26.5° degrees confirmed the formation of crystalline, *π*‐*π* stacked two‐dimensional structures for all COFs. According to the symmetry of the linkers, the structural models for the multicomponent COFs were constructed by generating the expected 2D layers with **hcb** topology. The models were geometrically optimized, and their corresponding theoretical PXRD patterns were compared to the experimentally measured patterns. Theoretical diffraction patterns of models with eclipsed stacking sequences (AA) are in good agreement with the measured diffractograms (Figures S4 and S5).

Nitrogen sorption measurements at 77 K confirmed the porosity of all COFs (Figures 1c and S6). Based on the Brunauer–Emmett–Teller (BET) method, surface areas of the COFs were determined. Among the series of Acr^2^‐L‐Bpy^1^ COFs, Acr^2^‐HTA‐Bpy^1^ showed the highest specific surface area (1093 m^2^/g). The fully imine‐based COF Acr^2^‐Tf‐Bpy^1^ showed the lowest surface area of 210 m^2^/g. A different trend was found for Acr^1^‐L‐Bpy^2^ COFs, where the highest surface area was obtained using DHTA (926 m^2^/g) and the lowest using HTA (175 m^2^/g). The pore size of all Acr^2^‐L‐Bpy^1^ COFs are close to theoretical values (Figure S7).

Fourier transform infrared (FTIR) spectroscopy confirmed the formation of the framework materials. Characteristic signals of the precursors disappeared, while peaks that can be assigned to C=O and C=C or C=N vibrations, respectively, are present after the COF synthesis (Figures S8 and S9). Thermal stability of the multicomponent COFs was investigated using thermogravimetric analysis (TGA). After an initial weight loss due to adsorbed moisture and solvent molecules, all materials were thermally stable up to 350 °C (Figure S10).

The structural integrity of frameworks was studied by ^13^C cross‐polarization magic angle spinning nuclear magnetic resonance (CP‐MAS NMR) analyses (Figure S11). For Acr^x^‐Tp‐Bpy^y^ COFs, a distinct carbonyl carbon (C=O) peak around 180–185 ppm as well the C=C signal at 105 ppm confirm the dominant presence of the keto form.^[45]^ These two peaks decreased with a lower degree of tautomerization when less hydroxy groups were on the node and completely vanished for Acr^x^‐Tf‐Bpy^y^, indicative of increasing amounts of imine tautomers among the series. Signals between 115–150 ppm confirm the presence heteroaromatic linkers in all COF backbones. Diagnostic signals at 150 and 133 ppm were assigned to the bipyridine motif and confirm an increasing bipyridine:acridine ratio when higher amounts of 2,2’‐bipyridine‐5,5’‐diamine were used for the COF synthesis (Figure S25b).[^46^, ^47^]

X‐ray photoelectron spectroscopy (XPS) also depicted the gradual reduction of keto‐enamine tautomerization in the series of COFs. Deconvolution revealed signals in the N 1s spectrum at 400 eV and 398.8 eV that can be assigned to the secondary amine group of the keto‐enamine linkage and imine and pyrdinic groups, respectively. The ratio between these signals gradually decreases with decreased number of tautomerizable groups and vanishes for fully imine‐based COFs Acr^x^‐Tf‐Bpy^y^ (Figure S12). Aromatic nitrogen atoms of the acridine and bipyridine atoms show a signal with the binding energy of 399 eV in the N 1s spectrum. The different amounts of bipyridine units within the COF backbones can be determined qualitatively, with a more pronounced signal for Acr^1^‐Tp‐Bpy^2^ compared to Acr^2^‐Tp‐Bpy^1^. For the other COFs this peak is overlying with the signals of the imine and enol bonded nitrogen atoms. To quantitatively determine the amount of Acr and Bpy units in the backbone of the multicomponent materials, Acr^x^‐Tp‐Bpy^y^ COFs were digested in a solution of 0.1 mL of 10 M NaOH in D_2_O and 0.5 mL DMSO‐d_6_ at 120 °C and subsequently analyzed with ^1^H NMR. Integration of the peaks at 7.89 and 7.69 ppm that can be assigned to Bpy and Acr, respectively, show that the amount of substrates used in the synthesis is in agreement with the incorporated ratio of the two linkers, demonstrating no preferential reaction of the linkers (Figure S13).

Using diffuse reflectance ultraviolet‐visible (UV‐vis) spectroscopy we confirmed that multicomponent COFs broadly absorb across the visible‐light spectrum (Figures 1d and S15a). The absorption onsets of all COFs are above 700 nm. In comparison, COFs that exclusively contain bipyridine linkers (L‐Bpy) absorb less broadly (Figure S24). This shows that the introduction of the acridine moiety is key for efficient solar harvesting.^[39]^ Using calculations on the level of density functional theory (DFT) we discovered that the introduction of a bipyridine linker into the backbone of the framework is not changing the band gap of the multicomponent Acr^2^‐L‐Bpy^1^ COFs (Section S4.10), confirming our experimental findings.

Before testing the materials in cross‐coupling reaction as suitable catalysts, nickel complexation at Acr^2^‐Tf‐Bpy^1^ was tested ex situ by refluxing a suspension of the COF and NiCl_2_⋅glyme in acetonitrile (Figure 1e). After confirming the crystallinity of the material by PXRD (Figure S31), the resulting Acr^2^‐Tf‐Bpy^1^‐Ni was characterized by transmission electron microscopy (TEM) and shows rather undefined morphology of the particles (Figure 1f). Elemental mapping illustrates a homogeneous distribution of nickel, nitrogen and carbon within the material. XPS analysis confirmed successful coordination of Ni^II^ on the bipyridine nitrogen atoms within the COF (Figure 1g). The Ni 2p spectrum showed the presence of nickel with a doublet at 855.7 eV and 873.4 eV, assigned to 2p_3/2_ and 2p_1/2_ signals for Ni^II^ species, respectively. Inductive coupled plasma – optical emission spectroscopy (ICP‐OES) was used to quantify the immobilization of the transition metal (Table S9) and showed a nickel loading of 3.59 mg g^−1^ corresponding to an occupation of 5.1 % of bipyridine functionalities.

With the fully characterized Acr^x^‐L‐Bpy^y^ COFs in hand, we sought to study if these bifunctional materials are suitable as heterogeneous metallaphotocatalysts for carbon‐heteroatom cross‐couplings in presence of a nickel(II) salt (in situ complexation). Indeed, Acr^2^‐Tf‐Bpy^1^ showed high catalytic activity in the C−S coupling of 4‐iodobenzotrifluoride with methyl 3‐mercaptopropanoate. Almost quantitative formation of the desired product (**1**) was achieved within three hours using 440 nm LEDs (Figure 2a, Entry 1). Control studies showed that only small amounts of the coupling product were formed without the Ni^II^ salt, or Acr^2^‐Tf‐Bpy^1^ (Entry 2,3). No reaction occurred in the absence of a base or light (Entry 4,5). Nickel complexation can also be carried out prior to catalysis (Entry 6).

High energy of blue light potentially causes deactivation of nickel catalysts and can lead to undesired side reactions.^[16]^ Longer wavelengths do not only serve as a tool to overcome such drawbacks, but also potentially provide better scalable protocols^[48]^ and enable irradiation through tissue,^[49]^ which is a promising feature towards biological applications. Indeed, Acr^2^‐Tf‐Bpy^1^ shows high catalytic activity using green light (Entry 7) and even results in quantitative product formation after 48 h using red LEDs (Entry 8).

By comparing the series of Acr^2^‐L‐Bpy^1^ COFs as catalysts, for the same cross‐coupling reaction a clear trend regarding the nodes was identified (Figure 2b). Fully tautomerized Acr^2^‐Tp‐Bpy^1^ showed the lowest catalytic activity with a yield of 4 % after 2 h irradiation time. Decreasing the number of hydroxy groups on the node resulted in a gradual increase of the desired product under identical conditions. The same activity pattern was observed in the coupling of 4‐iodobenzotrifluoride and sodium *p*‐toluenesulfinate (Figure 2c), where the same overall trend was found for Acr^1^‐L‐Bpy^2^ and L‐Bpy COFs, showing less than 45 and 36 % activity per added nickel center (Table S4). However, the related C‐N coupling between pyrrolidine and 4‐bromobenzotrifluoride showed a reversed trend (Figure 2d). Here, the keto‐tautomer Acr^2^‐Tp‐Bpy^1^ gave the highest yield, while the corresponding imine‐based COF showed low catalytic activity.

This discrepancy can be rationalized by the different involved mechanisms. C−S couplings require coordination of Ni to a bipyridine ligand. In C−N cross‐couplings, the secondary amine is added in large excess, because it simultaneously serves as substrate, base and, more importantly, ligand for the first‐row transition metal.^[50]^ As such, this reaction is catalyzed through a homogeneous Ni(pyrrolidine)_n_ complex that is activated by the COF that only acts as photocatalyst. Hence, the trend in the COF activity results from the fact that the irreversible tautomer stabilizes the conduction band electrons located at the acridine motif, resulting in a persistent charge‐separated species that is ideally suited for a diffusion limited dual catalytic interaction with a homogeneous nickel intermediate.^[39]^ In contrast, in the C−S cross‐couplings nickel catalysis requires bipyridine ligation and occurs therefore directly at the COF backbone. Consequently, this transformation benefits from high charge‐carrier mobility governed by a fully imine‐based backbone. These results are supported by electron paramagnetic resonance (EPR) spin trapping experiments that provide evidence for enhanced formation of spin adducts when a mixture of metalated Acr^2^‐Tf‐Bpy^1^‐Ni, 5,5‐dimethyl‐1‐pyrrolin‐*N*‐oxid (DMPO) and 4‐iodobenzotrifluoride in acetonitrile is irradiated compared to the Acr^2^‐Tp‐Bpy^1^‐Ni (Figure S19, see Section S4.12 for details).

**Figure 3:**
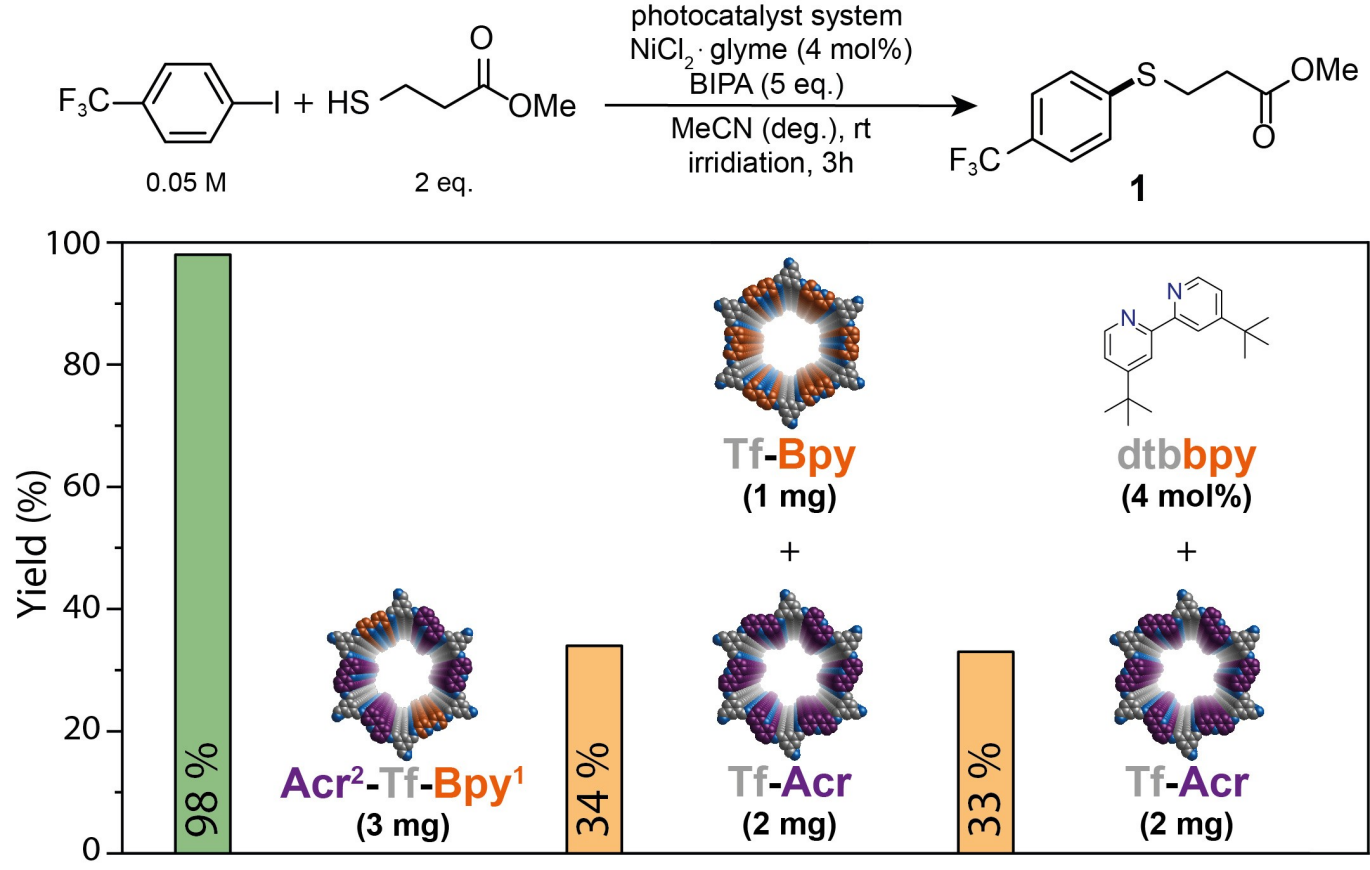
Comparison of the activities between multicomponent COF (left), physical mixture of respective COFs (middle) and semi‐heterogeneous catalysis (right).

Next, we aimed to investigate if the high spatiotemporal control that results from the close proximity of the photocatalyst (COF) and the nickel center in combination with the high charge‐carrier mobility is superior to related, diffusion limited transformations. We compared the catalytic activity of in situ nickel loaded Acr^2^‐Tf‐Bpy^1^ with two other catalytic systems in the cross‐coupling of 4‐iodobenzotrifluoride with methyl 3‐mercaptopropanoate (Figure 3). One catalytic system contained a mixture of a photocatalytic COF that only contains acridine units (Tf‐Acr; Section S5.3) with a nickel containing bipyridine COF (Tf‐Bpy, Section S5.1) in the ratio of 2 : 1 (Figure 3). The third catalytic cocktail combined the photocatalytic Tf‐Acr COF with a homogeneous nickel 4,4′‐di‐tert‐butyl‐2,2′‐dipyridyl (dtbbpy) complex. Under identical conditions (3 h at 440 nm), the bifunctional material produced the desired coupling molecule in almost quantitative yield, whereas both diffusion limited catalytic processes only resulted in modest conversion.

**Figure 4:**
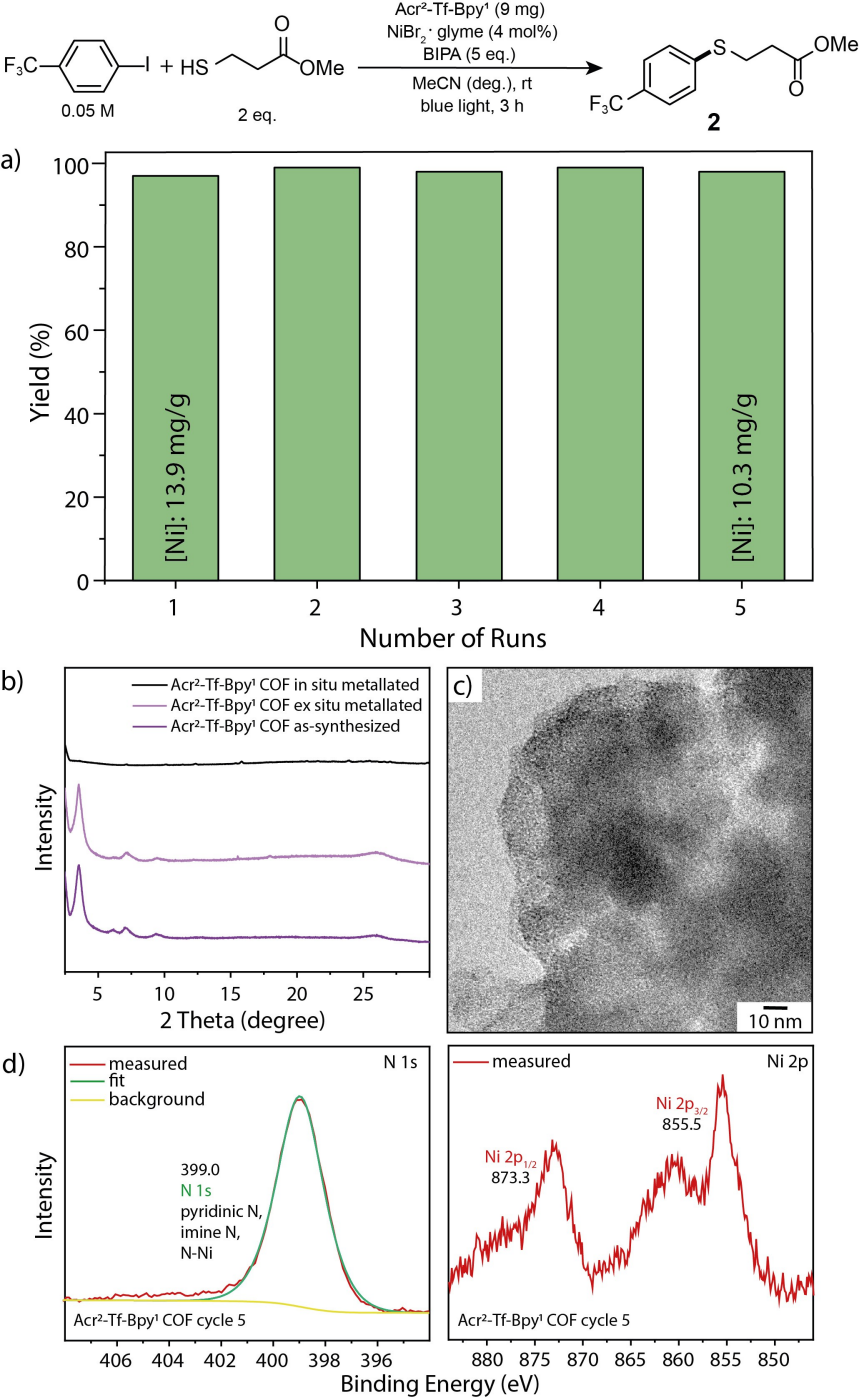
a) Recyclability studies of Acr^2^‐Tf‐Bpy^1^ for the C−S cross‐coupling between 4‐iodobenzotrifluoride and 3‐mercaptopropionate (NMR yields determined by ^1^H NMR using 1,3,5‐trimethoxybenzene as internal standard). b) PXRD analysis of Acr^2^‐Tf‐Bpy^1^ before and after photocatalysis (in situ metalation), and ex situ metalation. c) TEM analyses of Acr^2^‐Tf‐Bpy^1^ after photocatalysis. d) N 1s and Ni 2p XPS core level spectra of the Acr^2^‐Tf‐Bpy^1^ COF after 5 recycling cycles of photocatalytic dual nickel C−S cross‐coupling.

Finally, we studied the recyclability of the bifunctional material using blue (440 nm) light in the thioetherification (Figure 4a). The first experiment was carried through in situ catalyst formation using the conditions reported in Figure 2a. ICP‐analysis revealed that this material has a nickel content of 13.9 mg/g, corresponding to an occupation of 20.0 % of bipyridine functionalities. The difference in nickel loading compared to the ex situ prepared material described above can be attributed to the disruption of the *π‐π* stacking interactions by treating the material with light.[^37^, ^51^] This leads to loss of crystallinity using in situ metalation, enabling a larger amount of nickel to be immobilized (Figure 4b), due to the diminished long‐range order. However, after the respective reaction time, the heterogeneous catalyst was separated, washed and reused without adding additional Acr^2^‐Tf‐Bpy^1^‐Ni or nickel(II) salt. The bifunctional, nickel charged COF could be recycled five times without significant loss in catalytic activity, suggesting that the nickel atoms strongly coordinate to the bipyridine linkers in the COF. After the last cycle, a nickel loading of 10.3 mg/g showed that most of the nickel atoms present after the first cycle are still immobilized. FTIR spectroscopy confirmed the intact chemical structure of the COF after the reaction (Figure S32), which proves that the short‐range order of the material is preserved, although the long‐range periodicity is lost under photocatalytic conditions. Additionally, the morphology of the material was analyzed by TEM and did not alter during the recycling study (Figures 4c and S33). XPS spectra after 5 reaction cycles showed that both the N 1s and Ni 2p signal remained unchanged after the photocatalytic cross‐coupling reactions (Figure 4d). This confirms the formation of a fully heterogeneous nickel complex embedded into a stable COF matrix. Moreover, UV‐vis spectroscopy showed no change in optical properties after the reaction (Figure S35).

## Conclusion

In summary a series of multicomponent COFs containing a bipyridine and acridine linker were prepared using different three‐connected nodes and their application as catalysts for light‐mediated nickel catalyzed carbon−heteroatom cross‐couplings was evaluated. Four different 1,3,5‐triformylbenzene derivatives were selected as nodes to study the influence of *β*‐ketoenamine to imine ratio in the frameworks on catalytic activity. Our results show that the imine‐form is key for high charge‐carrier mobility that transfers conduction band electrons located at the acridine photocatalyst to an active nickel center that is attached at the bipyridine moieties. In contrast, persistent charge‐separated species located at the acridine moiety are formed upon excitation of COFs that have *β*‐ketoenamine connections. This was shown to be beneficial in the diffusion limited activation of homogeneous nickel complexes. A multicomponent COF that hosts nickel atoms was further shown to be a recyclable heterogeneous C−S cross‐coupling catalyst that can be activated with long wavelengths.

## Experimental Section

**Synthesis of Acr^x^‐L‐Bpy^y^ COFs (x : y=2:1 or 1:2 and L=Tp, DHTA, HTA or Tf)**: A typical COF synthesis is exemplified for Acr^2^‐Tf‐Bpy^1^. A Pyrex tube (o.d. × i.d.=15×10 mm^2^ and length 15 cm) is charged with 1,3,5‐triformylbenzene (Tf) (16.2 mg, 0.1 mmol), 2,6‐diaminoacridine (Acr) (20.9 mg, 0.1 mmol), 2,2′‐bipyridine‐5,5′‐diamine (Bpy) (9.3 mg, 0.05 mmol), 1.5 mL of *n*‐BuOH, 1.5 mL of anhydrous *o*‐DCB and 0.5 mL of 6 M aqueous acetic acid. This mixture was sonicated for 15 minutes in order to get a homogenous dispersion. The tube was then flash frozen at 77 K (liquid N_2_ bath) and degassed by three freeze‐pump‐thaw cycles. The tube was sealed off and then heated at 120 °C for 3 days. A dark red colored precipitate was collected by filtration and washed with acetone, methanol and cyclohexane. The powder collected was dried at 120 °C to give a dark red colored powder.

**General experimental procedure for photocatalytic experiments**: An oven dried vial (19×100 mm) equipped with a stir bar was charged with NiCl_2_⋅glyme (4–12 μmol), Acr^x^‐L‐Bpy^y^ COF, 4‐halobenzotrifluoride and nucleophile. The solvent (anhydrous) was added and the vessel was sealed with a septum and Parafilm. The mixture was stirred for 1 minute at high speed, followed by sonication for 5 minutes and degassing by bubbling argon for 10 minutes. The reaction mixture was stirred at 800 rpm and irradiated with 440 nm, 535 nm or 666 nm LED lamps. After the respective reaction time, 1,3,5‐trimethoxybenzene (1 eq.) was added as internal standard to the reaction vessel, the mixture was shaken and an aliquot (200 μL) was removed, filtered, diluted with DMSO‐d_6_ and analyzed by ^1^H NMR.

## Conflict of interest

The authors declare no conflict of interest.

1

## Supporting information

As a service to our authors and readers, this journal provides supporting information supplied by the authors. Such materials are peer reviewed and may be re‐organized for online delivery, but are not copy‐edited or typeset. Technical support issues arising from supporting information (other than missing files) should be addressed to the authors.

Supporting Information

## Data Availability

The data that support the findings of this study are available from the corresponding author upon reasonable request.
