# Supplementary material for: Programmable Photocatalytic Activity of Multicomponent Covalent Organic Frameworks Used as Metallaphotocatalysts
Source: Chemistry. 2022 Dec 1;29(4):e202202967. doi: 10.1002/chem.202202967 (PMC10108091; doi:10.1002/chem.202202967)
Supplement: Supplementary file 1 — Supporting Information [file CHEM-29-0-s001.pdf]

Chemistry–A European Journal

Supporting Information

Programmable Photocatalytic Activity of Multicomponent Covalent Organic Frameworks Used as Metallaphotocatalysts

Michael Traxler, Susanne Reischauer, Sarah Vogl, Jérôme Roeser, Jabor Rabeah, Christopher Penschke, Peter Saalfrank, Bartholomäus Pieber,* and Arne Thomas*

Table of Contents

Table of Contents 2
S1. General Remarks 4
S2. Setup for photochemical reactions 6
S3. Synthesis of organic linkers and COFs 8
S3.1 Synthesis of the organic linkers 8
S3.2 Synthesis of multicomponent covalent organic frameworks (COFs) 12
S4. Characterization multicomponent COFs 15
S4.1 PXRD analysis of Acr^x-L-Bpy^y COFs (x:y = 2:1 or 1:2; L = Tp, DHTA, HTA, Tf)...15
S4.2 Nitrogen sorption experiments for Acr^1-L-Bpy^2 multicomponent COFs 16
S4.3 Pore size distribution for Acr^2-L-Bpy^1 multicomponent COFs 16
S4.4 FT-IR results of of Acr^x-L-Bpy^y COFs 17
S4.5 Thermogravimetric analyses of Acr^x-L-Bpy^y COFs 19
S4.6 Solid-state NMR 19
S4.7 X-ray photoelectron spectroscopy (XPS) analysis of Acr^x-L-Bpy^y COFs 20
S4.8 COF digestion of Acr^x-Tp-Bpy^y 20
S4.9 UV-vis of Acr^x-L-Bpy^x COFs 21
S4.10 Comparison of theoretical and experimental excitation energies and band gaps 22
S4.11 Photoluminescence measurements of Acr^2-L-Bpy^1 multicomponent COFs 25
S4.12 Fluorescence quenching studies of Acr^2-Tf-Bpy^1 multicomponent COF 26
S4.13 Electron paramagnetic resonance (EPR) spin trap experiments of Acr^2-Tf-$[Ni]^1$ and Acr^2-Tp-$[Ni]^1$ multicomponent COFs 26
S5. Synthesis and characterization of reference COFs 29
S5.1 Synthesis of bipyridine COFs 29
S5.2 Characterization of bipyridine COFs 30
S5.3 Synthesis of Tf-Acr COF 33
S5.4 Characterization of Tf-Acr COF 33
S6. Synthesis and characterization of model compounds 34
S6.1 Synthesis of acridine model compound (SA-Acr) 34
S6.2 Synthesis of bipyridine model compounds (BA-Bpy) 35
S7. Photocatalytic reaction optimization 36
S7.1 General experimental procedure for screening experiments 36
S7.2 Initial screening experiments using sodium *p*-toluensulfinate 36
S7.3 Screening experiments for different COFs 37
S7.4 Screening different COFs for cross-coupling of aryl halides and nucleophiles 38
S7.5 Control studies 39
S8. Photocatalysis - recycling studies 41

S8.1 Reaction procedure 41
S8.2 Analysis of recycled Tp-Acr COF 42
S10. References 46
S11. Author Contributions 47
S12. Copies of NMR spectra...48

S1. General Remarks

All air and moisture sensitive reactions were performed using standard Schlenk-line techniques under an atmosphere of argon. Substrates, reagents, and solvents were purchased from commercial suppliers and used without further purification. The precursors such as phloroglucinol (TCI, > 99 %), resorcinol (> 98.5 %), phenol (TCI, > 99 %), 1,3,5-triformylbenzene (TCI, > 98 %), 2,2'-bipyridine-5-5'-diamine (BLDpharm, 97 %), 1-chloro-3-nitrobenzene (TCI, > 99 %), 4-nitroaniline (Carl Roth, > 98.5 %), 2,6-diaminoanthraquinone (TCI, > 97 %) and salicylic aldehyde (Sigma Aldrich, 98 %) were purchased and used as received. Reactants and solvents were obtained from Sigma Aldrich ((2-biphenyl)dicyclohexylphosphine (CyJohnPhos, 97 %), 1,2-dimethoxyethane (DME, 99.5 %, anhydrous), tin (powder <150 µm, 99.5 %), mesitylene (1,3,5-trimethylbenzene, 98 %), 1,2-dichlorobenzene (*o*-DCB, 99 %, anhydrous), glycerol (99.5 %)), TCI (tris(dibenzylideneacetone)dipalladium(0) ($Pd_2(dba)_3$, > 75 %), hexamethylenetetramine (HMTA, > 99 %)), ABCR (tripotassium phosphate (K_3PO_4, 97 %), palladium (Pd/C, 10 % on activated charcoal), trifluoroacetic acid (TFA, 99.9 %), 1,4-dioxane (99.5 %)), Carl Roth (formic acid (> 98 %), hydrochloric acid (37 %), sulfuric acid (96 %)), Alphagaz (hydrogen gas (99.999 %)), Eurisotop ($CDCl_3$ (99.8 $\%_d$), DMSO-d_6 (99.8 $\%_d$)), Chemsolute (sodium hydroxide (NaOH, 99.5 %)), Fluka Analytical (sodium borohydride (> 99 %)) or Grüssing (1-butanol (*n*-BuOH, 99.5 %)). Powder X-ray diffraction data were collected on a Bruker D8 Advance diffractometer in reflection geometry operating with a Cu K_α anode (λ = 1.54178 Å) with a working voltage at 40 kV and a current of 40 mA. Samples were ground and mounted as loose powders onto a Si sample holder. PXRD patterns were collected from 2 to 60 2θ degrees with a step size of 0.02 degrees and an exposure time of 2 seconds per step. LED lamps for photocatalytic experiments were purchased from Kessil Lightning.[1] ^{1}H-, ^{13}C-, and ^{19}F spectra were recorded on a Varian 400 spectrometer (400 MHz, Agilent), an Ascend™ 400 spectrometer (400 MHz, cryoprobe, Bruker), a Varian 600 spectrometer (600 MHz, Agilent), a Bruker Avance II spectrometer (200 MHz, Bruker) or an Bruker Avance 400 spectrometer (400 MHz, Bruker) at 298 K, and are reported in ppm relative to the residual solvent peaks. Peaks are reported as: s = singlet, d = doublet, t = triplet, q = quartet, m = multiplet or unresolved, with coupling constants in Hz. ^{13}C{1H} cross polarization magic angle spinning (CP/MAS) measurements were carried out using a Bruker range Avance 400 MHz Solid State spectrometer operating at 100.6 MHz and a Bruker 4 mm double resonance probe-head operating at a spinning rate of 10 kHz. Nitrogen sorption measurements were performed at 77 K using an

Autosorb-iQ-MP or QuadraSorb from Quantachrome. Prior the analysis the samples were dried and degassed at 150 °C for 12 h. Using the N_2 adsorption isotherms, the surface areas were calculated over a pressure range 0.05-0.1 = p/p0 using Brunauer-Emmett-Teller (BET) methods. The pore size distributions were calculated from the adsorption isotherms by Quenched Solid State Functional Theory (QSDFT) using N_2 sorption data collected at 77 K. We used the carbon cylindrical pore model for analyzing the distribution. Thermogravimetric analysis (TGA) measurements were carried out under nitrogen atmosphere on a Mettler Toledo TGA 1 Stare thermal instrument with a heating rate of 10 K min^{-1}. Solid state diffuse reflectance ultraviolet-visible spectroscopy (UV-vis) spectra have been collected on a Varian Cary 300 UV-Vis Spectrophotometer. UV-vis absorption spectra of COF suspensions were collected using a Shimadzu UV-1900. Fluorescence spectra were measured using a microplate reader (SpectraMax M5, Molecular Devices) or FluoroMax-2 (ISA Instruments S.A., Inc.). The Fourier transform infrared spectroscopy (FTIR) analyses of the samples were carried on Varian 640IR spectrometer equipped with an ATR cell. The TEM images were acquired using a Jeol TEM-F200, for the investigation, the acceleration voltage was set to 80 kV, the emission was put to 115,8 µA and a condenser aperture with a diameter of 200 µm was used. The specimens were prepared by dissolving a powder sample of the material in ethanol, sonicating the solution for 15 minutes and finally dropping a few drops onto a copper TEM grid coated with holey carbon film. Once the solution had dried off, the specimens were investigated. Scanning electron microscopy (SEM) images were obtained on a LEO 1550-Gemini microscope. Energy-dispersive X-ray (EDX) investigations were conducted on a Link ISIS-300 system (Oxford Microanalysis Group) equipped with a Si(Li) detector and an energy resolution of 133 eV. X-Ray photoelectron spectra were measured on a K-Alpha™ + X-ray Photoelectron Spectrometer System (Thermo Scientific) with Hemispheric 180° dual-focus analyzer with 128-channel detector. The X-ray monochromator used micro focused Al-Kα radiation. High resolution mass spectrometry (HR-MS) was performed on an LTQ Orbitrap XL spectrometer using electronspray ionization (ESI) or atmospheric pressure chemical ionization (APCI). Inductively coupled plasma - optical emission spectrometry (ICP-OES) was carried out using a Horiba Ultra 2 instrument equipped with a photomultiplier tube detection system. Analytical thin layer chromatography (TLC) was performed on pre-coated TLC-sheets, ALUGRAM Xtra SIL G/UV254 sheets (Macherey-Nagel) and visualized with 254 nm light or staining solutions followed by heating. Purification of final compounds was carried out by flash chromatography using a Biotage Isolera automatic column system with pre-packed silica columns. Centrifugation was carried out using an Eppendorf 5430 centrifuge.

S2. Setup for photochemical reactions

Photochemical experiments involving visible light irradiation were carried out using Kessil PR160L-440 (440nm, blue light) or Kessil PR160L-525 (525 nm, green light) LED lamps with the respective power settings.[1] One or two lamps were used, depending on the required light intensity to irradiate reaction vessels located on a stirring plate (lamp-vessel distance: 4.5 cm; stirring speed: 800 rpm, Figure S2). To avoid heating of the reaction mixture, fans were used for cooling.

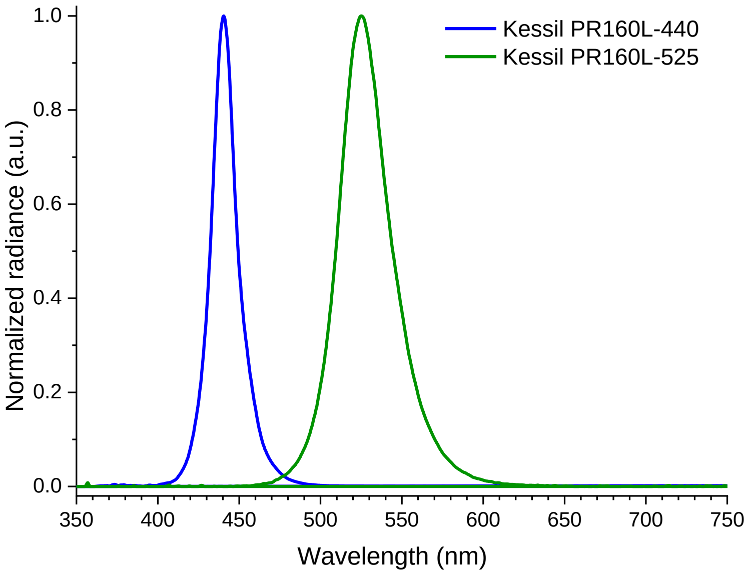

Figure S 1. Emission spectra of Kessil PR160L-400 (blue) and Kessil PR160L-525 (green).

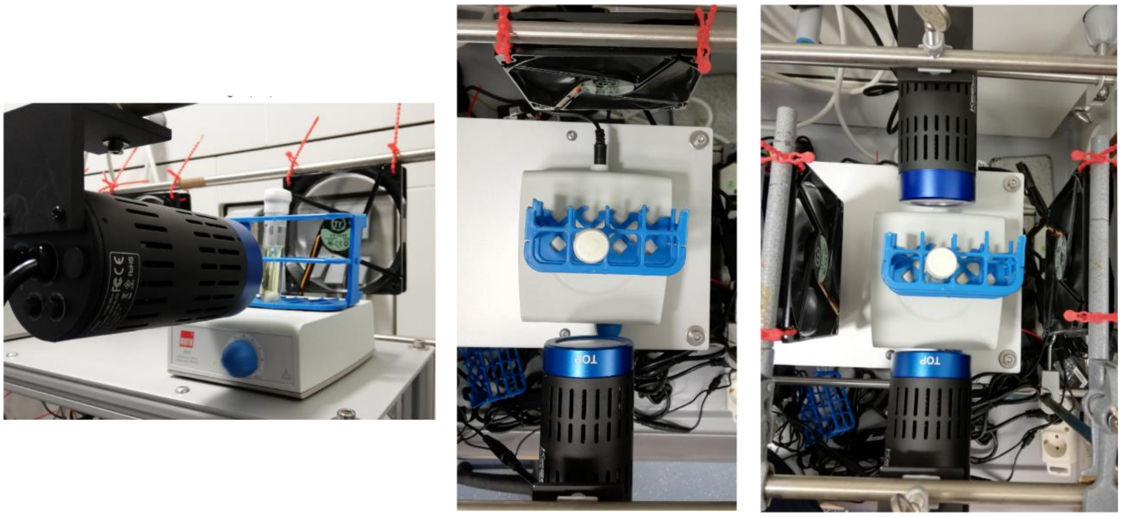

Figure S 2. Configuration of the experimental setup using one or two LED lamps.

Experiments using red light were carried out using a Kessil H160 Tuna Flora LED in “red” mode (Figure S3). Two sealed reaction vessels were placed between two lamps on a stirring plate (4.5 cm distance from each lamp). To avoid heating of the reaction mixture, a fan was used for cooling. All reactions were performed with maximum stirring speed.

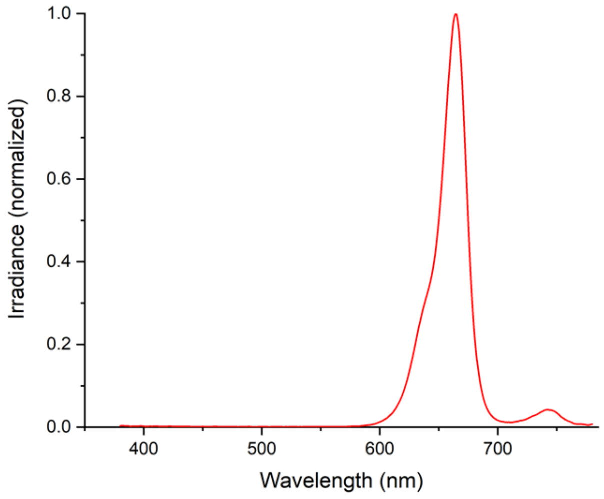

Figure S 3 . Emission spectra of the Kessil H160 Tuna Flora LED in “red” mode.

S3. Synthesis of organic linkers and COFs

S3.1 Synthesis of the organic linkers

Synthesis of 2,6-diaminoacridine:

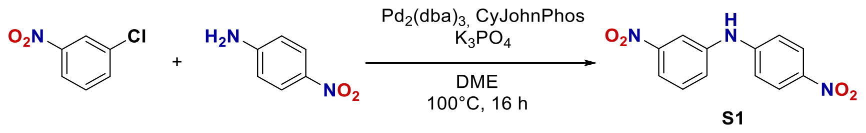

Scheme S 1. Synthesis of 3-nitro-*N*-(4-nitrophenyl)aniline (S1) via Buchwald-Hartwig coupling.

3-nitro-*N*-(4-nitrophenyl)aniline (S1): To a flame dried and three times evacuated and backfilled Schlenk flask were under argon counterflow 206 mg (0.22 mmol, 3 mol%) of $Pd_2(dba)_3$ and 80 mg (2-biphenyl)dicyclohexylphosphine (CyJohnPhos, 0.22 mmol, 3 mol%) added. 15 mL of anhydrous 1,2-dimethoxyehtane (DME) were added and the resulting suspension was stirred for 5 min at room temperature. Subsequently, 1.18 g (7.5 mmol, 1.0 eq.) of 1-chloro-3-nitrobenzene, 1.03 g (7.5 mmol, 1.0 eq.) of 4-nitroaniline and 2.23 g (10.5 mmol, 1.4 eq.) of K_3PO_4 were added and the mixture was heated to 100 °C for 24 h while stirring was maintained. After cooling to room temperature, the suspension was diluted with 300 mL of Et_2O/EtOAc (1:1), filtered through Celite and concentrated using the rotary evaporator. The crude material was purified by column chromatography on silica gel using 30 % EtOAc in cyclohexane to get the product as an orange powder (1.85 g, 95%).

^{1}H NMR (400 MHz, DMSO-d_6) δ 9.67 (s, 1H), 8.16 (d, J = 9.2 Hz, 2H), 7.86 (ddd, J = 7.8, 2.2, 1.2 Hz, 1H), 7.68 (ddd, J = 8.1, 2.1, 1.2 Hz, 1H), 7.63 (t, J = 8.0 Hz, 1H), 7.21 (d, J = 9.3 Hz, 2H). ^{13}C NMR (101 MHz, DMSO-d_6) δ 149.13, 148.66, 141.91, 139.37, 130.90, 126.10, 125.40, 116.86, 114.83, 113.41.

These data are in full agreement with those previously published in the literature.[2]

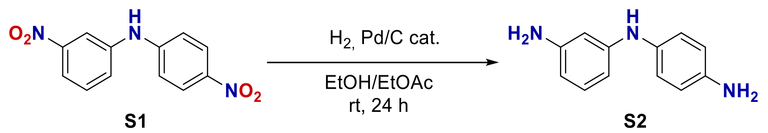

Scheme S 2. Synthesis of 3-(4-aminoanilino)aniline (S2) from 3-nitro-*N*-(4-nitrophenyl)aniline (S1).

3-(4-aminoanilino)aniline (S2): A suspension of 1.66 g (6.4 mmol) of 3-nitro-*N*-(4-nitrophenyl)aniline (**S1**), 332 mg (0.31 mmol, 4.9 mol%) of Pd/C (10 wt% Pd), 200 mL EtOH

and 100 mL EtOAc were degassed for 15 min using an argon purge. Subsequently, the suspension was degassed for 10 min using and H_2 purge and thereafter stirred for 24 h under hydrogen atmosphere. The catalyst was removed by filtration through Celite and the solvent was concentrated under vacuum and dried at 65 °C under high vacuum to give a dark brown solid (1.23 g, 96 %).

^{1}H NMR (200 MHz, DMSO-*d*$_6$) δ 7.11 (s, 1H), 6.83 – 6.68 (m, 3H), 6.51 (d, *J* = 8.6 Hz, 2H), 6.06 (t, *J* = 2.0 Hz, 1H), 5.99 (ddd, *J* = 7.9, 2.1, 0.8 Hz, 1H), 5.88 (ddd, *J* = 7.8, 2.0, 0.9 Hz, 1H), 4.74 (bs, 4H). ^{13}C NMR (50 MHz, DMSO-*d*$_6$) δ 149.20, 147.28, 143.21, 132.35, 129.14, 122.39, 114.69, 104.20, 103.03, 99.56.

These data are in full agreement with those previously published in the literature.[2]

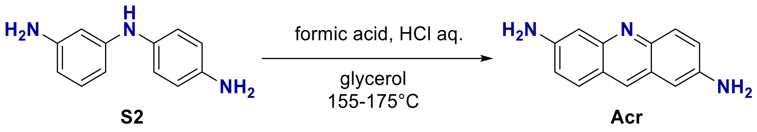

Scheme S 3. Synthesis of 2,6-diaminoacridine (Acr) from 3-(4-aminoanilino)aniline (S2).

2,6-diaminoacridine (Acr): To 1.23 g (6.1 mmol, 1.0 eq.) of 3-(4-aminoanilino)aniline (**S2**) were 4 mL glycerol, 235 µl (6.1 mmol, 1.0 eq.) of formic acid and 660 µl (7.9 mmol, 1.3 eq.) of 37 % aqueous HCl solution added. The reaction mixture was heated to 155 °C during 30 min, kept at this temperature for 30 min before heating it to 175 °C for another 30 min. After cooling to room temperature 2.5 mL of aqueous sulfuric acid (30 $\%_{w/v}$) were added and the reaction was heated to 95 °C for 10 min. Subsequently, the reaction was diluted to 25 mL with H_2O and the reaction mixture was kept at 0 °C for 1 h. The precipitated acid sulfate of the compound was filtered off, washed with water (40 mL) and Et_2O (60 mL). The dark red powder was boiled in 10 mL of aqueous NaOH (1 M) to precipitate the 2,6-diaminoacridine. The dark brown compound was filtered and washed with ice cold water (60 mL) and cold Et_2O (10 mL) and dried under vacuum at 65 °C (407 mg, 32 %).

^{1}H NMR (400 MHz, DMSO-*d*$_6$) δ 8.35 – 8.24 (m, 1H), 7.67 (dd, *J* = 9.0, 2.9 Hz, 2H), 7.22 (dd, *J* = 9.2, 2.3 Hz, 1H), 6.99 (dd, *J* = 9.0, 2.0 Hz, 1H), 6.84 – 6.82 (m, 1H), 6.82 – 6.80 (m, 1H), 5.83 – 5.78 (m, 1H), 5.43 – 5.39 (m, 1H). ^{13}C NMR (101 MHz, DMSO-*d*$_6$) δ 148.54, 148.07, 144.44, 144.07, 130.64, 128.51, 128.23, 125.84, 124.52, 121.45, 120.55, 103.65, 103.48. HR-ESI-MS [M+H^{+}] (m/z)): 210.1028 (th.: 210.1026).

Synthesis of aldehyde linkers:

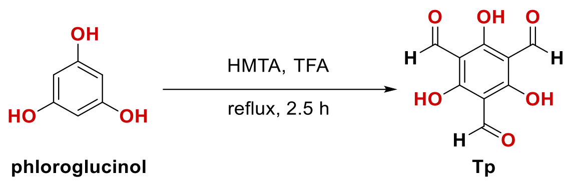

Scheme S 4. Synthesis of 1,3,5-triformylphloroglucino (Tp) from phloroglucinol.

1,3,5-triformylphloroglucinol (Tp): To 10.0 g (80 mmol, 1.0 eq.) of phloroglucinol and 25.0 g (179 mmol, 2.2 eq.) of hexamethylenetetramine (HMTA) were 150 mL trifluoroacetic acid (TFA) added slowly under argon atmosphere at 0 °C. After complete addition, the suspension was heated at 100 °C for 2.5 h. The reaction mixture was cooled to around 50 °C and 240 mL of 3 M HCl were added, and the solution was heated at 100 ºC for 1 h. After cooling to room temperature, the solution was filtered through Celite, extracted with 3x 200 mL dichloromethane, dried over magnesium sulfate, and filtered. Rotary evaporation of the solution afforded of an off-white powder. A pure sample was obtained by washing the solid sample with 10 mL of cold EtOH followed by sublimation under reduced pressure (2.76 g, 16 %).

^{1}H NMR (200 MHz, $CDCl_3$) δ 14.11 (s, 1H), 10.14 (s, 1H). ^{13}C NMR (50 MHz, $CDCl_3$) δ 192.17, 173.71, 103.05.

These data are in full agreement with those previously published in the literature.[3]

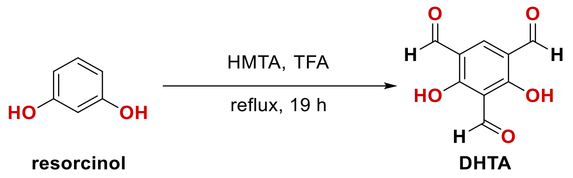

Scheme S 5. Synthesis of 2,4-dihydroxybenzene-1,3,5-tricarbaldehyde (DHTA) from resorcinol.

2,4-dihydroxybenzene-1,3,5-tricarbaldehyde (DHTA): To 3.6 g (33 mmol, 1.0 eq.) of resorcinol and 10.0 g (71 mmol, 2.2 eq.) of hexamethylenetetramine (HMTA) were 35 mL trifluoroacetic acid added slowly under argon atmosphere at 0 °C. After complete addition, the suspension was heated at 130 °C for 16 h and afterwards 3 h at 150 °C. The reaction mixture was cooled to around 100 °C and 55 mL of 3 M HCl were added, and the solution was heated at 105 ºC for 30 min. After cooling to room temperature, the solution was filtered through Celite, extracted with 3x 50 mL dichloromethane, dried over magnesium sulfate, and filtered. Rotary evaporation of the solution afforded of an off-white powder. A pure sample was

obtained by washing the solid sample with 10 mL of cold EtOH followed by sublimation under reduced pressure (1.37 g, 21 %).

^{1}H NMR (400 MHz, DMSO-d_6) δ 10.26 (s, 1H), 10.09 (s, 2H), 8.40 (s, 1H). ^{13}C NMR (101 MHz, DMSO-d_6) δ 193.93, 189.97, 169.97, 140.66, 115.80, 109.95.

These data are in full agreement with those previously published in the literature.[4]

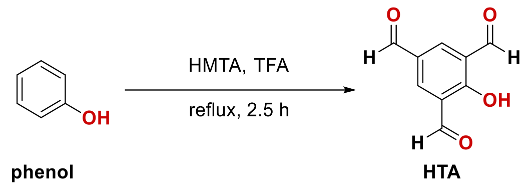

Scheme S 6. Synthesis of 2-hydroxybenzene-1,3,5-tricarbaldehyde (HTA) from phenol.

2-hydroxybenzene-1,3,5-tricarbaldehyde (HTA): To 3.5 g (36.7 mmol, 1.0 eq.) of phenol and 10.1 g (71.3 mmol, 2.0 eq.) of hexamethylenetetramine (HMTA) were 50 mL trifluoroacetic acid added slowly under argon atmosphere at 0 °C. After complete addition, the suspension was heated at 120 °C for 20 h and afterwards 30 min at 150 °C. The reaction mixture was cooled to around 120 °C and 50 mL of 3 M HCl were added, and the solution was heated at 120 ºC for 30 min. While cooling to room temperature a yellow precipitate was formed, which was filtered, washed with 20 mL of cold EtOH and dried under vacuo to give an off-white solid (3.64 g, 56%). Pure sample was obtained by sublimation of the crude material under reduced pressure.

^{1}H NMR (200 MHz, DMSO-d_6) δ 10.30 (s, 2H), 9.99 (s, 1H), 8.52 (s, 2H). ^{1}H NMR (200 MHz, $CDCl_3$) δ 12.16 (s, 1H), 10.32 (s, 2H), 10.01 (s, 1H), 8.51 (s, 2H). ^{13}C NMR (50 MHz, DMSO-d_6) δ 191.65, 190.72, 165.88, 137.31, 128.30, 124.09.

These data are in full agreement with those previously published in the literature.[5]

S3.2 Synthesis of multicomponent covalent organic frameworks (COFs)

Acr^2-Tp-Bpy^1 COF: A Pyrex tube (o.d. × i.d. = 15 × 10 mm^2 and length 15 cm) is charged with triformylphloroglucinol (**Tp**) (21 mg, 0.1 mmol), 2,6-diaminoacridine (**Acr**) (20.9 mg, 0.1 mmol), 2,2′-bipyridine-5,5′-diamine (**Bpy**) (9.3 mg, 0.05 mmol), 1.5 mL of *n*-BuOH, 1.5 mL of anhydrous *o*-DCB and 0.5 mL of 6 M aqueous acetic acid. This mixture was sonicated for 15 minutes in order to get a homogenous dispersion. The tube was then flash frozen at 77 K (liquid N_2 bath) and degassed by three freeze-pump-thaw cycles. The tube was sealed off and then heated at 120 °C for 3 days. A dark red colored precipitate was collected by filtration and washed with acetone, methanol and cyclohexane. The powder collected was dried at 120 °C to give a dark red colored powder (44 mg, 96 %).

Acr^1-Tp-Bpy^2 COF: A Pyrex tube (o.d. × i.d. = 15 × 10 mm^2 and length 15 cm) is charged with triformylphloroglucinol (**Tp**) (21 mg, 0.1 mmol), 2,6-diaminoacridine (**Acr**) (10.4 mg, 0.05 mmol), 2,2′-bipyridine-5,5′-diamine (**Bpy**) (18.6 mg, 0.1 mmol), 1.5 mL of *n*-BuOH, 1.5 mL of anhydrous *o*-DCB and 0.5 mL of 6 M aqueous acetic acid. This mixture was sonicated for 15 minutes in order to get a homogenous dispersion. The tube was then flash frozen at 77 K (liquid N_2 bath) and degassed by three freeze-pump-thaw cycles. The tube was sealed off and then heated at 120 °C for 3 days. A dark red colored precipitate was collected by filtration and washed with acetone, methanol and cyclohexane. The powder collected was dried at 120 °C to give a dark red colored powder (38 mg, 86 %).

Acr^2-DHTA-Bpy^1 COF: A Pyrex tube (o.d. × i.d. = 15 × 10 mm^2 and length 15 cm) is charged with 2,4-dihydroxybenzene-1,3,5-tricarbaldehyde (**DHTA**) (19.4 mg, 0.1 mmol), 2,6-diaminoacridine (**Acr**) (20.9 mg, 0.1 mmol), 2,2′-bipyridine-5,5′-diamine (**Bpy**) (9.3 mg, 0.05 mmol), 1.5 mL of *n*-BuOH, 1.5 mL of anhydrous *o*-DCB and 0.5 mL of 6 M aqueous acetic acid. This mixture was sonicated for 15 minutes in order to get a homogenous dispersion. The tube was then flash frozen at 77 K (liquid N_2 bath) and degassed by three freeze-pump-thaw cycles. The tube was sealed off and then heated at 120 °C for 3 days. A dark red colored precipitate was collected by filtration and washed with acetone, methanol and cyclohexane. The powder collected was dried at 120 °C to give a dark red colored powder (43 mg, 98 %).

Acr^1-DHTA-Bpy^2 COF: A Pyrex tube (o.d. × i.d. = 15 × 10 mm^2 and length 15 cm) is charged with 2,4-dihydroxybenzene-1,3,5-tricarbaldehyde (**DHTA**) (19.4 mg, 0.1 mmol), 2,6-diaminoacridine (**Acr**) (10.4 mg, 0.05 mmol), 2,2′-bipyridine-5,5′-diamine (**Bpy**) (18.6 mg, 0.1 mmol), 1.5 mL of *n*-BuOH, 1.5 mL of anhydrous *o*-DCB and 0.5 mL of 6 M aqueous acetic acid. This mixture was sonicated for 15 minutes in order to get a homogenous dispersion. The tube was then flash frozen at 77 K (liquid N_2 bath) and degassed by three freeze-pump-thaw cycles. The tube was sealed off and then heated at 120 °C for 3 days. A dark red colored precipitate was collected by filtration and washed with acetone, methanol and cyclohexane. The powder collected was dried at 120 °C to give a dark red colored powder (38 mg, 89 %).

Acr^2-HTA-Bpy^1 COF: A Pyrex tube (o.d. × i.d. = 15 × 10 mm^2 and length 15 cm) is charged with 2-hydroxybenzene-1,3,5-tricarbaldehyde (**HTA**) (17.8 mg, 0.1 mmol), 2,6-diaminoacridine (**Acr**) (20.9 mg, 0.1 mmol), 2,2′-bipyridine-5,5′-diamine (**Bpy**) (9.3 mg, 0.05 mmol), 1.5 mL of *n*-BuOH, 1.5 mL of anhydrous *o*-DCB and 0.5 mL of 6 M aqueous acetic acid. This mixture was sonicated for 15 minutes in order to get a homogenous dispersion. The tube was then flash frozen at 77 K (liquid N_2 bath) and degassed by three freeze-pump-thaw cycles. The tube was sealed off and then heated at 120 °C for 3 days. A dark red colored precipitate was collected by filtration and washed with acetone, methanol and cyclohexane. The powder collected was dried at 120 °C to give a dark red colored powder (41 mg, 97 %).

Acr^1-HTA-Bpy^2 COF: A Pyrex tube (o.d. × i.d. = 15 × 10 mm^2 and length 15 cm) is charged with 2-hydroxybenzene-1,3,5-tricarbaldehyde (**HTA**) (17.8 mg, 0.1 mmol), 2,6-diaminoacridine (**Acr**) (10.4 mg, 0.05 mmol), 2,2′-bipyridine-5,5′-diamine (**Bpy**) (18.6 mg, 0.1 mmol), 1.5 mL of *n*-BuOH, 1.5 mL of anhydrous *o*-DCB and 0.5 mL of 6 M aqueous acetic acid. This mixture was sonicated for 15 minutes in order to get a homogenous dispersion. The tube was then flash frozen at 77 K (liquid N_2 bath) and degassed by three freeze-pump-thaw cycles. The tube was sealed off and then heated at 120 °C for 3 days. A dark red colored precipitate was collected by filtration and washed with acetone, methanol and cyclohexane. The powder collected was dried at 120 °C to give a dark red colored powder (34 mg, 89 %).

Acr²-Tf-Bpy¹ COF: A Pyrex tube (o.d. × i.d. = 15 × 10 mm^2 and length 15 cm) is charged with 1,3,5-triformylbenzene (**Tf**) (16.2 mg, 0.1 mmol), 2,6-diaminoacridine (**Acr**) (20.9 mg, 0.1 mmol), 2,2′-bipyridine-5,5′-diamine (**Bpy**) (9.3 mg, 0.05 mmol), 1.5 mL of *n*-BuOH, 1.5 mL of anhydrous *o*-DCB and 0.5 mL of 6 M aqueous acetic acid. This mixture was sonicated for 15 minutes in order to get a homogenous dispersion. The tube was then flash frozen at 77 K (liquid N_2 bath) and degassed by three freeze-pump-thaw cycles. The tube was sealed off and then heated at 120 °C for 3 days. A dark red colored precipitate was collected by filtration and washed with acetone, methanol and cyclohexane. The powder collected was dried at 120 °C to give a dark red colored powder (37 mg, 91 %).

Acr¹-Tf-Bpy² COF: A Pyrex tube (o.d. × i.d. = 15 × 10 mm^2 and length 15 cm) is charged with 1,3,5-triformylbenzene (**Tf**) (16.2 mg, 0.1 mmol), 2,6-diaminoacridine (**Acr**) (10.4 mg, 0.05 mmol), 2,2′-bipyridine-5,5′-diamine (**Bpy**) (18.6 mg, 0.1 mmol), 1.5 mL of *n*-BuOH, 1.5 mL of anhydrous *o*-DCB and 0.5 mL of 6 M aqueous acetic acid. This mixture was sonicated for 15 minutes in order to get a homogenous dispersion. The tube was then flash frozen at 77 K (liquid N_2 bath) and degassed by three freeze-pump-thaw cycles. The tube was sealed off and then heated at 120 °C for 3 days. A dark red colored precipitate was collected by filtration and washed with acetone, methanol and cyclohexane. The powder collected was dried at 120 °C to give a dark red colored powder (33 mg, 81 %).

S4. Characterization multicomponent COFs

S4.1 PXRD analysis of Acr^x-L-Bpy^y COFs (x:y = 2:1 or 1:2; L = Tp, DHTA, HTA, Tf)

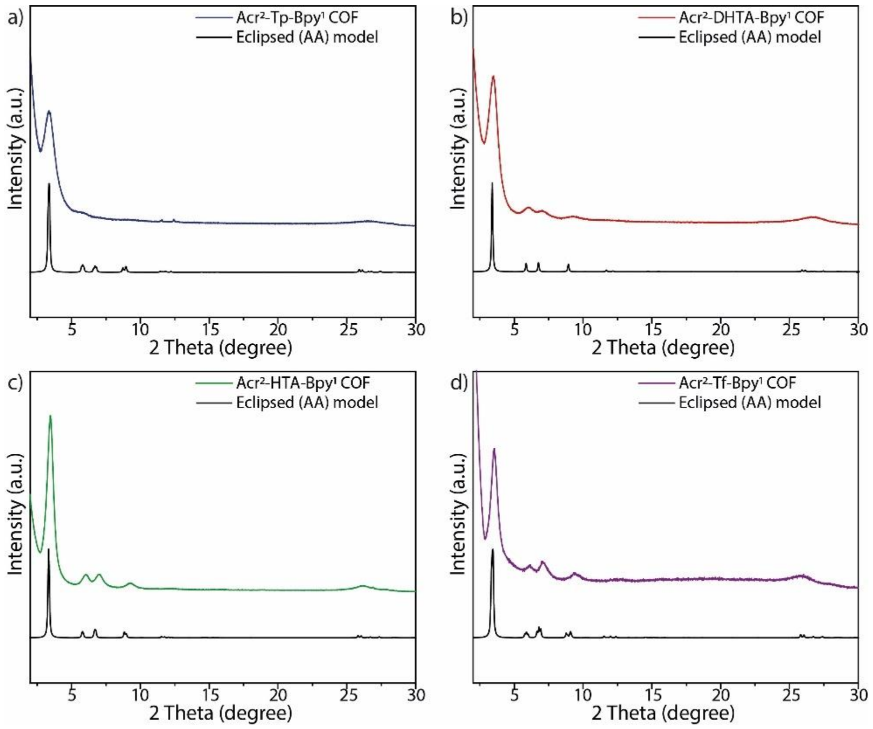

Figure S 4. Comparison between simulated and experimental PXRD patterns for a) Acr^2-Tp-Bpy^1, b) Acr^2-DHTA-Bpy^1, c) Acr^2-HTA-Bpy^1 and d) Acr^2-Tf-Bpy^1 COFs, showing agreement between the experimental diffractogram and the eclipsed stacking model (AA).

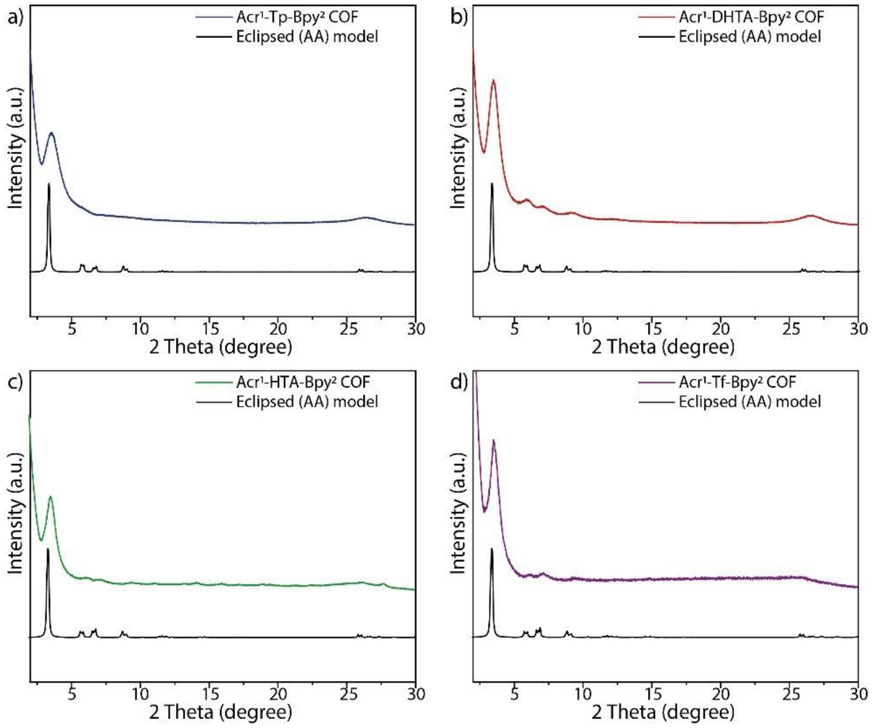

Figure S 5. Comparison between simulated and experimental PXRD patterns for a) Acr^1-Tp-Bpy^2, b) Acr^1-DHTA-Bpy^2, c) Acr^1-HTA-Bpy^2 and d) Acr^1-Tf-Bpy^2 COFs, showing agreement between the experimental diffractogram and the eclipsed stacking model (AA).

S4.2 Nitrogen sorption experiments for Acr¹-L-Bpy² multicomponent COFs

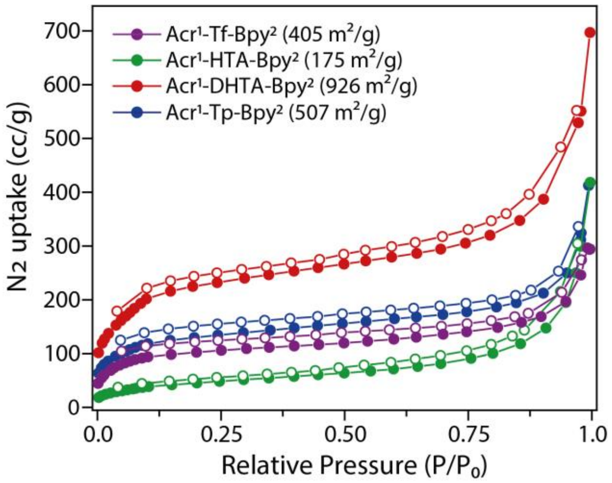

Figure S 6. N_2 **sorption isotherms for Acr¹-Tp-Bpy², Acr¹-DHTA-Bpy², Acr¹-HTA-Bpy² and Acr¹-Tf-Bpy² COFs, calculated BET surface areas are shown in brackets.**

S4.3 Pore size distribution for Acr²-L-Bpy¹ multicomponent COFs

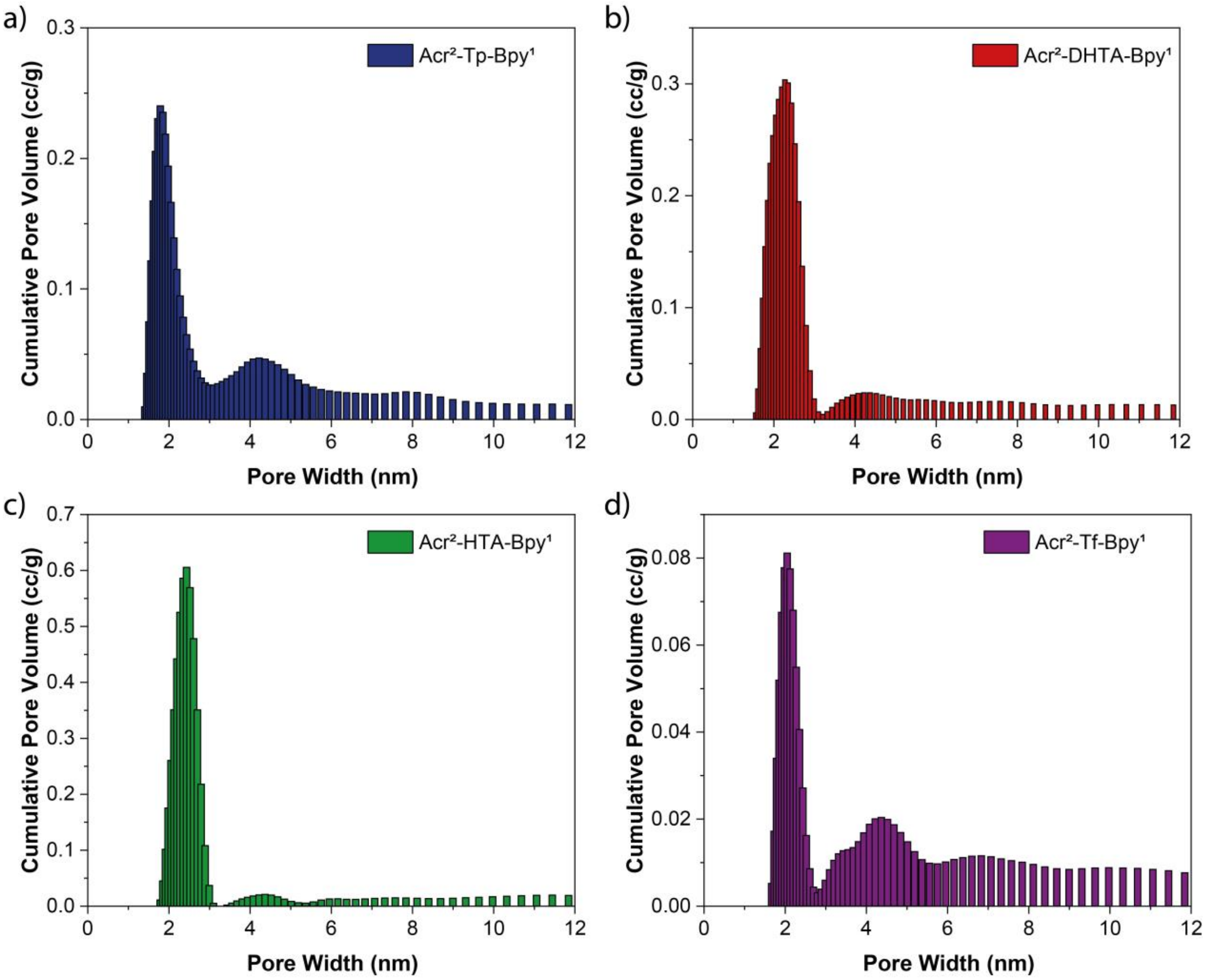

Figure S 7. Pore size distribution of a) Acr²-Tp-Bpy¹, b) Acr²-DHTA-Bpy¹, c) Acr²-HTA-Bpy¹ and d) Acr²-Tf-Bpy¹ COFs, showing the pores distribution close to the ideal pore size calculated from simulated structures.

Acr^2-Tp-Bpy^1 COF:

Method = Quenched Solid State Functional Theory (QSDFT)

Model = N_2 at 77 K on carbon cylindrical pore (fitting error = 1.1%)

Acr^2-DHTA-Bpy^1 COF:

Method = Quenched Solid State Functional Theory (QSDFT)

Model = N_2 at 77 K on carbon cylindrical pore (fitting error = 1.3%)

Acr^2-HTA-Bpy^1 COF:

Method = Quenched Solid State Functional Theory (QSDFT)

Model = N_2 at 77 K on carbon cylindrical pore (fitting error = 2.1%)

Acr^2-Tf-Bpy^1 COF:

Method = Quenched Solid State Functional Theory (QSDFT)

Model = N_2 at 77 K on carbon cylindrical pore (fitting error = 1.6%)

S4.4 FT-IR results of of Acr^x-L-Bpy^y COFs

Fourier transform infrared (FT-IR) spectra show the disappearance of the vibration of the amino group of the 2,6-diaminoacridine (**Acr**) and 2,2′-bipyridine-5,5′-diamine (**Bpy**) linker at around 3400 cm^{-1}, and of the stretching vibration of C=O groups of the aldehyde linker (1690-1640 cm^{-1}). The bands of the newly formed C=O and C=C and C=N bonds, respectively were merged into a peak in the range of 1630 cm^{-1} to 1560 cm^{-1} for the COFs, where the peak is at higher wavenumber for imine-based COFs. Distinctive spectral bands of the acridine linker at ~800 cm^{-1} and for the bipyridine unit at ~1250 cm^{-1} can be also found in the acridine COFs.

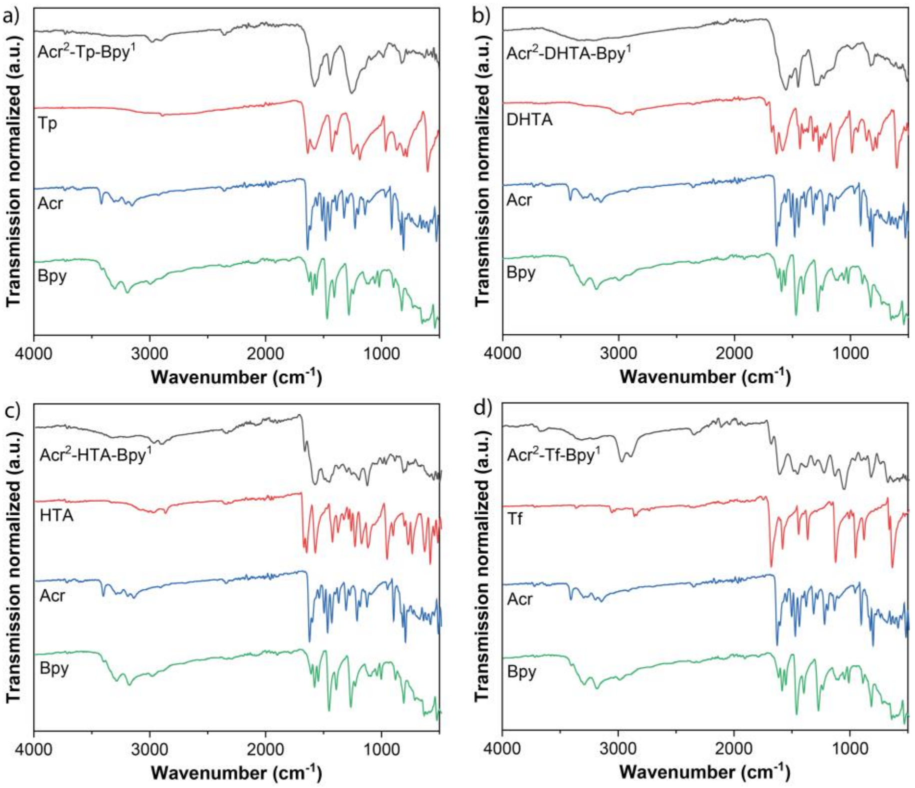

Figure S 8. FT-IR analyses of Acr^2-L-Bpy^1 COFs [L = Tp (a), DHTA (b), HTA (c), Tf (d)] in comparison with the corresponding aldehydes and amines (Acr, Bpy) showing the formation of the framework structure.

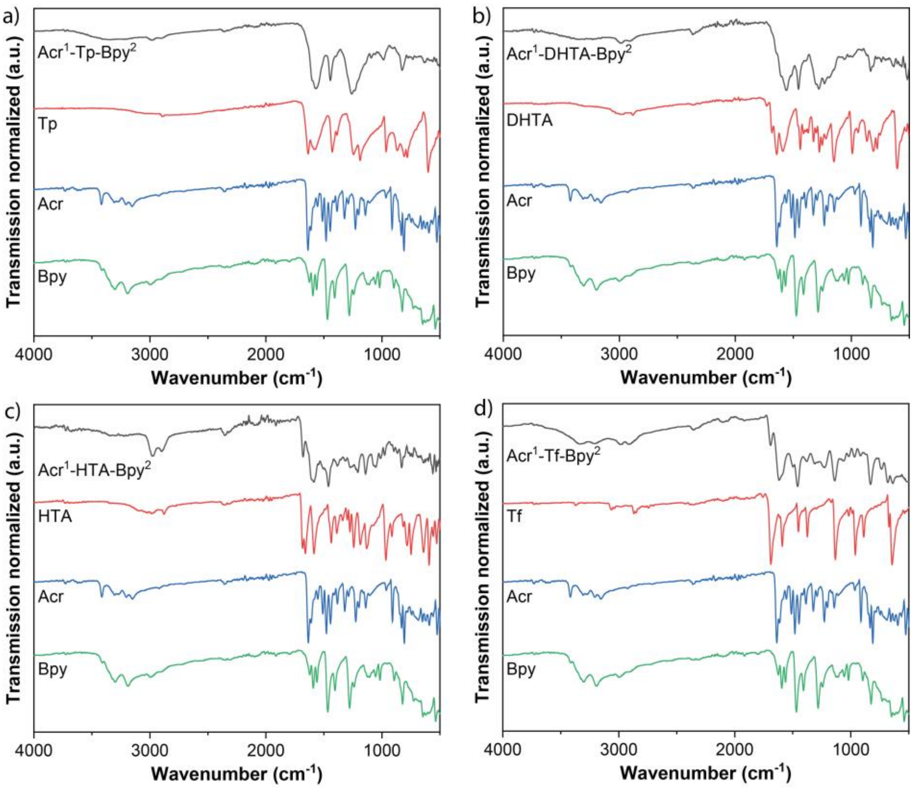

Figure S 9. FT-IR analyses of Acr^1-L-Bpy^2 COFs [L = Tp (a), DHTA (b), HTA (c), Tf (d)] in comparison with the corresponding aldehydes and amines (Acr, Bpy) showing the formation of the framework structure.

S4.5 Thermogravimetric analyses of Acr^{x}-L-Bpy^{y} COFs

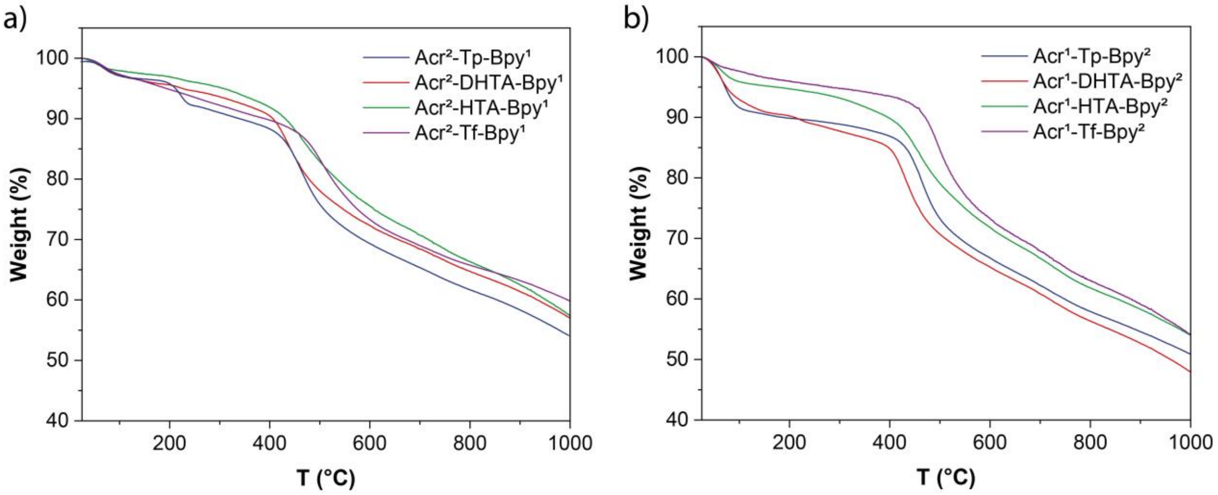

Figure S 10. Thermogravimetric analyses (TGA) for a) Acr^{2}-L-Bpy^{1} and b) Acr^{1}-L-Bpy^{2} COFs, showing the thermal stability of the COFs up to 350 °C, under nitrogen atmosphere.

S4.6 Solid-state NMR

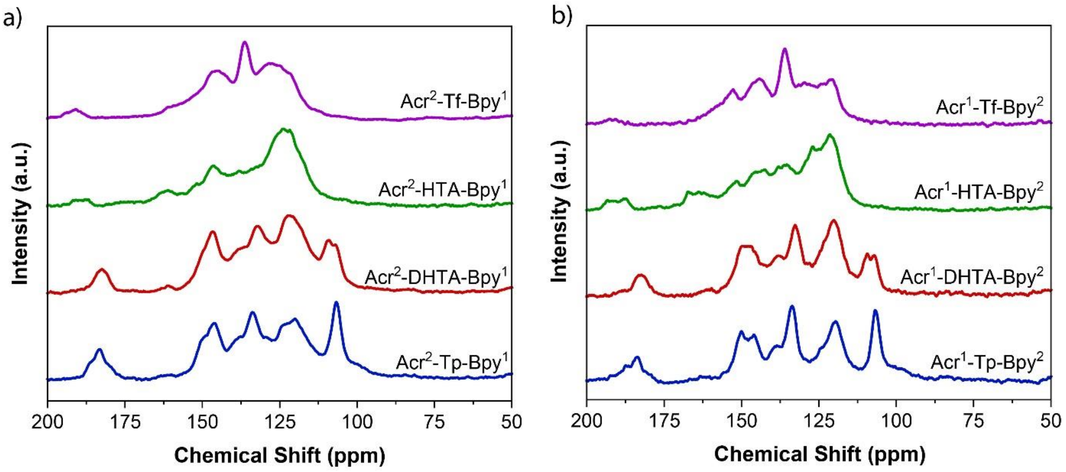

Figure S 11. ^{13}C CP-MAS NMR spectra of a) Acr^{2}-L-Bpy^{1} and b) Acr^{1}-L-Bpy^{2} COFs.

S4.7 X-ray photoelectron spectroscopy (XPS) analysis of Acr^x-L-Bpy^y COFs

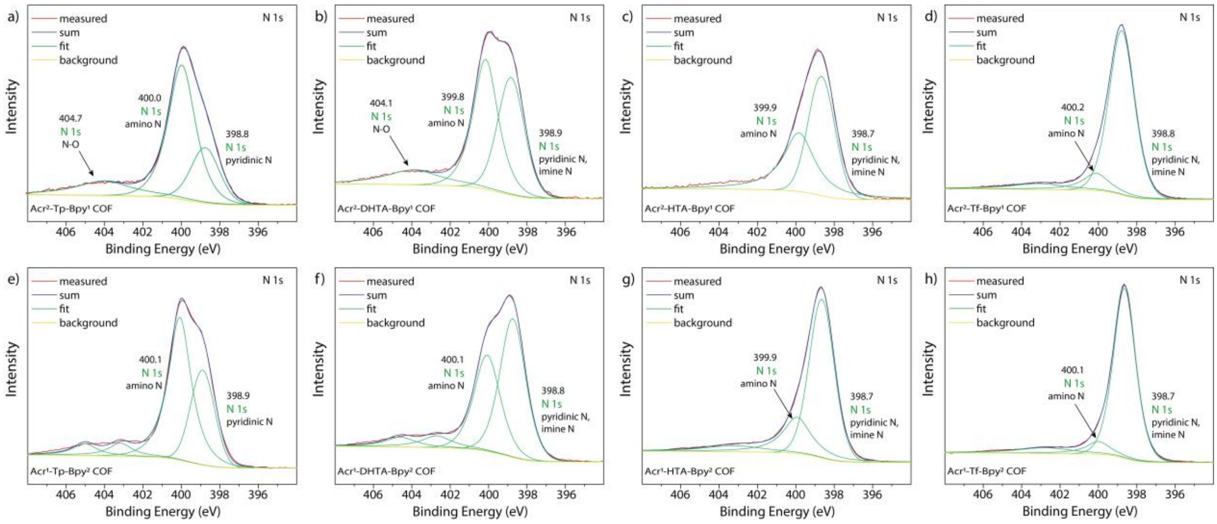

Figure S 12. (a, b, c, d) N 1s XPS core-level spectra of the Acr^2-L-Bpy^1 multi-component COFs [L= Tp (a), DHTA (b), HTA (c), Tf (d)]. (e, f, g, h) N 1s XPS core-level spectra of the Acr^1-L-Bpy^2 COFs [L= Tp (e), DHTA (f), HTA (g), Tf (h)].

S4.8 COF digestion of Acr^x-Tp-Bpy^y

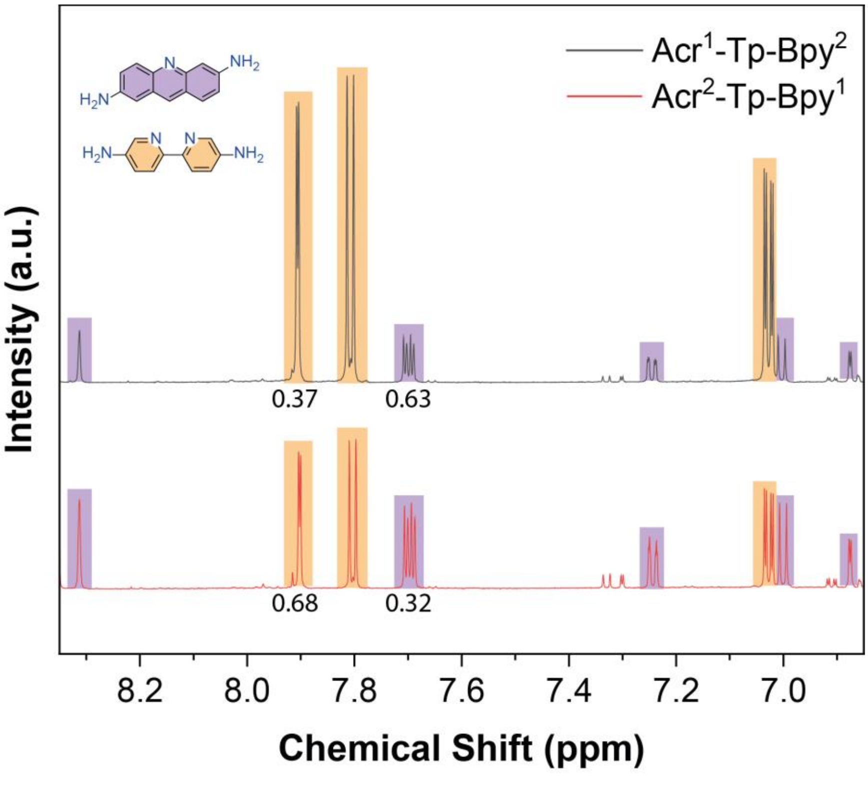

Figure S 13. Partial ^{1}H NMR spectra (DMSO-d$_6$, 700 MHz, 298K) of digested Acr^x-Tp-Bpy^y COFs. Each material sample was were digested in a solution of 0.1 mL of 10 M NaOH in D_2O and 0.5 mL DMSO-d$_6$ at 120 °C for 3 h.

S4.9 UV-vis of Acr^x-L-Bpy^x COFs

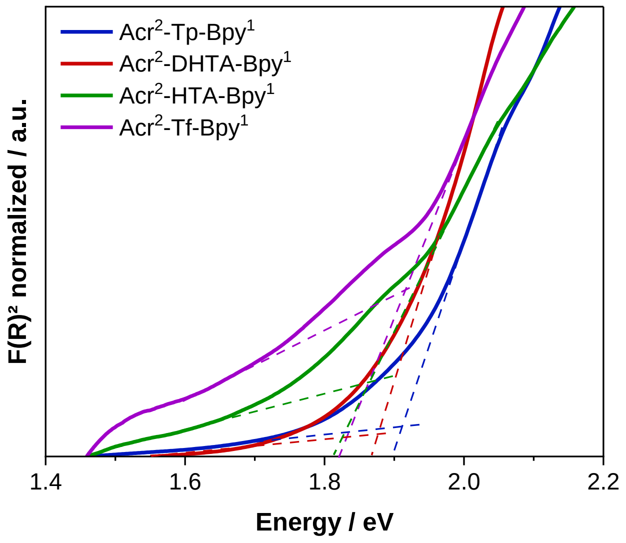

Figure S 14. Tauc plots for Acr^2-L-Bpy^1 COFs.

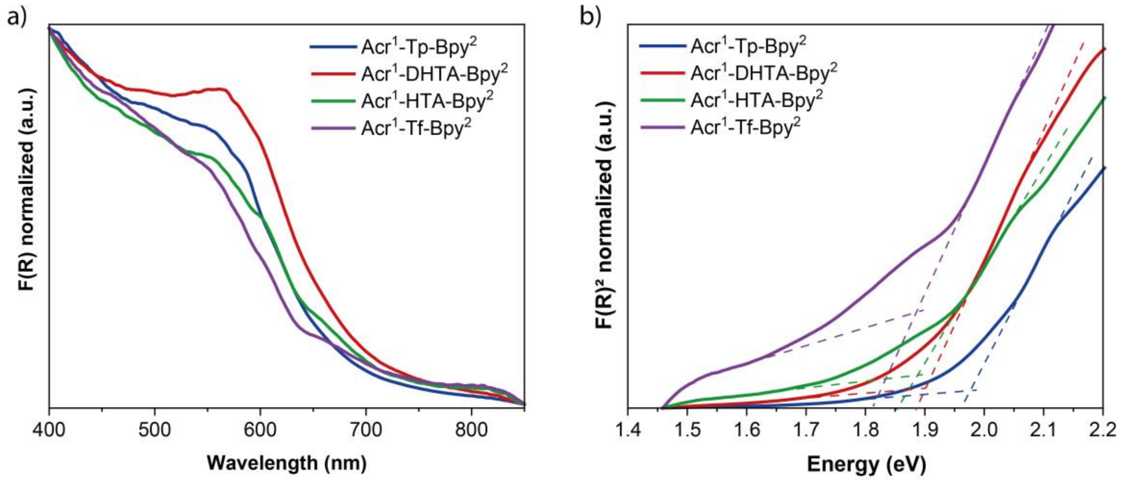

Figure S 15. a) UV-vis diffuse reflectance spectroscopy analysis of Acr^1-L-Bpy^2 COFs. b) Tauc plots for Acr^1-L-Bpy^2 COFs.

S4.10 Comparison of theoretical and experimental excitation energies and band gaps

Density functional theory using gradient corrected or hybrid functionals with dispersion corrections were used to compare experimental and theoretical excitation energies and band gaps (see "Computational details" below), as well as the stability of keto and enol forms, and effects of metalation. Both periodic calculations and cluster models were used, the former giving band gaps, the latter, via linear response time-dependent DFT (TD-DFT) in addition excitation energies and oscillator strengths.

Periodic calculations were performed on AA-stacked COF periodic models for L-Acr, Acr^2-L-Bpy^1 and Acr^2-L-Bpy^1-Ni for single-layer models (L = Tp, DHTA, HTA, Tf). Relative energies of keto and enol tautomers at the PBE+D3 level show a strong preference (around 0.4 to 1.1 eV per unit cell) for keto-Tp and keto-DHTA, while enol HTA COFs are slightly more stable (by less than 0.1 eV) than keto HTA COFs. Band gaps between keto and enol tautomers typically differ by up to 0.2 eV at the PBE level (see Table S1, below), indicating a strong influence of keto-enol tautomerism on the electronic structure. Cluster models consisting of one complete pore were built from the PBE+D3 optimized planar bulk structures and reoptimized using PBE+D3. The results reported here all use a cluster model with amine groups capping the structure to ensure fully hydrogen-bonded keto groups if applicable (Figure S16).

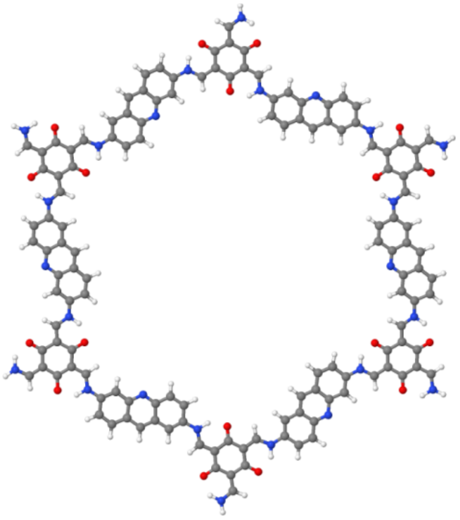

Figure S 16. Cluster models of Tp-Acr. The model is cut to keep the hydrogen-bonded amine groups.

Table S 1. Indirect band gaps in eV for planar periodic models; HOMO-LUMO gaps, E_g, and excitation energies (1st "bright" transition), E_{ex}, in eV for cluster models (in parentheses: enol tautomers).

		Periodic models		Cluster models		
	experimental UV-vis DRS	PBE planar, one layer	HSE PBE opt. one layer	PBE E_g	CAM-B3LYP E_g	CAM-B3LYP E_{ex}
Tp-Acr	1.83 [a]	1.38	2.07	1.48	4.82	3.03
DHTA- Acr	1.83 [a]	1.19	1.84	1.07	4.31	2.71
HTA-Acr	1.82 [a]	0.94	1.54	0.82 (1.35)	4.08 (4.80)	2.59 (3.16)
Tf-Acr	1.87	1.66	2.35	1.68	5.04	3.23
Acr^2-Tp-Bpy^1	1.92	1.41	2.11	1.53	4.89	3.03
Acr^2-DHTA-Bpy^1	1.87	1.30	1.96	1.21	4.48	2.75
Acr^2-HTA-Bpy^1	1.84	0.98	1.59	0.83 (1.33)	4.09 (4.75)	2.64 (3.22)
Acr^2-Tf-Bpy^1	1.90	1.66	2.36	1.68	5.04	3.23
Acr^2-Tp-Bpy^1-Ni		1.01	1.94	1.09	4.63	2.92
Acr^2-DHTA-Bpy^1-Ni		1.07	1.65	0.73	3.97	2.69
Acr^2-HTA-Bpy^1-Ni		0.77	1.43	0.66 (0.81)	3.85 (4.37)	2.58 (2.95)
Acr^2-Tf-Bpy^1-Ni	1.92 [b]	0.88	2.06	0.85	4.59	3.15

[a] Values obtained from literature.[6] [b] Degree of metalation 19.4%

In Table S1 we compare indirect band gaps calculated from periodic models and HOMO-LUMO gaps and lowest bright transition energies for L-Acr, Acr^2-L-Bpy^1 and Acr^2-L-Bpy^1-Ni to the onset of measured UV-vis DRS spectra. Values for L-Acr have been obtained from literature.[6] The exchange of an acridine unit with a bipyridine moiety is not having a significant impact on the optical properties, which can be confirmed with the theoretical calculations. Metalation of this bipyridine unit is in experimental spectra not resulting in a change of the band gap, while calculations would suggest a red shift. This is most likely because of incomplete metalation of the COF. Calculations suggest that the removal of additional hydroxy groups from Tp to DHTA and HTA results in a gradual decrease in band gap, while Tf linked COFs are not following this trend and have again larger band gaps. However, this trend is not observed for acridine containing COFs, while purely bipyridine linked COFs are following this trend (Figure S24). This suggests an insufficient representation of the acridine chromophore in the calculations. The absolute values for all calculated band gaps on the PBE level of theory differ significantly from the ones derived from UV-vis measurements. This is not unexpected, since PBE gives smaller gaps and excitation energies compared to functionals containing exact exchange (HSE and CAM-B3LYP). Further, in the cluster models only a single hexagonal pore was used for the calculations, and no defects/imperfections were considered. In fact, periodic models (on the PBE level of theory) provide a smaller gap when compared to cluster models. In passing we note that calculations indicate buckled/twisted structures being more stable than plana ones, which may also have an effect on the optoelectronic properties of the materials.

Computational details: Calculations on periodic models were performed with VASP[7,8], version 5.4.4. Atomic positions and cell parameters were optimized using the PBE functional[9] with the D3 dispersion correction[10] with Becke-Johnson damping.[11] Projector augmented-wave[12,13] pseudopotentials were used, which included the semi-core s and p states for Ni (resulting in 18 explicitly treated electrons). The kinetic energy cutoff was set to 600 eV and a 2×2×4 k point grid was used. SCF and force convergence criteria were set to 10^{-6} eV and 10^{-2} eV/Å, respectively. Single-layer models employ a cell vector perpendicular to the layer of 20 Å, resulting in a vacuum space of at least 16 Å for the twisted COFs. HSE[14,15] single-point calculations were performed on the PBE-optimized single-layer models.

Cluster calculations were performed using ORCA,[16] version 4.0.0.2. The cluster models were based on the periodic models and reoptimized using the PBE functional[9] with the D3 dispersion correction[10] with Becke-Johnson damping.[11] A def2-TZVP basis set[17] was employed together with an auxiliary def2 basis set for the RI approximation. CAM-B3LYP[18–21] calculations were performed using the def2-SVP basis set.[17] Time-dependent density functional theory (TD-DFT)[22] was used to calculate excitation energies and oscillator strengths.

S4.11 Photoluminescence measurements of Acr²-L-Bpy¹ multicomponent COFs

Preparation of the samples for luminescence studies: 0.5 mg of COF was dispersed in 10 ml acetonitrile by sonicating for 5 minutes at 40 °C. Prior the UV-vis and PL measurements the bigger particles were let to sediment to obtain a nearly clear solution. The clear COF dispersed solutions were used for the PL measurements.

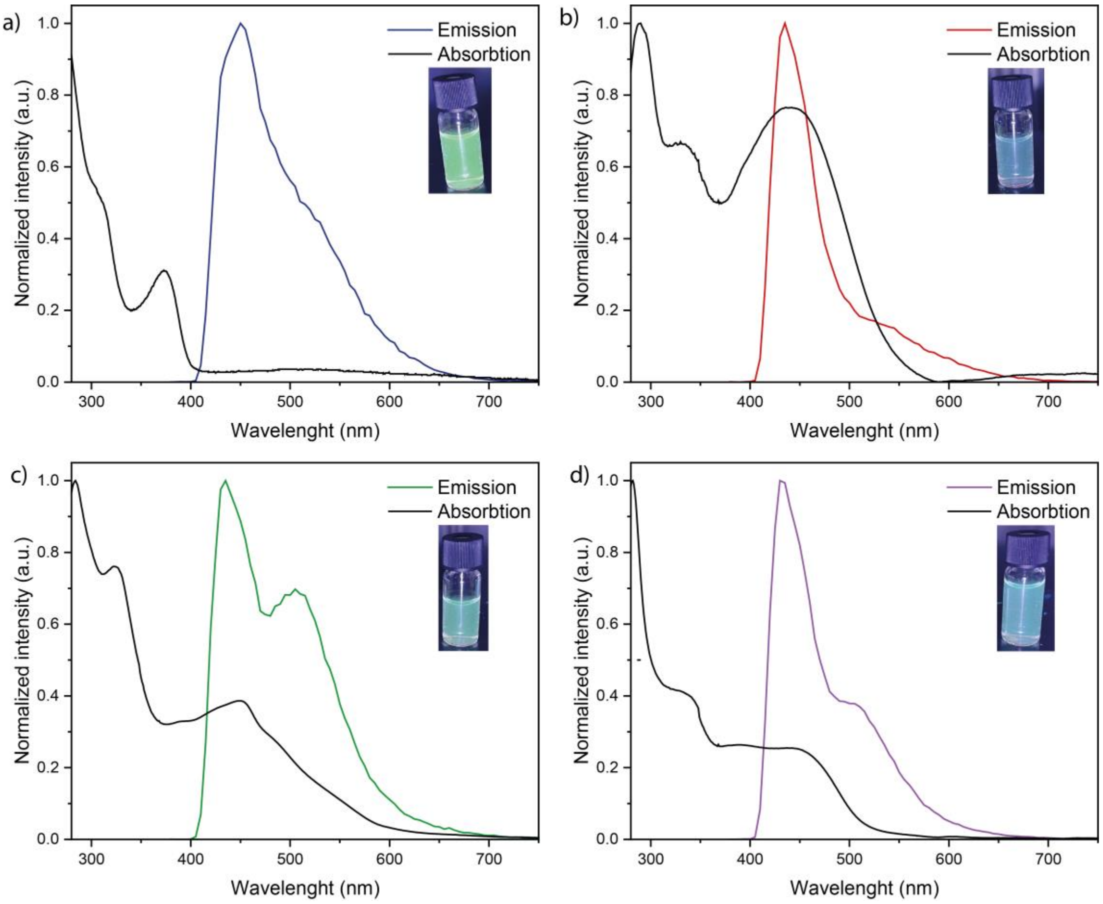

Figure S 17.Steady-state absorption and emission profiles of dispersed a) Acr²-Tp-Bpy¹, b) Acr²-DHTA-Bpy¹, c) Acr²-HTA-Bpy¹ and d) HTA-Acr in acetonitrile. For the fluorescence measurements the samples were excited at a wavelength of 360 nm. Optical images at ambient light and upon excitation under a 365 nm UV-lamp is shown in every case.

S4.12 Fluorescence quenching studies of Acr[2]-Tf-Bpy[1] multicomponent COF

Preparation of the samples for luminescence studies:

0.5 mg of COF was dispersed in 10 ml DMSO by sonicating for 10 minutes. Prior the fluorescence measurements the bigger particles were let to sediment to obtain a nearly clear solution.

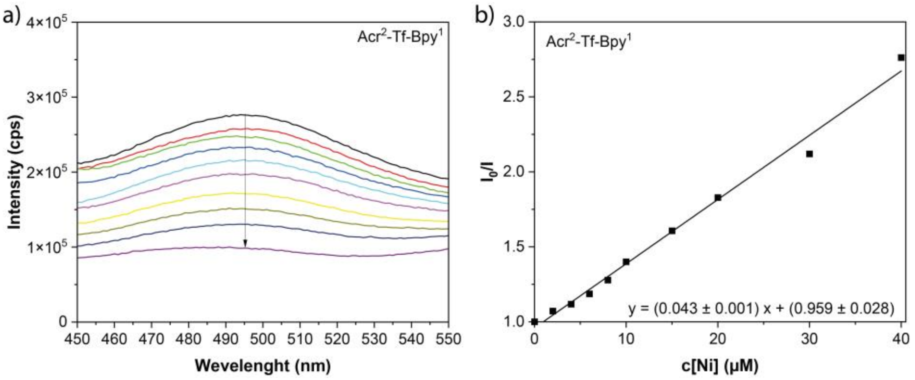

Figure S 18. a) Fluorescence quenching experiments of Acr[2]-Tf-Bpy[1] COF in presence of $NiCl_2$·glyme. b) Stern-Volmer quenching plot.

S4.13 Electron paramagnetic resonance (EPR) spin trap experiments of Acr[2]-Tf-[Ni][1] and Acr[2]-Tp-[Ni][1] multicomponent COFs

Acr[2]-Tf-[Ni][1] and Acr[2]-Tp-[Ni][1] COFs were prepared by ex-situ metalation of the respective multicomponent frameworks in acetonitrile with an excess of $NiCl_2$·glyme at 40 °C for 3 h while stirring the suspension. The material was filtered and washed with H2O dist., MeOH and cyclohexane before drying under vacuum. Electron paramagnetic resonance (EPR) spectra were recorded on a X-band Bruker EMX CW-micro EPR spectrometer equipped with an ER4119HS high-sensitivity resonator using modulation frequency of 100 kHz and modulation amplitude up to 1G. The experimental spectrum was simulated with EasySpin program. In-situ EPR spectrum was recorded at 20 °C during irradiating a home-made EPR quartz flat-cell (ID 0.5 mm) filled under Ar with a suspension of COF catalysts (5 mg COF + 20µL 4-iodobenzotrifluoride + 5,5-dimethyl-1-pyrrolin-N-oxid (DMPO) solution prepared by dissolving of 10 µl DMPO in 0.5 MeCN). In situ EPR spectra were recorded before and after irradiation the sample with a 300 W Xe-arc lamp (LOT Oriel GmbH, Germany) using a cut-off filter GG420.

To gain more knowledge about the reason for different catalytic activity behaviors, in situ EPR investigations were conducted in the presence of DMPO as spin trap reagent to detect the

possible formation of organic radical during the catalytic reaction. Photoirradiation of Acr^2-Tf-[Ni]^1 in the presence of 4-iodobenzotrifluoride caused appearing of multiple-lines EPR signal at g = 2.007 and its intensity increased with irradiation time (Figure S18a). This signal can be simulated assuming the coupling of electron spin half center (S = 1/2) interacting with two nonequivalent nitrogen nuclei (I = 1; A_{N1} = 13.23 G and A_{N2} = 1.27 G) and one hydrogen nucleus (I = 1/2; A_H = 8.94 G) suggesting the formation of DMPO-N spin adduct (Figure S18b, red line). This indicates the formation of N centered radical during the irradiation of the Acr^2-Tf-[Ni]^1 catalyst. The EPR spectrum also showed the appearing of an additional week signal with time. The origin of this signal is not clear since it is very weak and only its outer lines were resolved (indicated by a star in Figure S18b, black line). In comparison to the Acr^2-Tf-[Ni]^1 COF, very weak signal was observed for the Acr^2-Tp-[Ni]^1 (Figure S19, blue line) revealing the low photocatalytic activity of the keto-enamine linked COF compared to the fully imine multivalent framework.

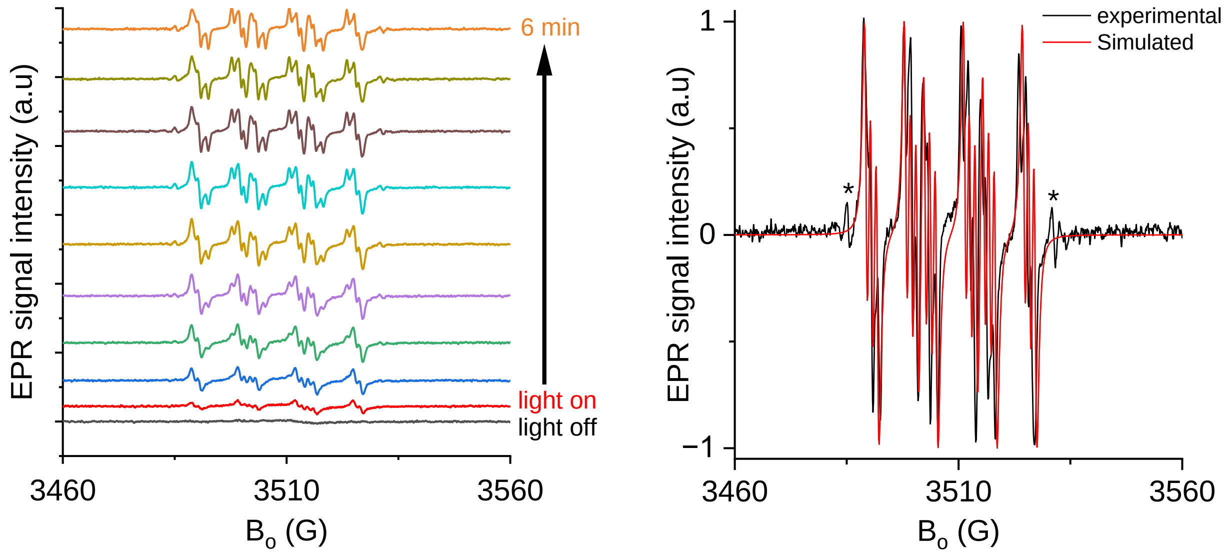

Figure S 19. a) EPR spectra of Acr^2-Tf-[Ni]^1 in 0.5 CH_3CN in the presence of 4-iodobenzotrifluoride and DMPO during irradiation, measured with modulation amplitude of 0.3 G for better resolving the small hyperfine structures. b) simulation of the experimental spectrum after 4 min irradiation.

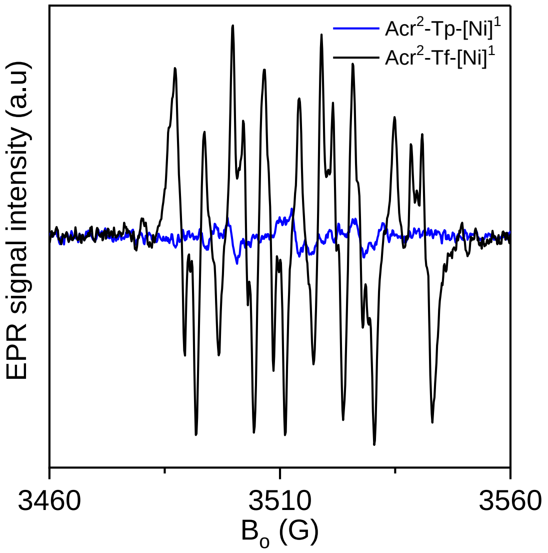

Figure S 20. EPR spectra of Acr[2]-Tf-[Ni][1] (black line) and Acr[2]-Tp-[Ni][1] in CH_3CN in the presence of 4-iodobenzotrifluoride and DMPO during irradiation for 1 min), measured with modulation amplitude of 1 G for better signal intensity.

S5. Synthesis and characterization of reference COFs

S5.1 Synthesis of bipyridine COFs

Tp-Bpy COF: A Pyrex tube (o.d. × i.d. = 15 × 10 mm^2 and length 15 cm) is charged with triformylphloroglucinol (**Tp**) (21 mg, 0.1 mmol), 2,2′-bipyridine-5,5′-diamine (**Bpy**) (27.9 mg, 0.15 mmol), 1.5 mL of mesitylene, 1.5 mL of 1,4-dioxane and 0.5 mL of 6 M aqueous acetic acid. This mixture was sonicated for 15 minutes in order to get a homogenous dispersion. The tube was then flash frozen at 77 K (liquid N_2 bath) and degassed by three freeze-pump-thaw cycles. The tube was sealed off and then heated at 120 °C for 3 days. An orange colored precipitate was collected by filtration and washed with acetone, methanol and cyclohexane. The powder collected was dried at 120 °C to give a dark orange colored powder (43 mg, 99 %).

DHTA-Bpy COF: A Pyrex tube (o.d. × i.d. = 15 × 10 mm^2 and length 15 cm) is charged with 2,4-dihydroxybenzene-1,3,5-tricarbaldehyde (**DHTA**) (19.4 mg, 0.1 mmol), 2,2′-bipyridine-5,5′-diamine (**Bpy**) (27.9 mg, 0.15 mmol), 1.5 mL of mesitylene, 1.5 mL of 1,4-dioxane and 0.5 mL of 6 M aqueous acetic acid. This mixture was sonicated for 15 minutes in order to get a homogenous dispersion. The tube was then flash frozen at 77 K (liquid N_2 bath) and degassed by three freeze-pump-thaw cycles. The tube was sealed off and then heated at 120 °C for 3 days. A red colored precipitate was collected by filtration and washed with acetone, methanol and cyclohexane. The powder collected was dried at 120 °C to give a bright red colored powder (34 mg, 81 %).

HTA-Bpy COF: A Pyrex tube (o.d. × i.d. = 15 × 10 mm^2 and length 15 cm) is charged with 2-hydroxybenzene-1,3,5-tricarbaldehyde (**HTA**) (17.8 mg, 0.1 mmol), 2,2′-bipyridine-5,5′-diamine (**Bpy**) (27.9 mg, 0.15 mmol), 1.5 mL of mesitylene, 1.5 mL of 1,4-dioxane and 0.5 mL of 6 M aqueous acetic acid. This mixture was sonicated for 15 minutes in order to get a homogenous dispersion. The tube was then flash frozen at 77 K (liquid N_2 bath) and degassed by three freeze-pump-thaw cycles. The tube was sealed off and then heated at 120 °C for 3 days. A red colored precipitate was collected by filtration and washed with acetone, methanol and cyclohexane. The powder collected was dried at 120 °C to give a bright red colored powder (40 mg, 99 %).

Tf-Bpy COF: A Pyrex tube (o.d. × i.d. = 15 × 10 mm^2 and length 15 cm) is charged with 1,3,5-triformylbenzene (**Tf**) (16.2 mg, 0.1 mmol), 2,2′-bipyridine-5,5′-diamine (**Bpy**) (27.9 mg, 0.15 mmol), 1.5 mL of mesitylene, 1.5 mL of 1,4-dioxane and 0.5 mL of 6 M aqueous acetic acid. This mixture was sonicated for 15 minutes in order to get a homogenous dispersion. The tube was then flash frozen at 77 K (liquid N_2 bath) and degassed by three freeze-pump-thaw cycles. The tube was sealed off and then heated at 120 °C for 3 days. A yellow colored precipitate was collected by filtration and washed with acetone, methanol and cyclohexane. The powder collected was dried at 120 °C to give a bright yellow colored powder (32 mg, 82 %).

S5.2 Characterization of bipyridine COFs

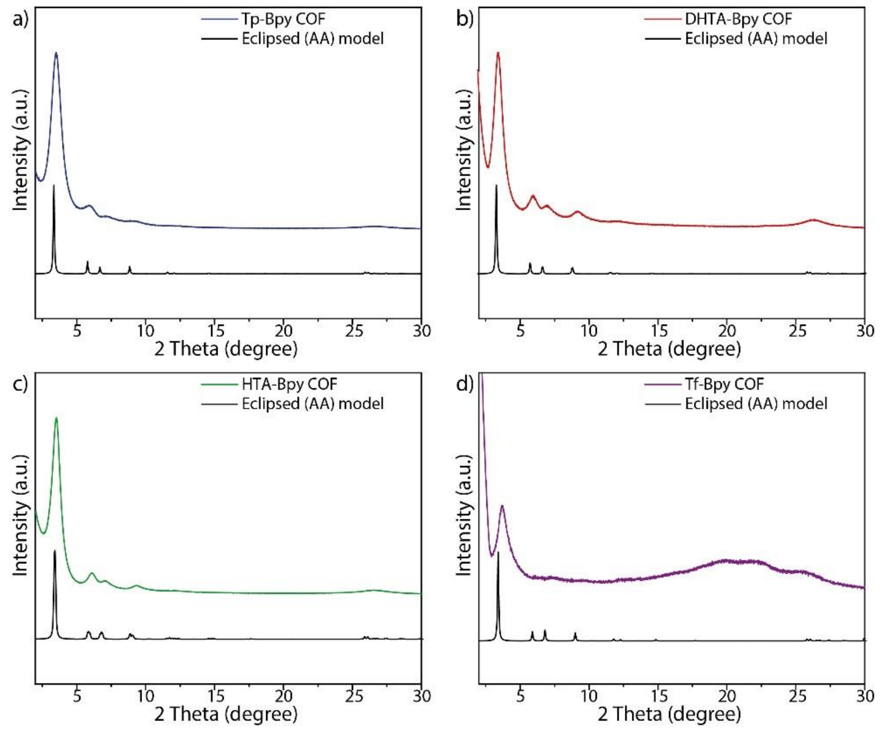

Figure S 21. Comparison between simulated and experimental PXRD patterns for a) Tp-Bpy, b) DHTA-Bpy, c) HTA-Bpy and d) Tf-Bpy COFs, showing agreement between the experimental diffractogram and the eclipsed stacking model (AA).

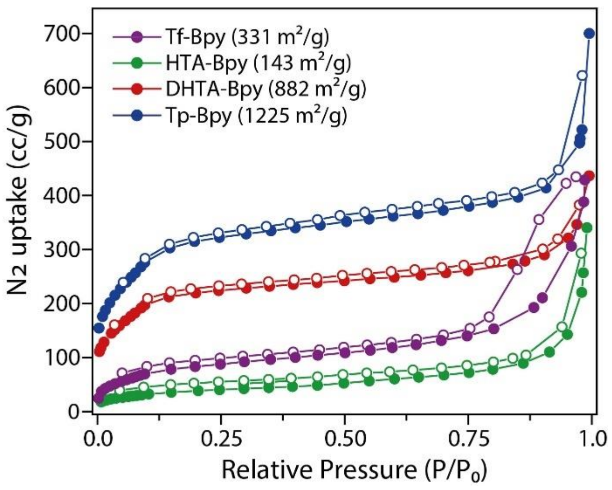

Figure S 22. N_2 sorption isotherms for Tp-Bpy, DHTA-Bpy, HTA-Bpy and Tf-Bpy COFs, calculated BET surface areas are shown in brackets.

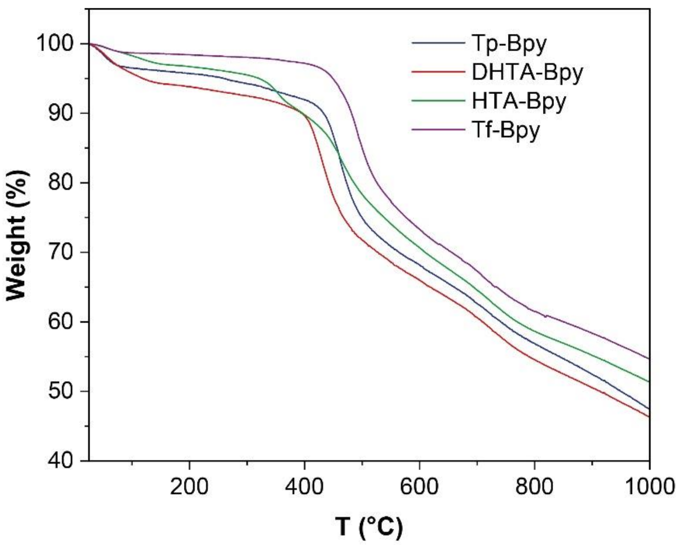

Figure S 23. Thermogravimetric analyses (TGA) for Tp-Bpy, DHTA-Bpy, HTA-Bpy and Tf-Bpy COFs, showing the thermal stability of the COFs below 350 °C, under nitrogen atmosphere.

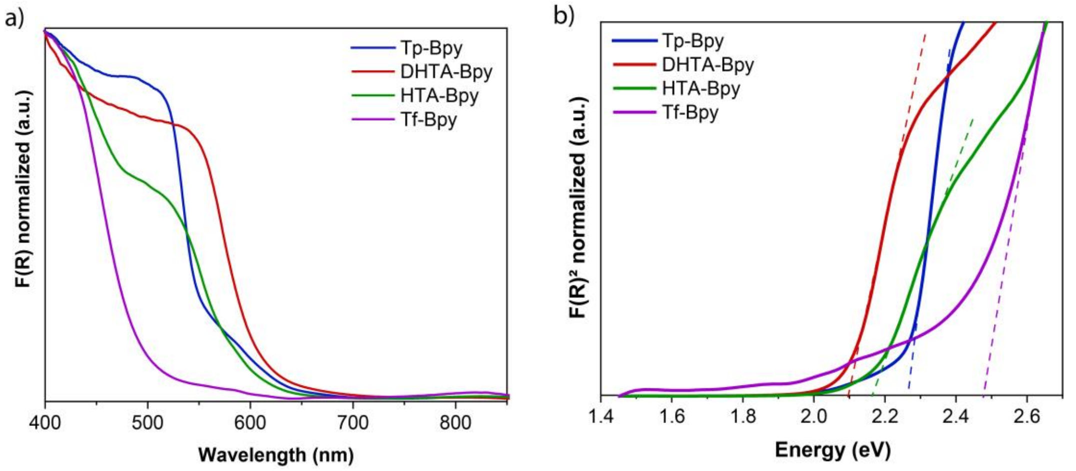

Figure S 24. a) UV-vis diffuse reflectance spectroscopy analysis of Acr¹-L-Bpy² COFs. b) Tauc plots for Acr¹-L-Bpy² COFs.

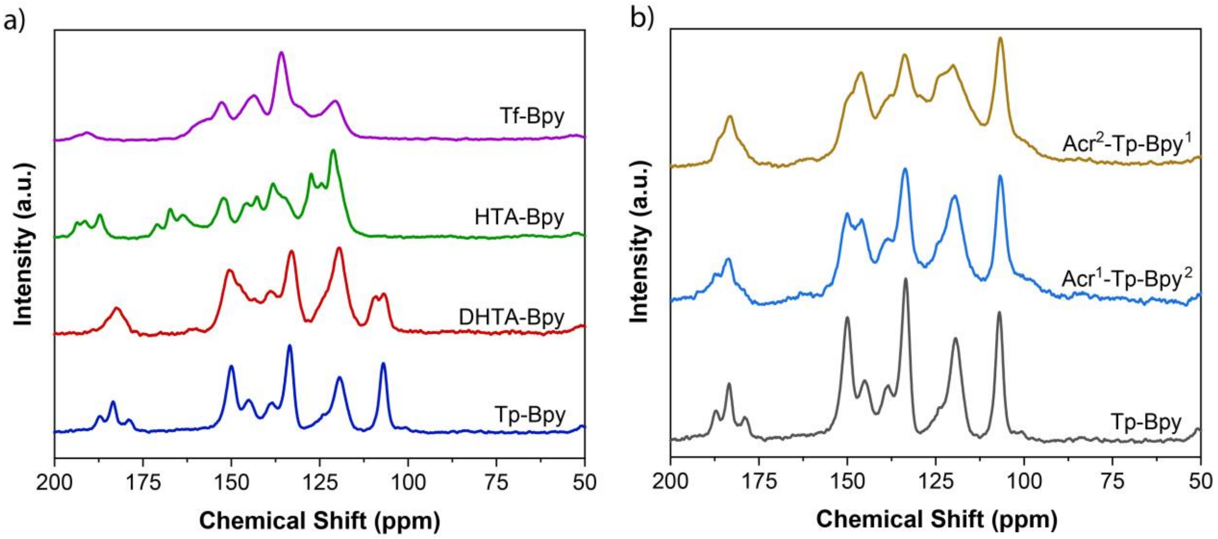

Figure S 25. a) ^{13}C CP-MAS NMR spectra of Tp-Bpy, DHTA-Bpy, HTA-Bpy and Tf-Bpy COF. b) Following the trend of introduction of bipyridine units for Acr^{x}-Tp-Bpy^{y} COFs.

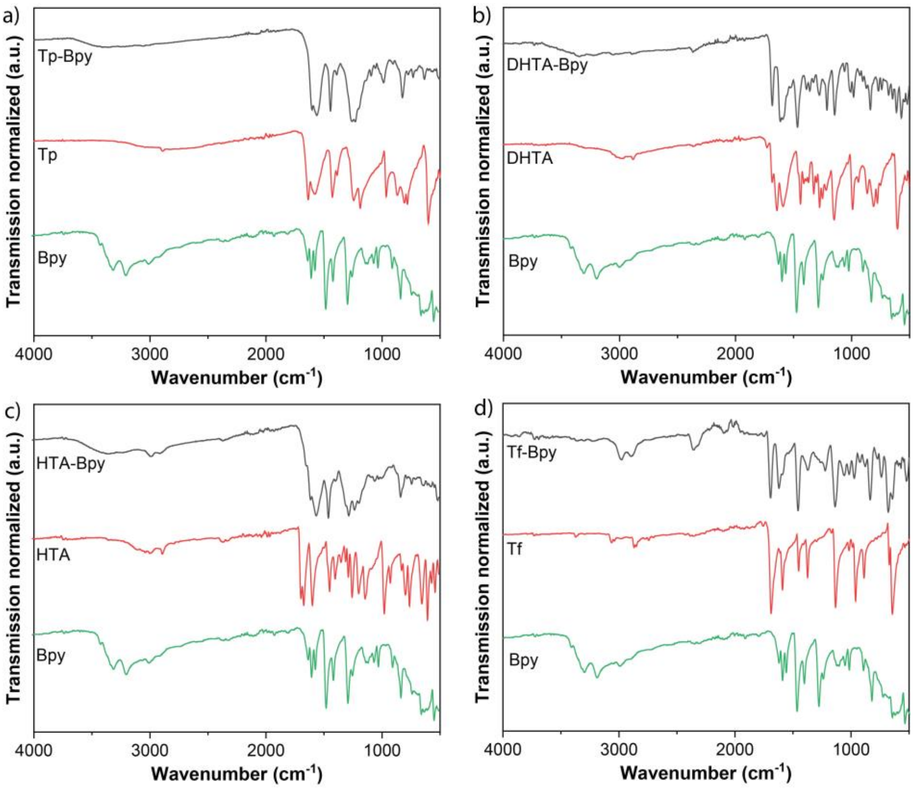

Figure S 26. FT-IR analyses of L-Bpy COFs [L = Tp (a), DHTA (b), HTA (c), Tf (d)] in comparison with the corresponding aldehydes and amine (Bpy) showing the formation of the framework structure.

S5.3 Synthesis of Tf-Acr COF

Tf-Acr COF: A Pyrex tube (o.d. × i.d. = 15 × 10 mm^2 and length 15 cm) is charged with 1,3,5-triformylbenzene (**Tf**) (16.2 mg, 0.1 mmol), 2,6-diaminoacridine (**Acr**) (31,5 mg, 0.15 mmol), 1.5 mL of *n*-BuOH, 1.5 mL of anhydrous *o*-DCB and 0.5 mL of 6 M aqueous acetic acid. This mixture was sonicated for 15 minutes in order to get a homogenous dispersion. The tube was then flash frozen at 77 K (liquid N_2 bath) and degassed by three freeze-pump-thaw cycles. The tube was sealed off and then heated at 120 °C for 3 days. A dark red precipitate was collected by filtration and washed with acetone, methanol and cyclohexane. The powder collected was dried at 120 °C to give a dark red powder (31 mg, 72 %).

S5.4 Characterization of Tf-Acr COF

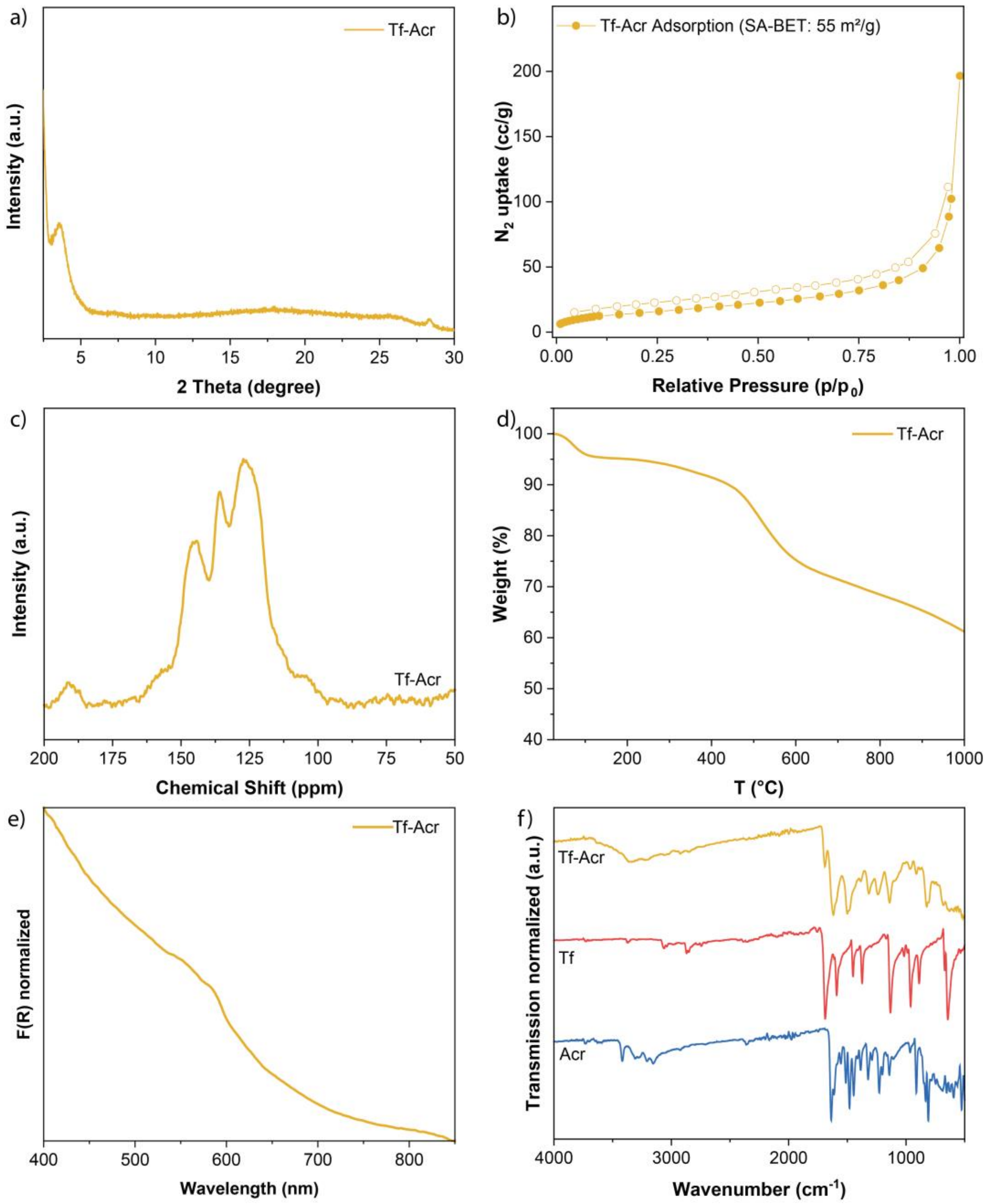

Figure S 27. Characterization of Tf-Acr COF using PXRD (a), N_2 sorption (b) ^{13}C CP-MAS NMR (c), TGA (d), diffuse reflectance UV-vis (e) and FT-IR (f).

S6. Synthesis and characterization of model compounds

S6.1 Synthesis of acridine model compound (SA-Acr)

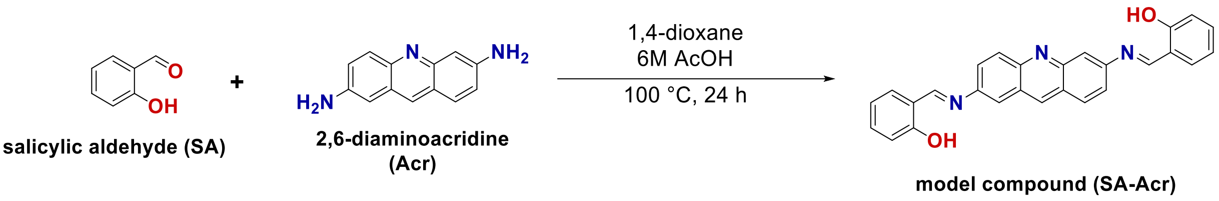

Scheme S 7. Synthesis of the model compound SA-Acr.

Model compound (SA-Acr): To a Schlenk flask were 63 mg (0.30 mmol, 1.0 eq.) of 2,6-diaminoacridine (Acr), 68 µl (0.66 mmol, 2.2 eq.) and 5 mL of 1,4-dioxane added. To the resulting suspension 1.0 mL of 6 M aqueous acetic acid were added. The flask was closed and heated to 100 °C for 24 h while stirring the reaction mixture. After cooling to room temperature, the resulting yellow-brown suspension was filtered and washed with 10 mL acetone to remove residual starting materials. The model compound was subsequently dried under vacuo to give the desired compound as a dark brown powder (71 mg, 57%).

ESI-MS [M+H$^+$] (m/z)): 418.1549 (th.: 418.1550). Due to the low solubility of this compound in common deuterated solvents, no ^{13}C NMR spectra could be recorded.

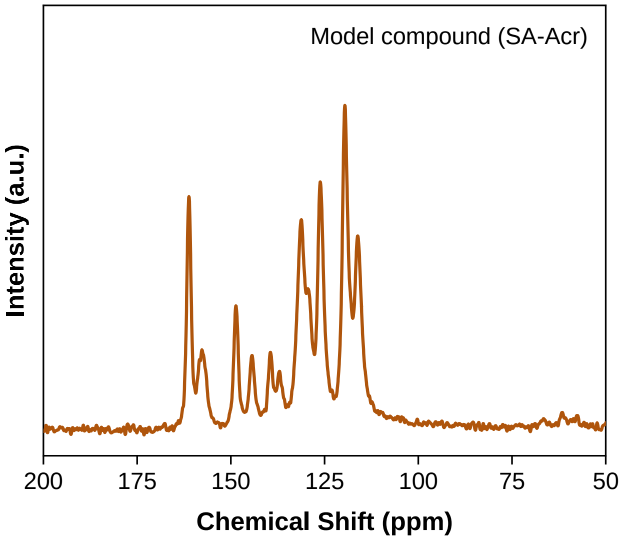

Figure S 28. ^{13}C CP-MAS solid-state NMR spectra of the model compound SA-Acr.

S6.2 Synthesis of bipyridine model compounds (BA-Bpy)

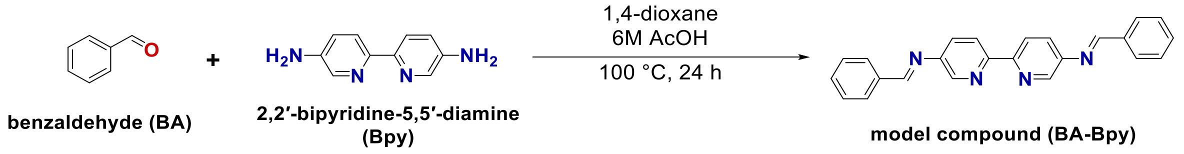

Scheme S 8. Synthesis of the model compound BA-Bpy.

Model compound (BA-Bpy): To a Schlenk flask were 56 mg (0.30 mmol, 1.0 eq.) of 2,2'-bipyridine-5,5'-diamine (Bpy), 67 µl (0.66 mmol, 2.2 eq.) of benzaldehyde and 5 mL of 1,4-dioxane added. To the resulting solution 1.0 mL of 6 M aqueous acetic acid were added. The flask was closed and heated to 100 °C for 24 h while stirring the reaction mixture. After cooling to room temperature, the resulting dark yellow suspension was filtered and washed with 10 mL of diethyl ether to remove residual starting materials. The model compound was subsequently dried under vacuo to give the desired compound as a dark yellow powder (75 mg, 69%).

^{1}H NMR (700 MHz, DMSO) δ 8.81 (s, 2H), 8.63 (d, J = 2.5 Hz, 2H), 8.45 (d, J = 8.4 Hz, 2H), 8.00 (dd, J = 7.9, 1.3 Hz, 4H), 7.89 (dd, J = 8.4, 2.5 Hz, 2H), 7.61 – 7.54 (m, 6H) ppm. ^{13}C NMR (176 MHz, DMSO) δ 162.9, 152.6, 147.2, 143.0, 135.8, 132.0, 129.0, 128.9, 128.7, 120.7 ppm. ESI-MS [M+H^{+}] (m/z)): 363.1603 (th.: 363.1604).

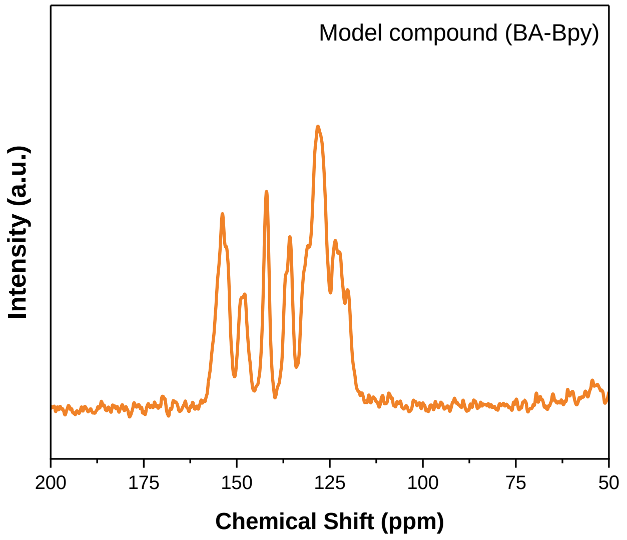

Figure S 29. ^{13}C CP-MAS solid-state NMR spectra of the model compound BA-Bpy. The peaks found are in full agreement with the liquid state NMR results.

S7. Photocatalytic reaction optimization

S7.1 General experimental procedure for screening experiments

An oven dried vial (19 x 100 mm) equipped with a stir bar was charged with $NiCl_2$·glyme (4-12 µmol), 3 mg of Acr^x-L-Bpy^y COF, 4-halobenzotrifluoride (100 µmol) and nucleophile (200 µmol). The solvent (anhydrous) was added and the vessel was sealed with a septum and Parafilm. The mixture was stirred for 1 minute at high speed, followed by sonication for 5 minutes and degassing by bubbling argon for 10 minutes. The reaction mixture was stirred at 800 rpm and irradiated with 440 nm, 535 nm or 666 nm LED lamps using the reported power settings. After the respective reaction time, 1,3,5-trimethoxybenzene (1 equiv) was added to the reaction vessel, the mixture was shaken and an aliquot (200 µL) was removed, filtered, diluted with DMSO-d_6 and analyzed by ^{1}H NMR.

S7.2 Initial screening experiments using sodium *p*-toluensulfinate

Table S 2. Screening experiments for the coupling of 4-iodobenzotrifluoride and sodium *p*-toluensulfinate using 440 nm.[a]

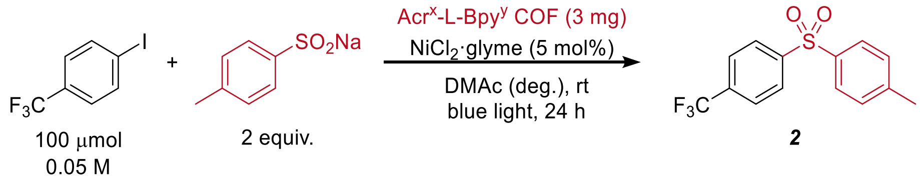

Entry	Variations	2 [%][c]
MT30	Tp-Bpy	6
MT31	DHTA-Bpy	16
MT32	HTA-Bpy	28
MT33	Acr^2-Tp-Bpy^1	15
MT34	Acr^2-DHTA-Bpy^1	20
MT35	Acr^2-HTA-Bpy^1	34
MT36	No COF	traces

[a]Reaction conditions: 4-iodobenzotrifluoride (100 µmol), sodium *p*-toluensulfinate (200 µmol), $NiCl_2$·glyme (5 µmol), COF (3 mg), DMAc (anhydrous, 2 mL), 440 nm LED (1 lamp at full power). [b]Conversion of 4-iodobenzotrifluoride determined by ^{1}H-NMR using 1,3,5-trimethoxybenzene as internal standard. [c]NMR yields determined by ^{1}H-NMR using 1,3,5-trimethoxybenzene as internal standard. glyme = 1,2-dimethoxyethane. n.d. = not detected.

Table S 3. Screening experiments for the coupling of 4-iodobenzotrifluoride and sodium *p*-toluensulfinate using 525 nm.[a]

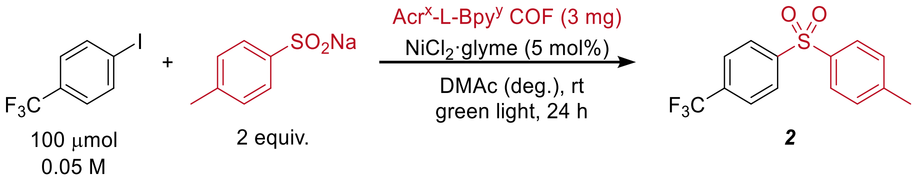

Entry	Variations	2 [%][c]
MT30	Tp-Bpy	3
MT31	DHTA-Bpy	5
MT32	HTA-Bpy	17
MT33	Acr^2-Tp-Bpy^1	9
MT34	Acr^2-DHTA-Bpy^1	7
MT35	Acr^2-HTA-Bpy^1	11
MT36	No COF	traces

[a]Reaction conditions: 4-iodobenzotrifluoride (100 µmol), sodium *p*-toluensulfinate (200 µmol), NiCl$_2$·glyme (5 µmol), COF (3 mg), DMAc (anhydrous, 2 mL), 525 nm LED (1 lamp at full power). [b]Conversion of 4-iodobenzotrifluoride determined by ^{1}H-NMR using 1,3,5-trimethoxybenzene as internal standard. [c]NMR yields determined by ^{1}H-NMR using 1,3,5-trimethoxybenzene as internal standard. glyme = 1,2-dimethoxyethane. n.d. = not detected.

S7.3 Screening experiments for different COFs

Table S 4. Screening experiments for the coupling of 4-iodobenzotrifluoride and sodium *p*-toluensulfinate using different COFs.

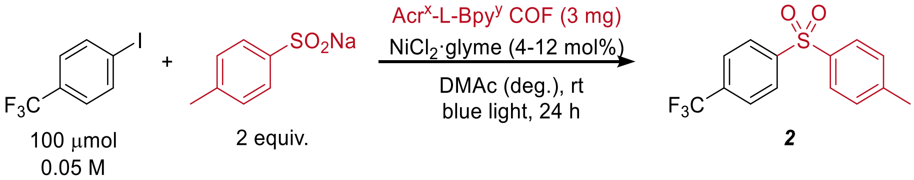

Acr:Bpy (Ni mol%)	Tp	DHTA	HTA	Tf
2:1 (4 mol%)	25	33	43	55
1:2 (8 mol%)	19	29	35	49
0:1 (12 mol%)	10	30	39	59

increasing activity →

← increasing number of hydroxy groups

Reaction conditions: 4-iodobenzotrifluoride (100 µmol), sodium *p*-toluenesulfinate (200 µmol), NiCl$_2$·glyme (4-12 mol%), COF (3 mg), DMAc (anhydrous, 2 mL), 440 nm LED (2 lamps at full power). NMR yields determined by ^{1}H-NMR using 1,3,5-trimethoxybenzene as internal standard.

S7.4 Screening different COFs for cross-coupling of aryl halides and nucleophiles

Table S 5. Screening experiments for the coupling of 4-iodobenzotrifluoride and methyl 3-mercaptopropanoate using different COFs.

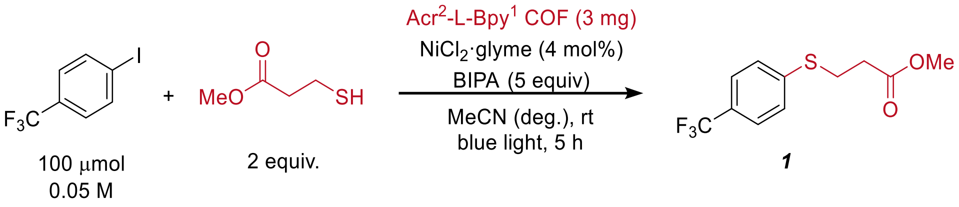

Acr:Bpy (Ni mol%)	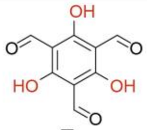 Tp	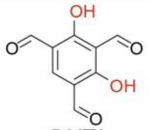 DHTA	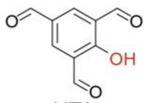 HTA	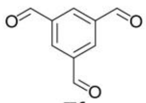 Tf
2:1, 2 h	4 (21)	41 (46)	59 (64)	86 (86)
2:1, 5 h	54 (54)	99 (quant.)	92 (quant.)	96 (quant.)

increasing activity →

← increasing number of hydroxy groups

Reaction conditions: 4-iodobenzotrifluoride (100 µmol), methyl 3-mercaptopropanoate (200 µmol), $NiCl_2$·glyme (4 mol%), COF (3 mg), *N,N-tert*-butylisopropylamine (BIPA, 500 µmol), MeCN (anhydrous, 2 mL), 440 nm LED (2 lamp at full power). NMR yields and conversion of 4-iodobenzotrifluoride determined by ^{1}H-NMR using 1,3,5-trimethoxybenzene as internal standard. Conversion in brackets.

Table S 6. Screening experiments for the coupling of 4-bromebenzotrifluoride and pyrrolidine using different COFs.

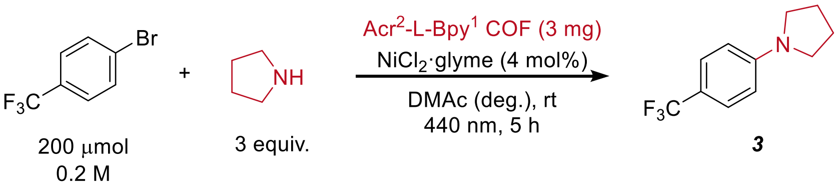

	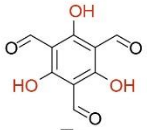 Tp	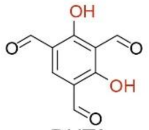 DHTA	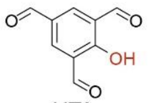 HTA	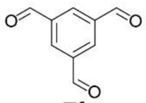 Tf
Acr:Bpy = 2:1	80 (quant.)	28 (40)	14 (29)	4 (16)

← increasing activity

← increasing number of hydroxy groups

Reaction conditions: 4-bromobenzotrifluoride (200 µmol), pyrrolidine (600 µmol), $NiCl_2$·glyme (4 mol%), COF (3 mg), DMAc (anhydrous, 1 mL), 440 nm LED (2 lamp at full power). Conversion of 4-iodobenzotrifluoride determined by ^{1}H-NMR using 1,3,5-trimethoxybenzene as internal standard; conversion noted in brackets. NMR yields determined by ^{1}H-NMR using 1,3,5-trimethoxybenzene as internal standard.

S7.5 Control studies

Table S 7. Control experiments

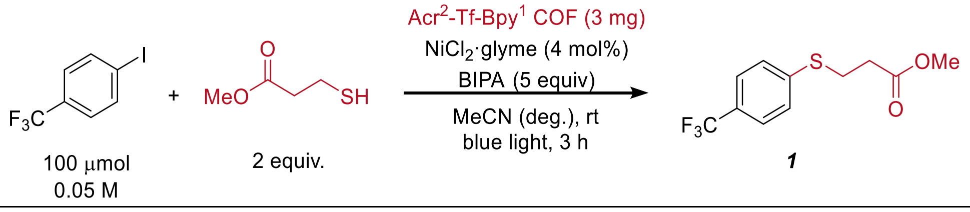

Entry	Variation	Conversion [%][b]	1 [%][c]
1	None	quant.	98
2	No COF	12	4
3	No $NiCl_2$·glyme	8	traces
4	No BIPA	10	n.d.
5	No light	8	n.d.
6	Pre-ligated (COF+$NiCl_2$·glyme)	quant.	99
7	Tp-Acr + dtbppy	35	33
8	Tf-Acr (2 mg) + Tf-Bpy (1 mg)	34	34
9	green instead of blue light, 5h	74	74
10	green instead of blue light, 5h, Tf-Bpy	24	23
11	red instead of blue light, 5h	15	9
12	red instead of blue light, 48h	quant.	99

[a]Reaction conditions: 4-iodobenzotrifluoride (100 µmol), methyl 3-mercaptopropanoate (200 µmol), $NiCl_2$·glyme (4 mol%), COF (3 mg), *N,N-tert*-butylisopropylamine (BIPA, 500 µmol), MeCN (anhydrous, 2 mL), blue light (440 nm; 2 lamp at full power), green light (525 nm; 2 lamp at full power), red light (666 nm; 2 lamp at full power). [b]Conversion of 4-iodobenzotrifluoride determined by ^{1}H-NMR using 1,3,5-trimethoxybenzene as internal standard. [c]NMR yields determined by ^{1}H-NMR using 1,3,5-trimethoxybenzene as internal standard. n.d. = not detected.

Experimental procedure for methyl 3-((4-(trifluoromethyl)phenyl)thio)propanoate

An oven dried vial (13 x 95 mm) equipped with a stir bar was charged with $NiCl_2$·glyme (2.6 mg, 12 µmol, 4 mol%), 4-iodobenzotrifluoride (0.3 mmol, 1 equiv.) and Tf-COF (9.0 mg). Subsequently, methyl 3-mercaptopropanoate (0.6 mmol, 2 equiv.), *N,N*-tert-butylisopropylamine (BIPA, 1.5 mmol, 5 equiv.), MeCN (anhydrous, 6 mL) were added and the vial was sealed with a septum and parafilm. The reaction mixture was sonicated for 5-10 min and the mixture was then degassed by bubbling N_2 for 10 min. The mixture was stirred at 800 rpm and irradiated with two LED lamps (440 nm) at full power. After the respective reaction time, one equivalent of 1,3,5-trimethoxybenzene (50.5 mg, 0.3 mmol, internal standard) was added. An aliquot (~300 µL) of the reaction mixture was diluted with DMSO-d_6 and subjected to ^{1}H-NMR analysis. The NMR sample was combined with the reaction mixture, diluted with H2O (40 mL) and extracted with ethyl acetate (3 x 30 mL). The combined organic phases were washed with aqueous NaOH (1 M, 2 x 40 ml) and brine (40 mL), dried over $MgSO_4$ and concentrated. The product was purified by flash column chromatography (SiO_2, cyclohexane/EtOAc) on a Biotage Isolera system using a 25 g cartridge and an elution gradient of 0-5 % of ethyl acetate in cyclohexane. The product was gained in 91% yield (72 mg, 273 µmol) as a white solid. The final product was characterized by ^{1}H-NMR, ^{13}C-NMR, ^{19}F-NMR.

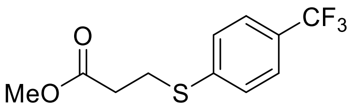

Methyl 3-((4-(trifluoromethyl)phenyl)thio)propanoate: ^{1}H NMR (700 MHz, $CDCl_3$) δ 7.52 (d, J = 8.3 Hz, 2H), 7.39 (d, J = 8.2 Hz, 2H), 3.69 (s, 3H), 3.24 (t, J = 7.4 Hz, 2H), 2.67 (t, J = 7.4 Hz, 2H) ppm. ^{13}C NMR (176 MHz, $CDCl_3$) δ 172.0, 141.1, 128.3, 128.1 (q, J = 32.6 Hz), 125.93 (q, J = 3.7 Hz), 124.20 (q, J = 271.8 Hz), 52.1, 33.9, 27.9 ppm. ^{19}F NMR (659 MHz, $CDCl_3$) δ -62.51 (s, 3F) ppm. ESI-MS [M+H^+] (m/z)): 265.0503 (th.: 265.0505).

These data are in full agreement with those previously published in the literature.[23]

S8. Photocatalysis - recycling studies

S8.1 Reaction procedure

An oven dried vial (13 x 80 mm) equipped with a stir bar was charged with Acr[2]-Tf-Bpy[1] (9 mg) and $NiCl_2$·glyme (2.6 mg, 12 µmol, 4 mol%). Subsequently, 4-iodobenzotrifluoride (0.3 mmol, 1 equiv.) methyl 3-mercaptopropanoate (0.6 mmol, 2 equiv.), *N,N*-tert-butylisopropylamine (BIPA, 1.5 mmol, 5 equiv.), MeCN (anhydrous, 6 mL) were added and the vial was sealed with a septum and Parafilm. The reaction mixture was sonicated for 5-10 min followed by stirring for 5 min until fine dispersion of the solids was achieved and the mixture was then degassed by bubbling N_2 for 10 min. The mixture was stirred at 800 rpm and irradiated with one or two LED lamps (440 nm) at full power. After the respective reaction time, one equivalent of 1,3,5-trimethoxybenzene (50.5 mg, 0.3 mmol) was added and the mixture was stirred for 5 min. The reaction mixture was centrifuged at 3500 rpm for 10 min and the liquid phase was carefully separated and analyzed by ^{1}H-NMR. The Acr[2]-Tf-Bpy[1] COF was washed 2 times with MeCN (anhydrous, 3 mL, followed by centrifugation at 3500 rpm for 10 min and separation of the liquid phase) and reused in the next reaction.

Table S 8. Catalyst Recycling experiments of the C-S arylation with the 440 nm setup using an *in situ* generated catalyst.[a]

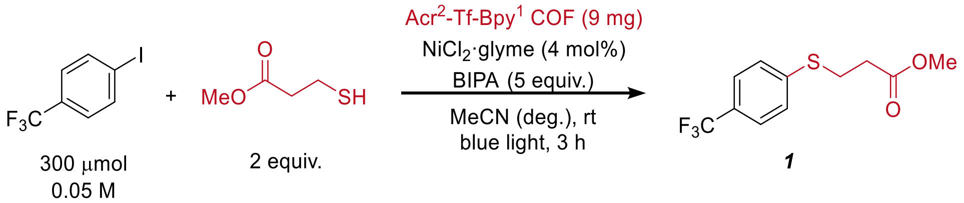

1

Entry	Cycle	Conversion [%][b]	1 [%][c]
1	1	quant.	96
2	2	quant.	98
3	3	quant.	97
4	4	quant.	98
5	5	quant.	97

[a]Reaction conditions: 4-iodobenzotrifluoride (300 µmol), methyl 3-mercaptopropanoate (600 µmol), $NiBr_2$·glyme (4 mol%), COF (9 mg), *N,N-tert*-butylisopropylamine (BIPA, 1.5 mmol), MeCN (anhydrous, 6 mL), blue light (440 nm; 2 lamp at full power). [b]Conversion of 4-iodobenzotrifluoride determined by ^{1}H-NMR using 1,3,5-trimethoxybenzene as internal standard. [c]NMR yields determined by ^{1}H-NMR using 1,3,5-trimethoxybenzene as internal standard. n.d. = not detected.

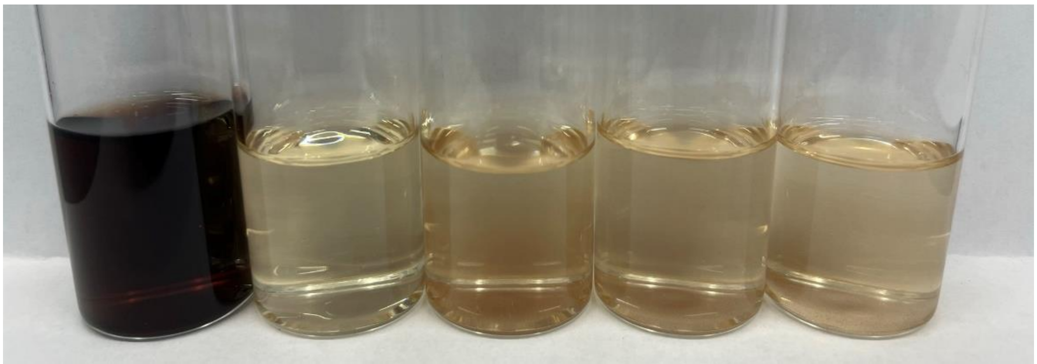

Figure S 30. Reaction mixtures (see Table S 8) by centrifugation.

S8.2 Analysis of recycled Tp-Acr COF

Table S 9. ICP-OES measurements of the nickel content of the Acr^2-Tf-Bpy^1 COF, Acr^2-Tf-[Ni]^1 COF in- and ex-situ preparation and the recovered Acr^2-Tf-Bpy^1 COF after 5 cycles.

Sample	Ni [mg/g catalyst]	Occupancy of Bpy sites [%]
Acr^2-Tf-Bpy^1 COF	0.317	-
Acr^2-Tf-Bpy^1-Ni COF (in situ)	13.9	20.0
Acr^2-Tf- Bpy^1-Ni COF (ex situ)	3.59	5.1
Acr^2-Tf-Bpy^1-Ni COF (after 5 cycles) [a]	10.3	14.7

[a]Acr^2-Tf-Bpy^1 COF after 5 reaction cycles

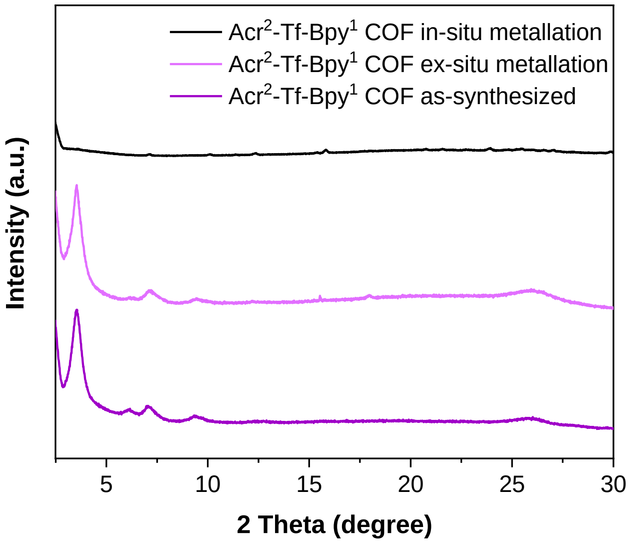

Figure S 31. PXRD analyses of Acr^2-Tf-Bpy^1 before and after photocatalysis (in-situ metalation), and ex-situ metalation, showing that the framework structure loses crystallinity under photocatalysis conditions, compared to metalation without light irradiation

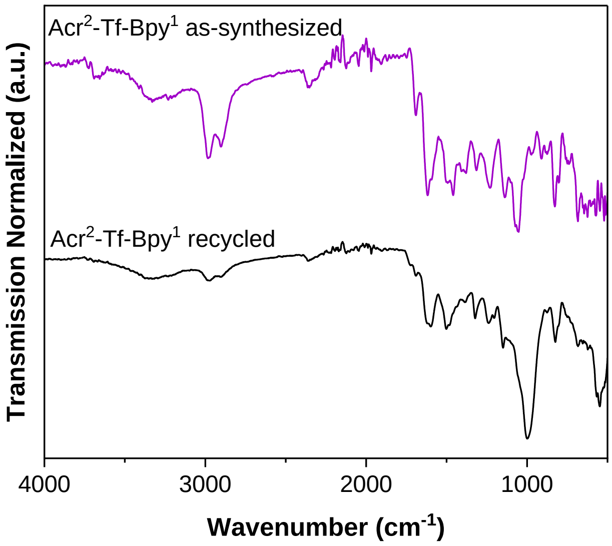

Figure S 32. FT-IR analyses of Acr^2-Tf-Bpy^1 COF before and after photocatalysis, showing that the framework structure remains stable under photocatalysis conditions.

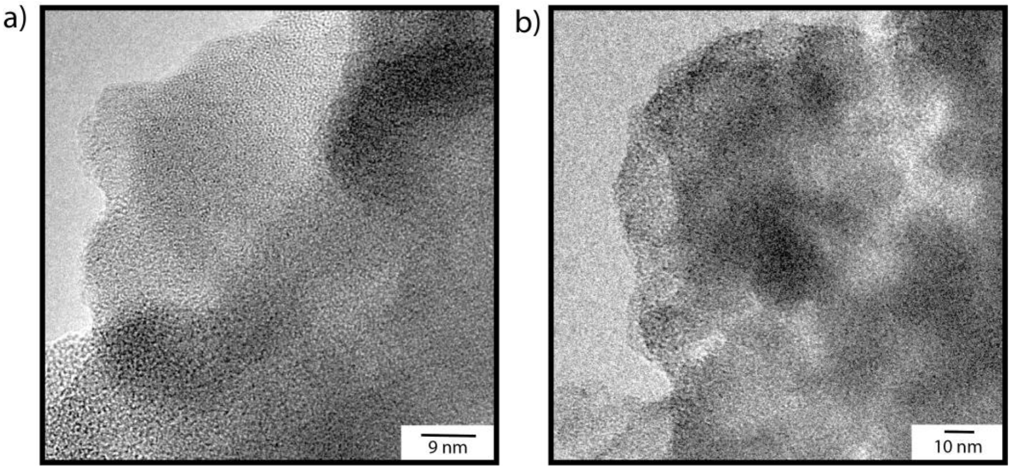

Figure S 33. TEM analyses of Acr^2-Tf-Bpy^1 before (a) and after photocatalysis (b), respectively, showing the morphology and the layered structure of the COF matrix, unchanged by photocatalysis.

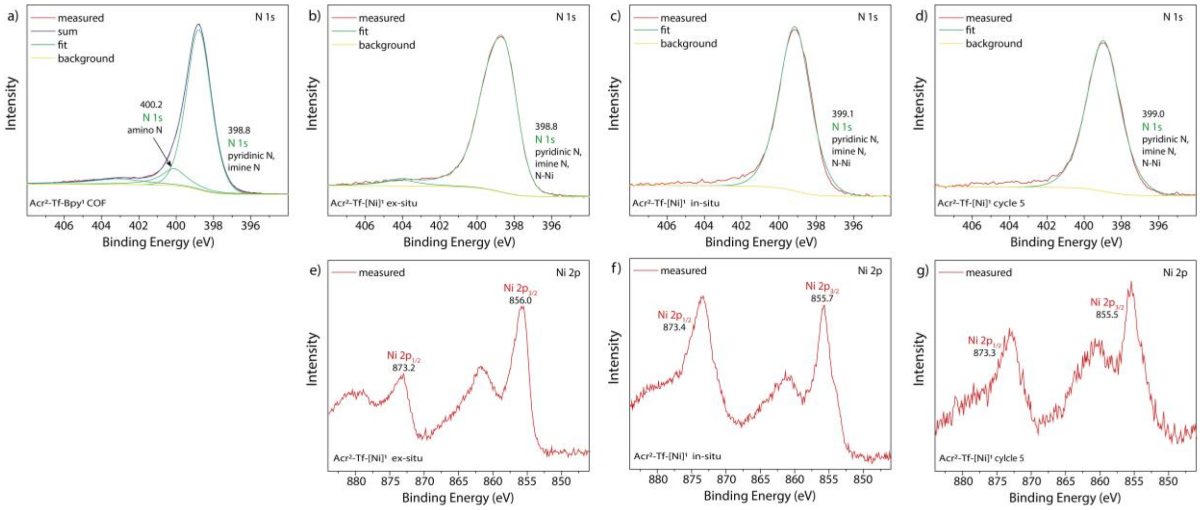

Figure S 34. (a, b, c, d) N 1s XPS core level spectra of the Acr^2-Tf-Bpy^1 COF (a), ex-situ metallated Acr^2-Tf- Bpy^1-Ni COF (b), in-situ metallated Acr^2-Tf-Bpy^1-Ni COF (c) and Acr^2-Tf-Bpy^1-Ni COF after 5 recycling cycles (d) of photocatalytic dual nickel C-S cross coupling. (e, f, g) Ni 2p XPS core level spectra of the ex-situ metallated Acr^2-Tf-Bpy^1-Ni COF (e), in-situ metallated Acr^2-Tf-Bpy^1-Ni COF (f) and Acr^2-Tf-Bpy^1-Ni COF after 5 recycling cycles (g) of photocatalytic dual nickel C-S cross coupling.

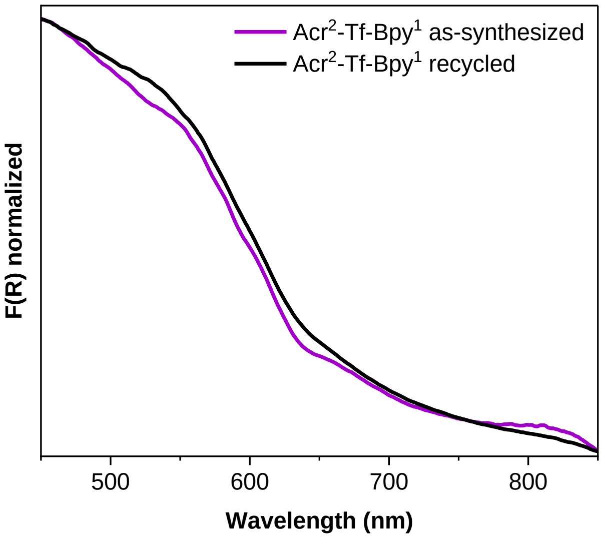

Figure S 35. UV-vis diffuse reflectance spectroscopy analysis of Acr^2-Tf-Bpy^1 COF before and after in-situ metalation during photocatalysis, showing no effect of the nickel on the optical properties of the framework.

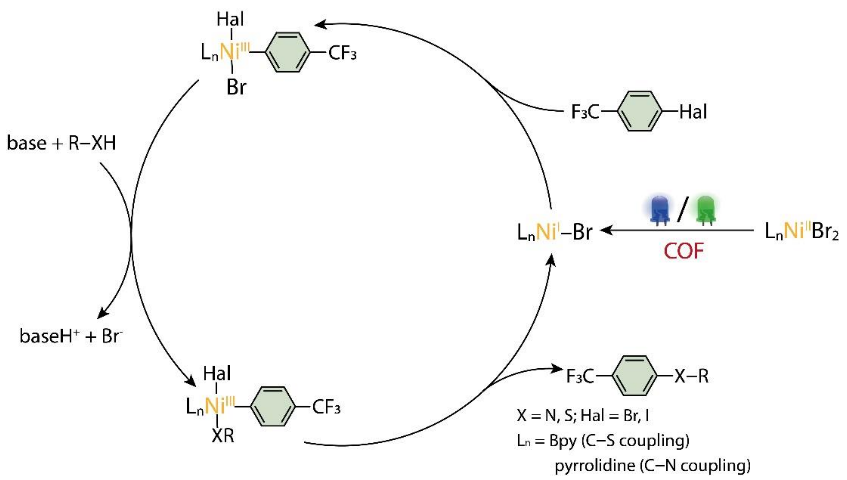

Figure S 36. Proposed mechanisms invoked in the nickel mediated metallaphotocatalytic C–X cross-coupling by a thermally sustained $Ni^{I/III}$ cycle.

S10. References

[1] "https://www.kessil.com/photoreaction/PR160L.php," **n.d.**

[2] D. H. O'Donovan, B. Kelly, E. Diez-Cecilia, M. Kitson, I. Rozas, *New J. Chem.* **2013**, *37*, 2408–2418.

[3] J. H. Chong, M. Sauer, B. O. Patrick, M. J. MacLachlan, *Org. Lett.* **2003**, *5*, 3823–3826.

[4] S. Haldar, D. Chakraborty, B. Roy, G. Banappanavar, K. Rinku, D. Mullangi, P. Hazra, D. Kabra, R. Vaidhyanathan, *J. Am. Chem. Soc.* **2018**, *140*, 13367–13374.

[5] A. A. Anderson, T. Goetzen, S. A. Shackelford, S. Tsank, *Synth. Commun.* **2000**, *30*, 3227–3232.

[6] M. Traxler, S. Gisbertz, P. Pachfule, J. Schmidt, J. Roeser, S. Reischauer, J. Rabeah, B. Pieber, A. Thomas, *Angew. Chem. Int. Ed.* **2022**, *61*, e202117738.

[7] G. Kresse, J. Furthmüller, *Comput. Mater. Sci.* **1996**, *6*, 15–50.

[8] G. Kresse, J. Furthmüller, *Phys. Rev. B - Condens. Matter Mater. Phys.* **1996**, *54*, 11169–11186.

[9] J. P. Perdew, K. Burke, M. Ernzerhof, *Phys. Rev. Lett.* **1996**, *77*, 3865–3868.

[10] S. Grimme, J. Antony, S. Ehrlich, H. Krieg, *J. Chem. Phys.* **2010**, *132*, 154104.

[11] S. Grimme, S. Ehrlich, L. Goerigk, *J. Comput. Chem.* **2011**, *32*, 1456–1465.

[12] P. E. Blöchl, *Phys. Rev. B* **1994**, *50*, 17953–17979.

[13] G. Kresse, D. Joubert, *Phys. Rev. B - Condens. Matter Mater. Phys.* **1999**, *59*, 1758–1775.

[14] J. Heyd, G. E. Scuseria, M. Ernzerhof, *J. Chem. Phys.* **2003**, *118*, 8207–8215.

[15] J. Heyd, G. E. Scuseria, M. Ernzerhof, *J. Chem. Phys.* **2006**, *124*, 219906.

[16] F. Neese, *WIREs Comput. Mol. Sci.* **2012**, *2*, 73–78.

[17] F. Weigend, R. Ahlrichs, *Phys. Chem. Chem. Phys.* **2005**, *7*, 3297–3305.

[18] T. Yanai, D. P. Tew, N. C. Handy, *Chem. Phys. Lett.* **2004**, *393*, 51–57.

[19] P. J. Stephens, F. J. Devlin, C. F. Chabalowski, M. J. Frisch, *J. Phys. Chem.* **1994**, *98*, 11623–11627.

[20] A. D. Becke, *J. Chem. Phys.* **1993**, *98*, 5648–5652.

[21] C. Lee, W. Yang, R. G. Parr, *Phys. Rev. B* **1988**, *37*, 785–789.

[22] E. Runge, E. K. U. Gross, *Phys. Rev. Lett.* **1984**, *52*, 997–1000.

[23] J. I. Higham, J. A. Bull, *Chem. Commun.* **2020**, *56*, 4587–4590.

S11. Author Contributions

M.T. and S.R. contributed equally to this work and either has the right to list them self first in bibliographic documents. All authors contributed extensively to the work presented in this paper. M.T., S.R., B.P. and A.T. conceived the research project. M.T. conducted the synthesis of linkers as well as COFs and performed characterizations. S.R. evaluated the COFs in cross-couplings. S.V. performed and evaluated the XPS analyses. J. Roeser was responsible for simulations of the COFs. J. Rabeah carried out EPR analyses. C.P. and P.S. conducted theoretical calculations. M.T. and S.R. wrote the manuscript with the input from all authors.

S12. Copies of NMR spectra

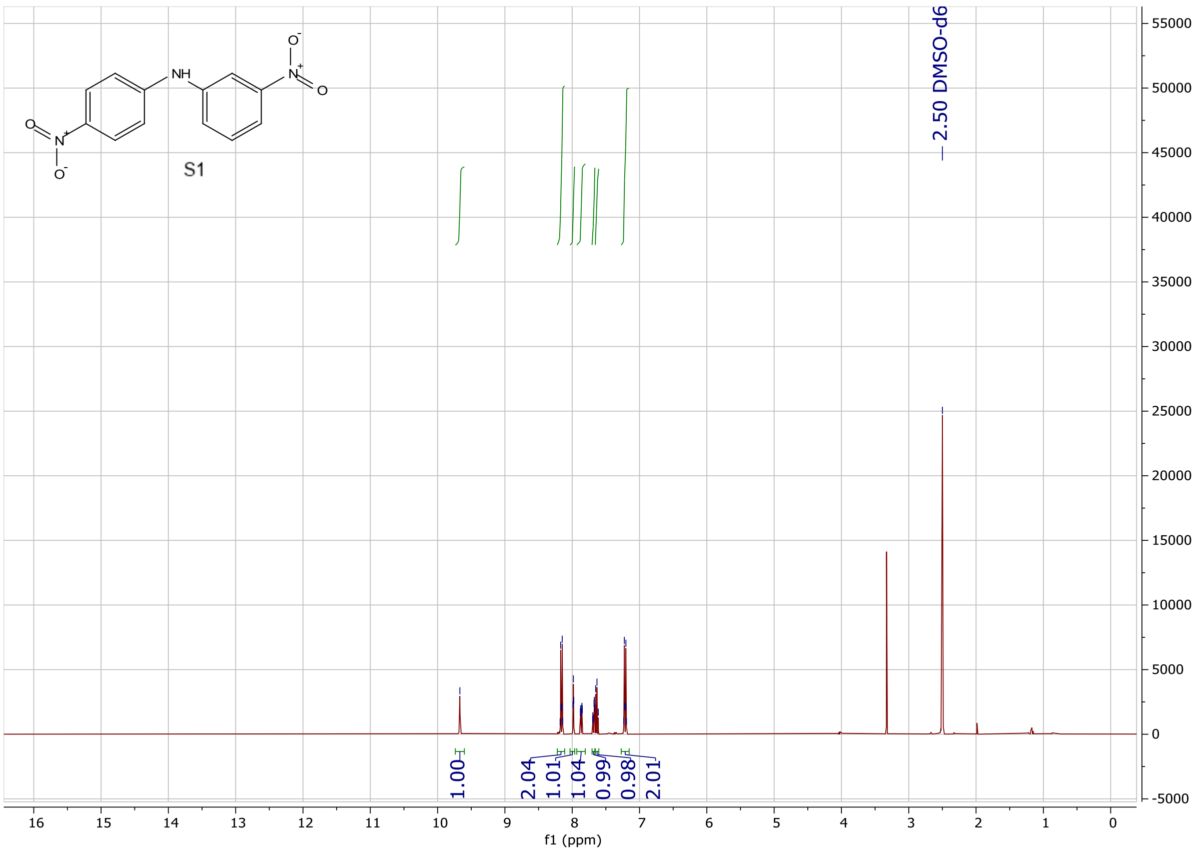

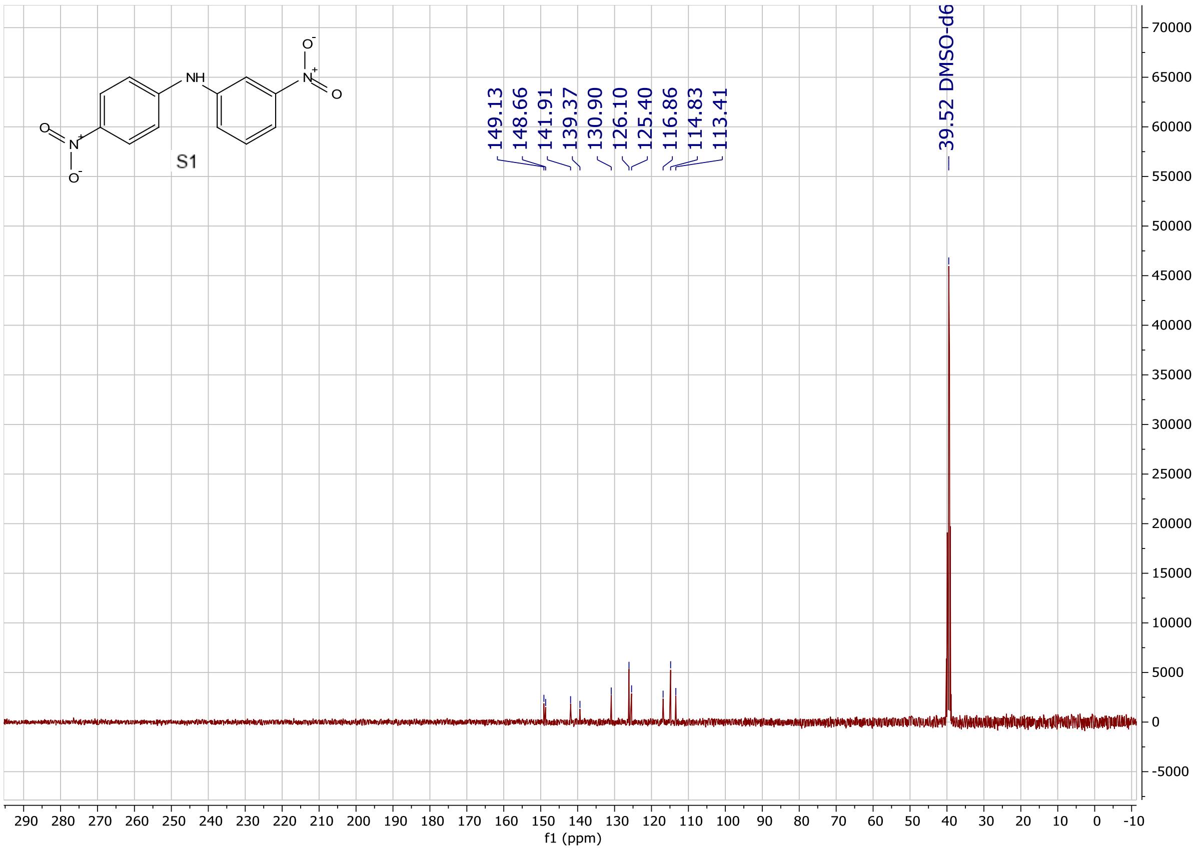

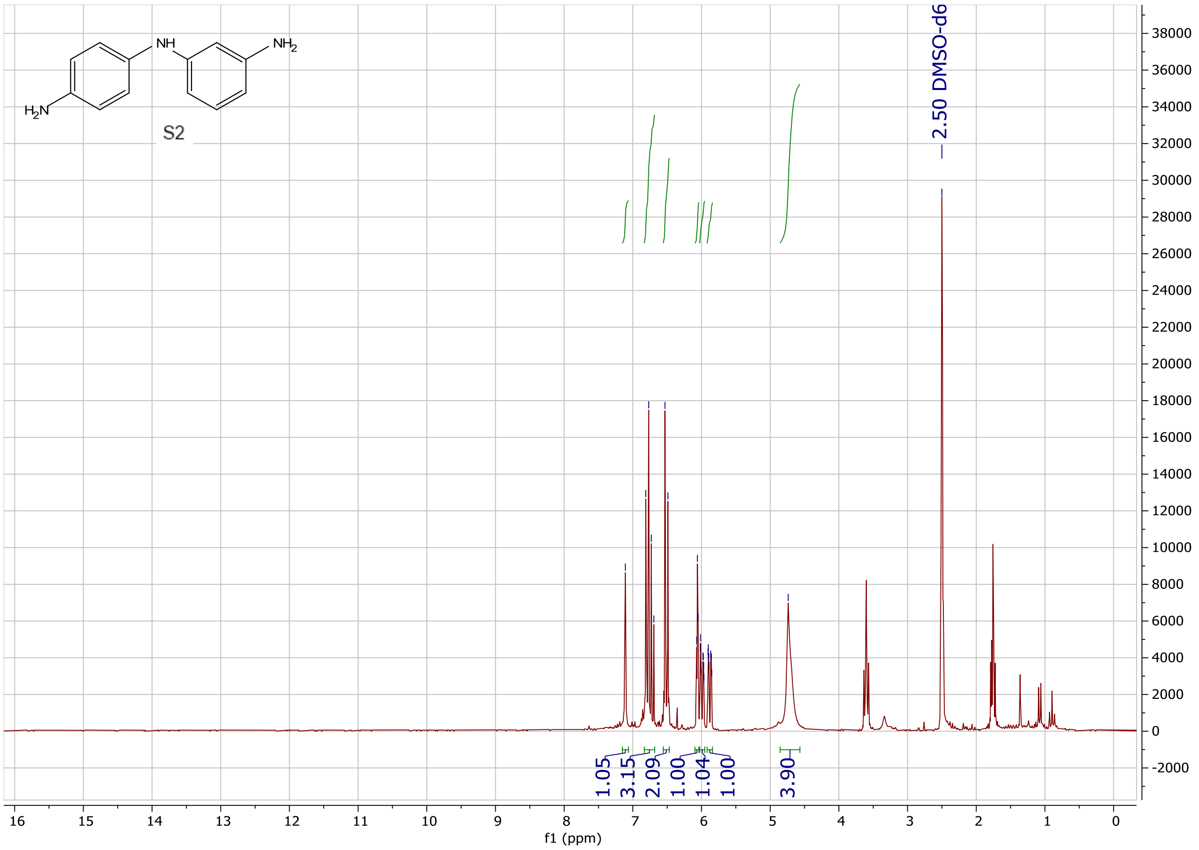
NH
NH2
H2N
S2
2.50 DMSO-d6
1.05
3.15
2.09
1.00
1.04
1.00
3.90
f1 (ppm)

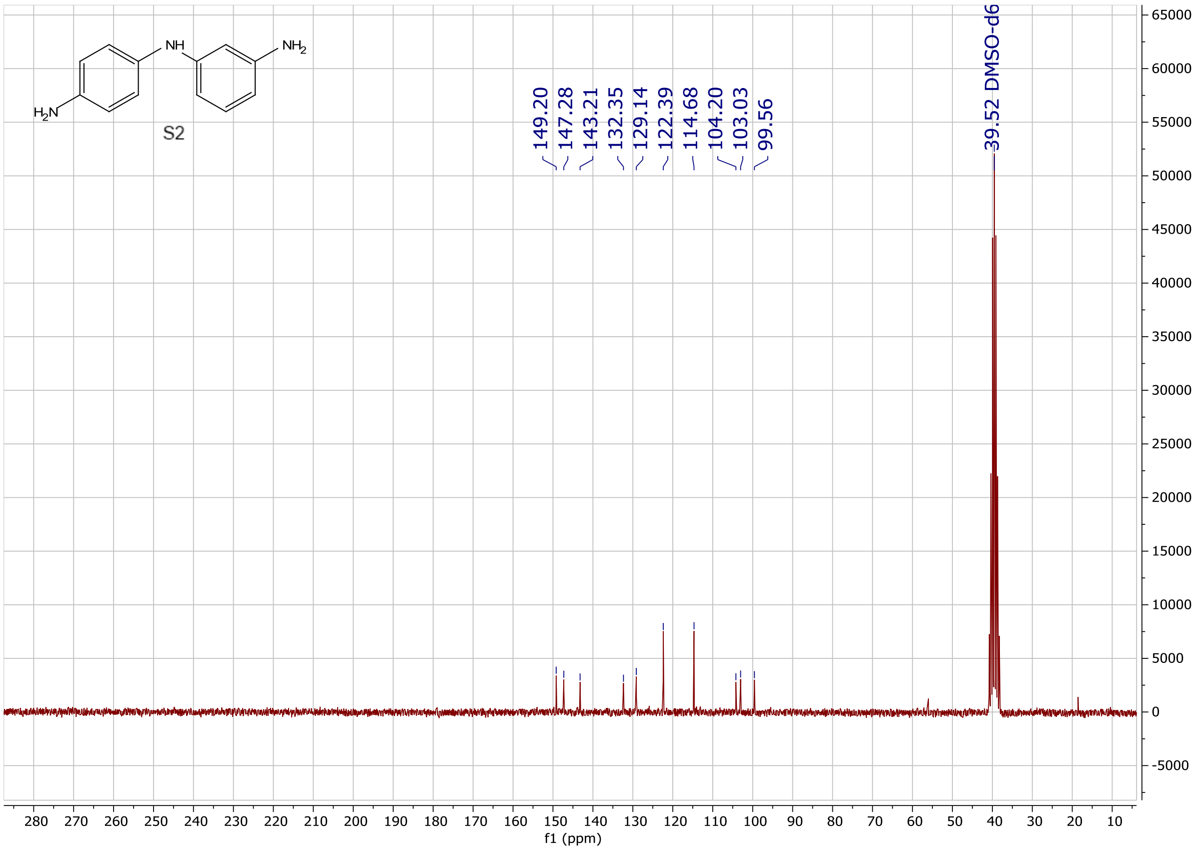
NH
NH2
H2N
S2
149.20
147.28
143.21
132.35
129.14
122.39
114.68
104.20
103.03
99.56
39.52 DMSO-d6
f1 (ppm)

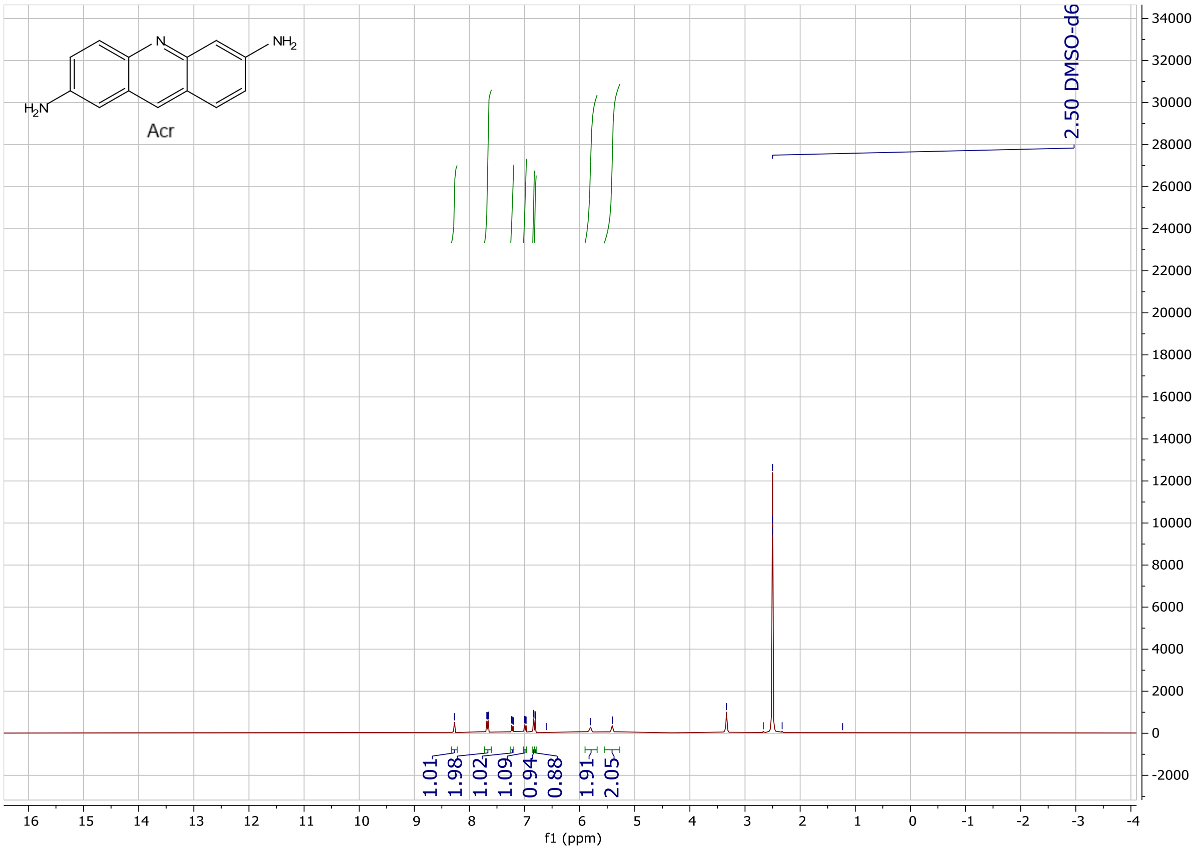

NH2
H2N
N
Acr
2.50 DMSO-d6
1.01
1.98
1.02
1.09
0.94
0.88
1.91
2.05
f1 (ppm)

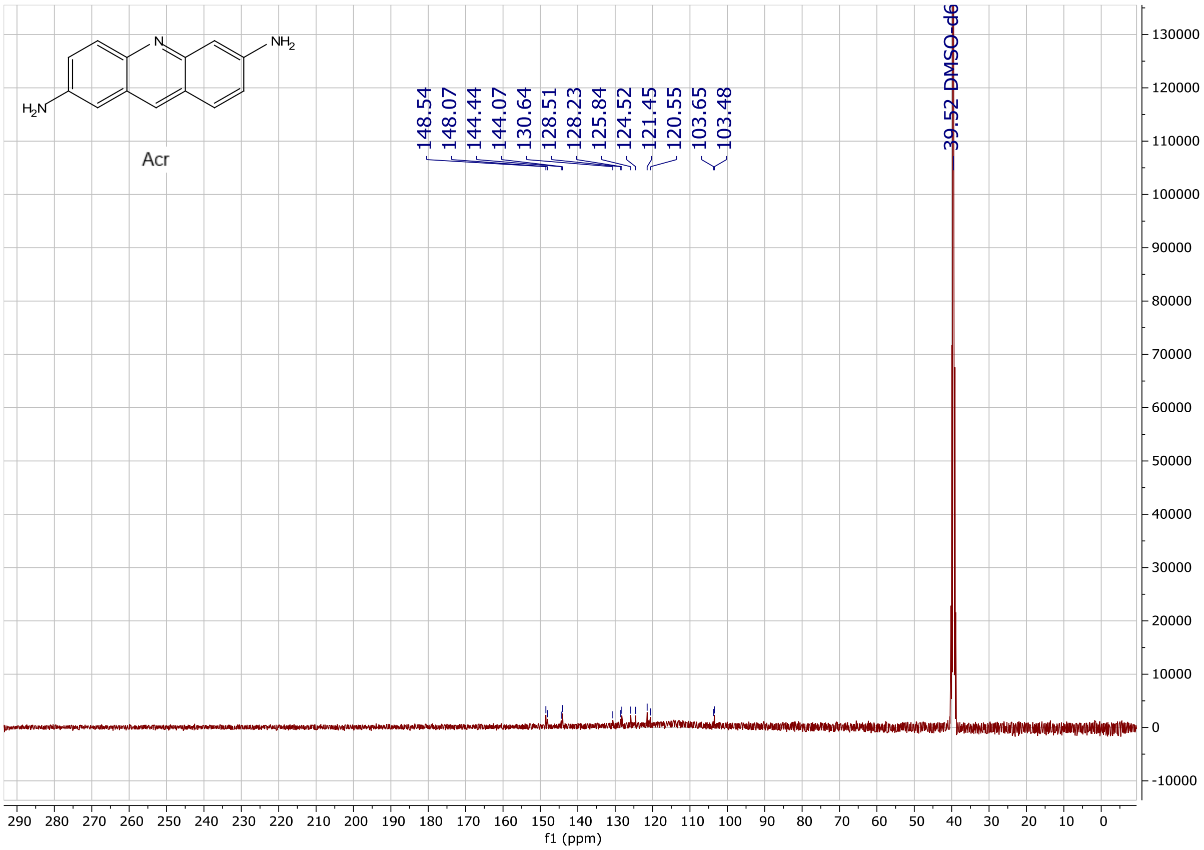

NH2
H2N
N
Acr
148.54
148.07
144.44
144.07
130.64
128.51
128.23
125.84
124.52
121.45
120.55
103.65
103.48
39.52 DMSO-d6
f1 (ppm)

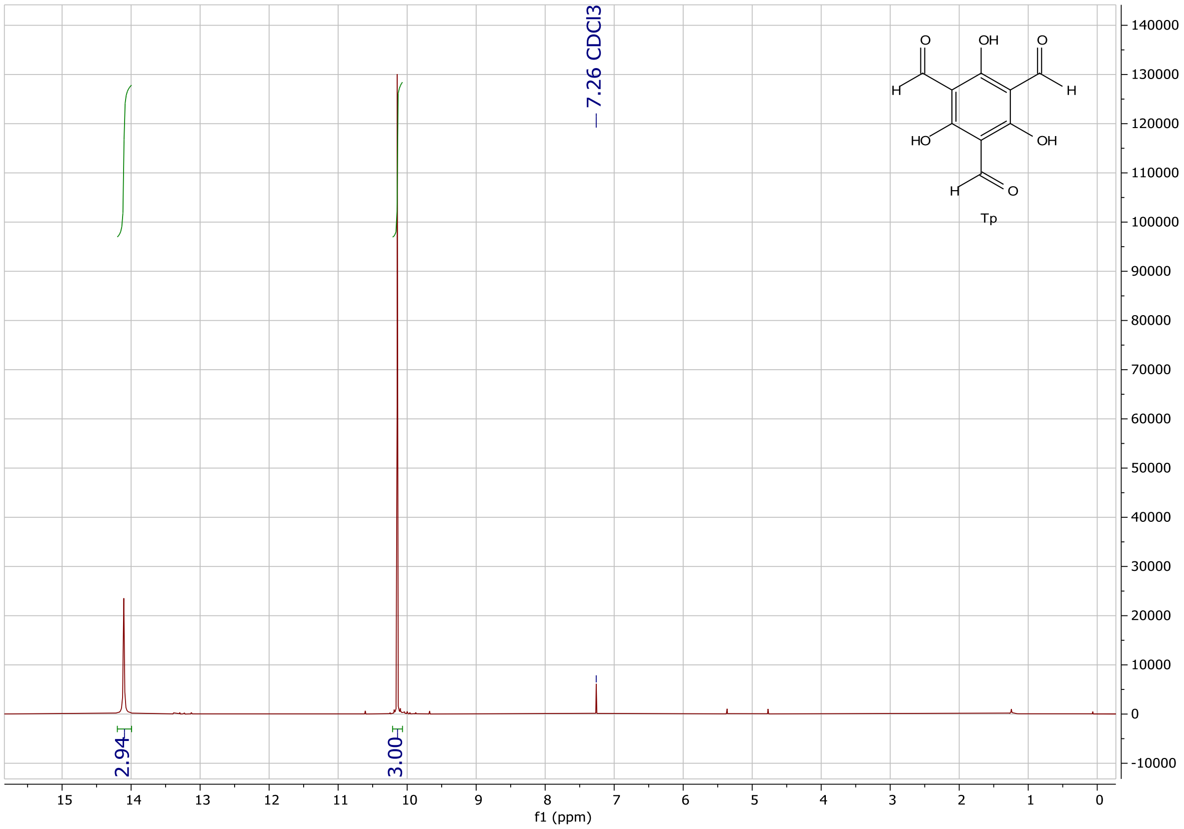
7.26 CDCl3
2.94
3.00
f1 (ppm)
Tp

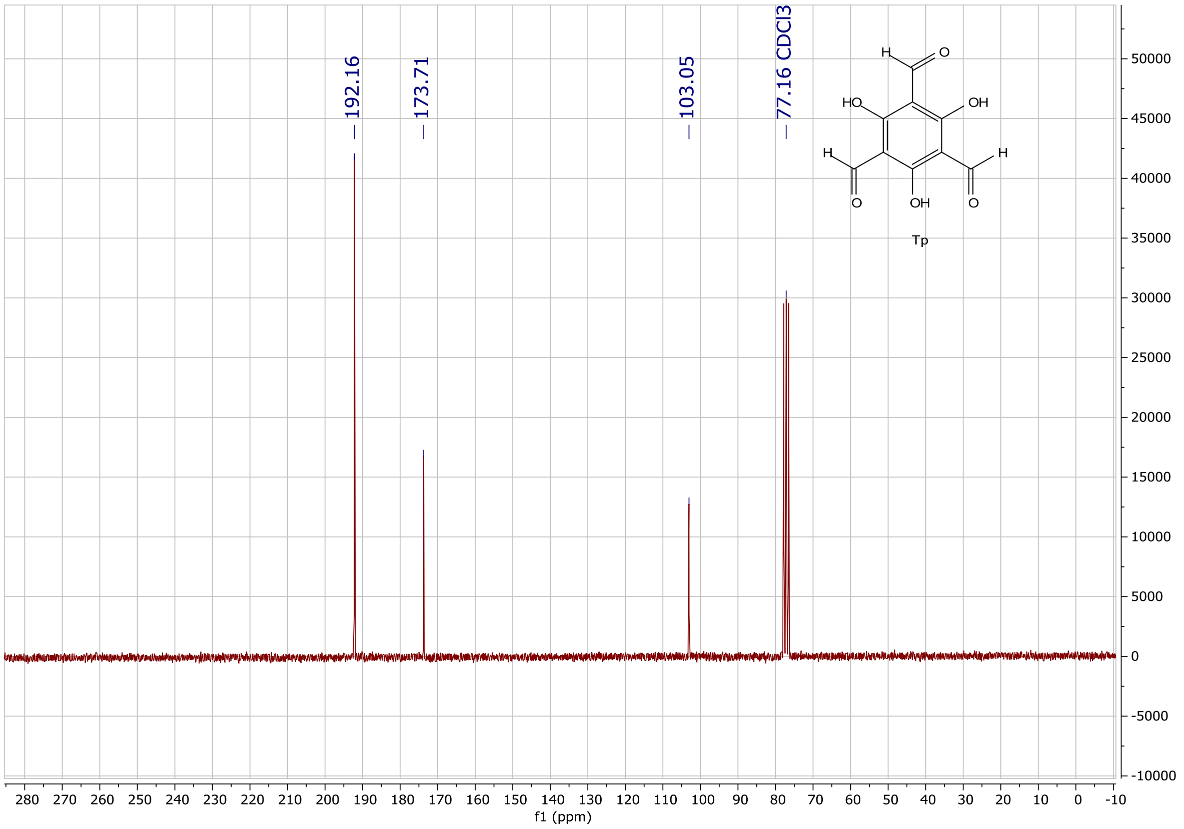
192.16
173.71
103.05
77.16 CDCl3
f1 (ppm)
Tp

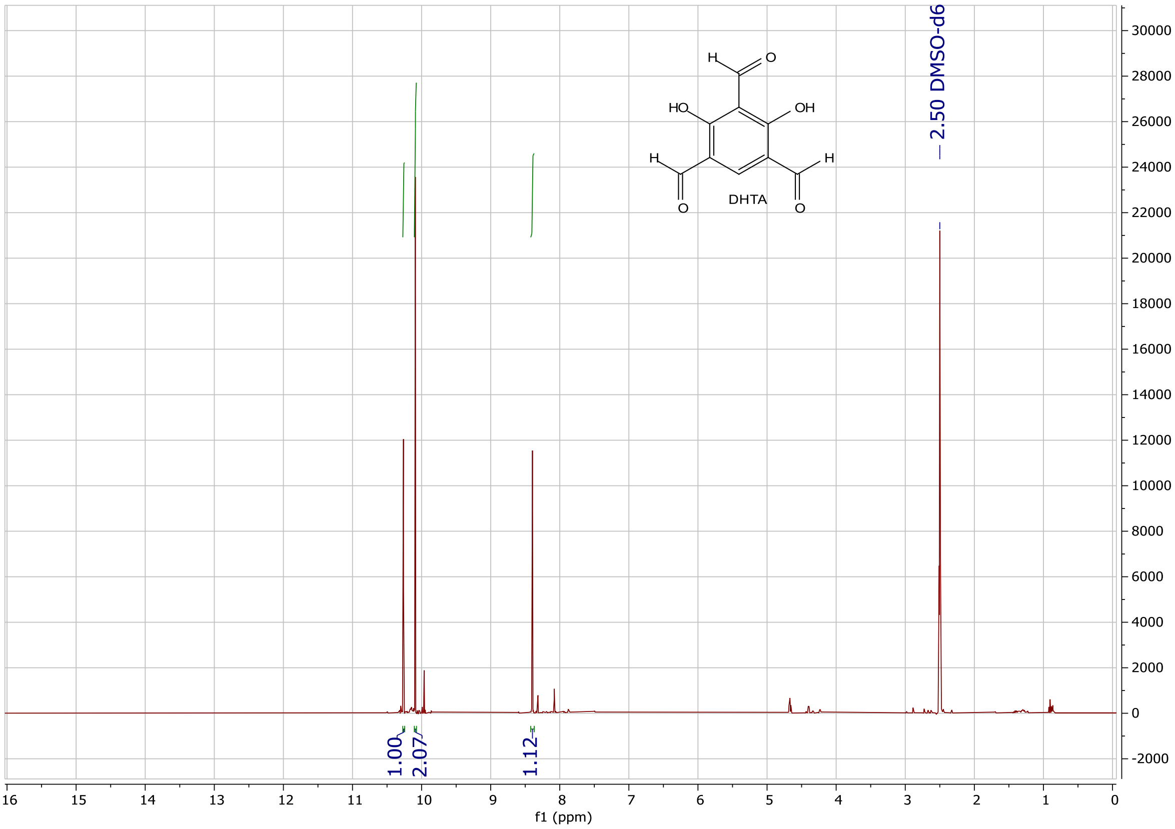
DHTA
HO
OH
2.50 DMSO-d6
1.00
2.07
1.12
f1 (ppm)

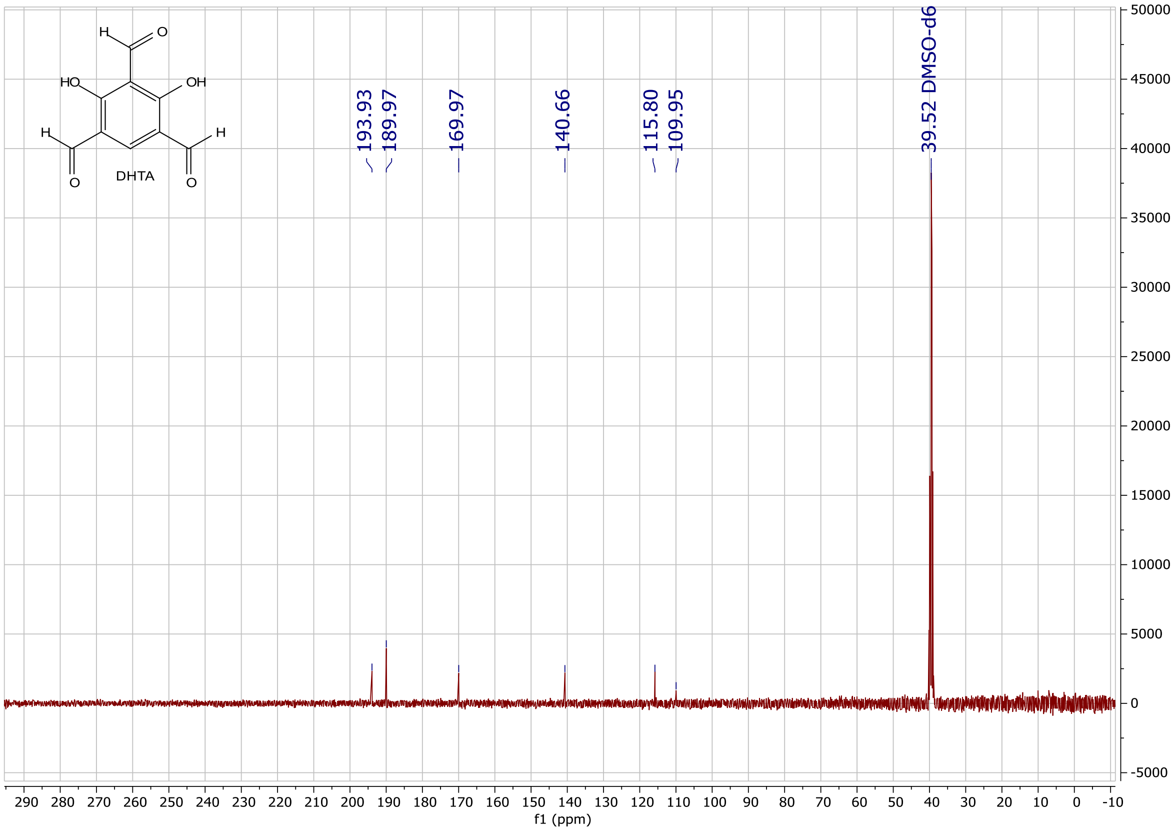
DHTA
HO
OH
193.93
189.97
169.97
140.66
115.80
109.95
39.52 DMSO-d6
f1 (ppm)

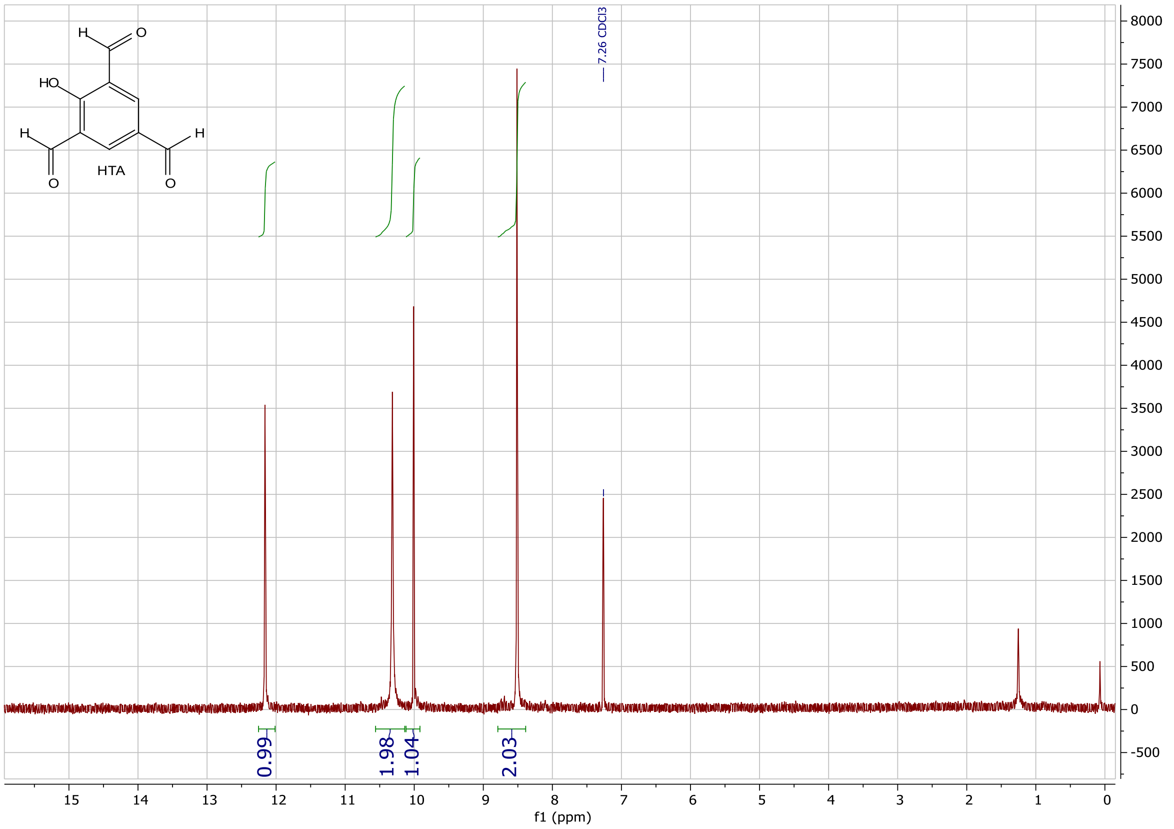
HO
H
O
HTA
7.26 CDCl3
0.99
1.98
1.04
2.03
f1 (ppm)

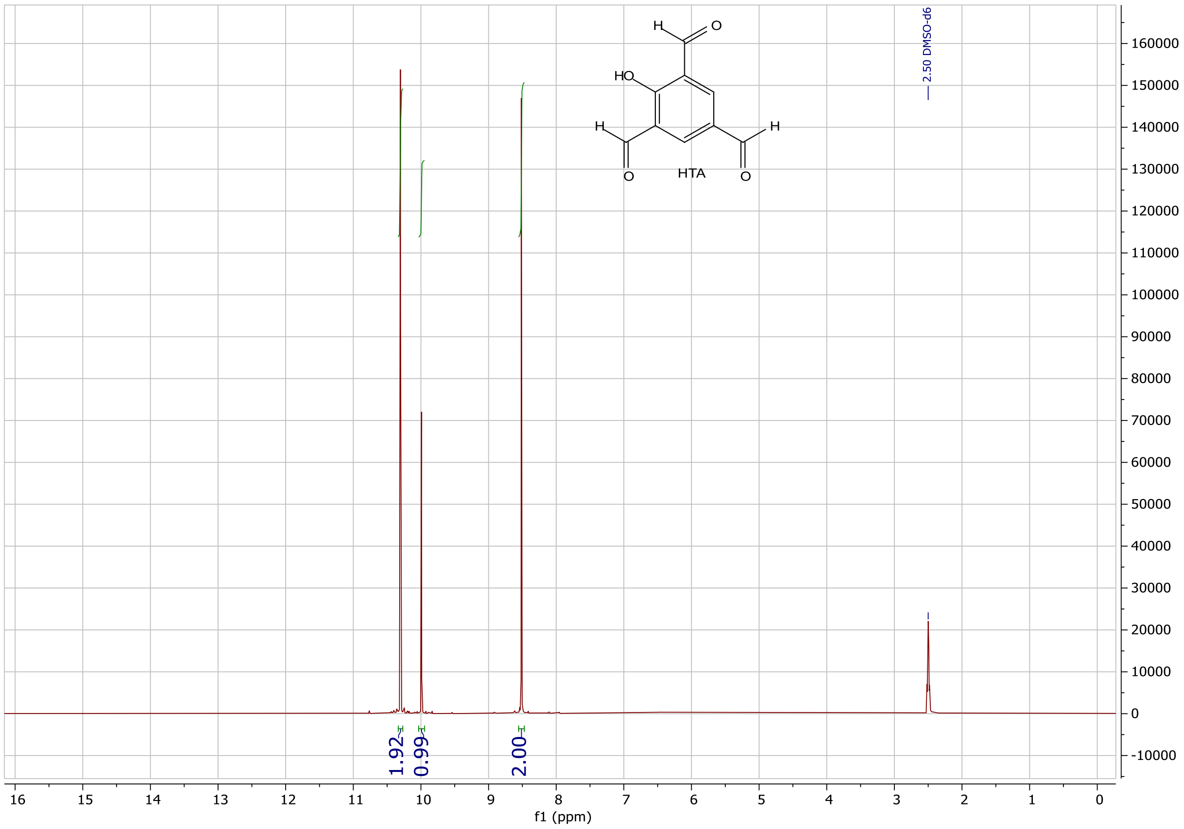
HO
H
O
HTA
2.50 DMSO-d6
1.92
0.99
2.00
f1 (ppm)

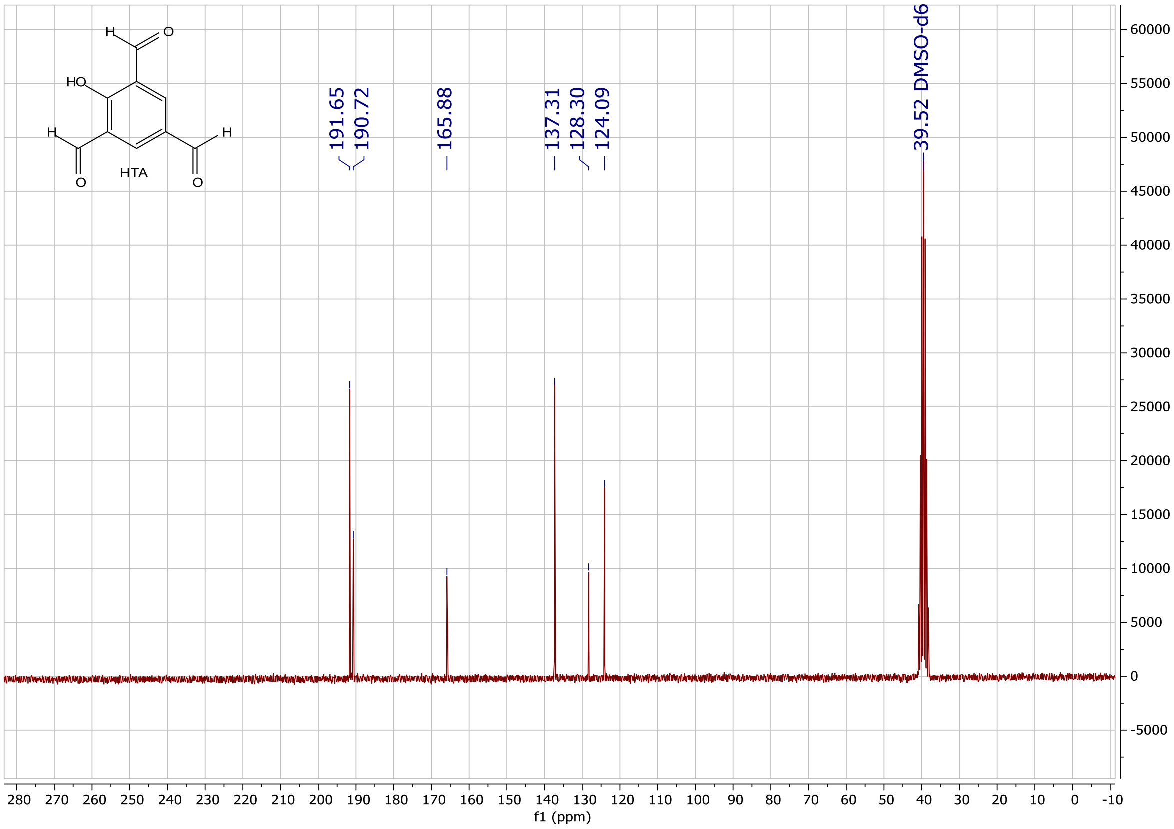
HTA
HO
191.65
190.72
165.88
137.31
128.30
124.09
39.52 DMSO-d6
f1 (ppm)

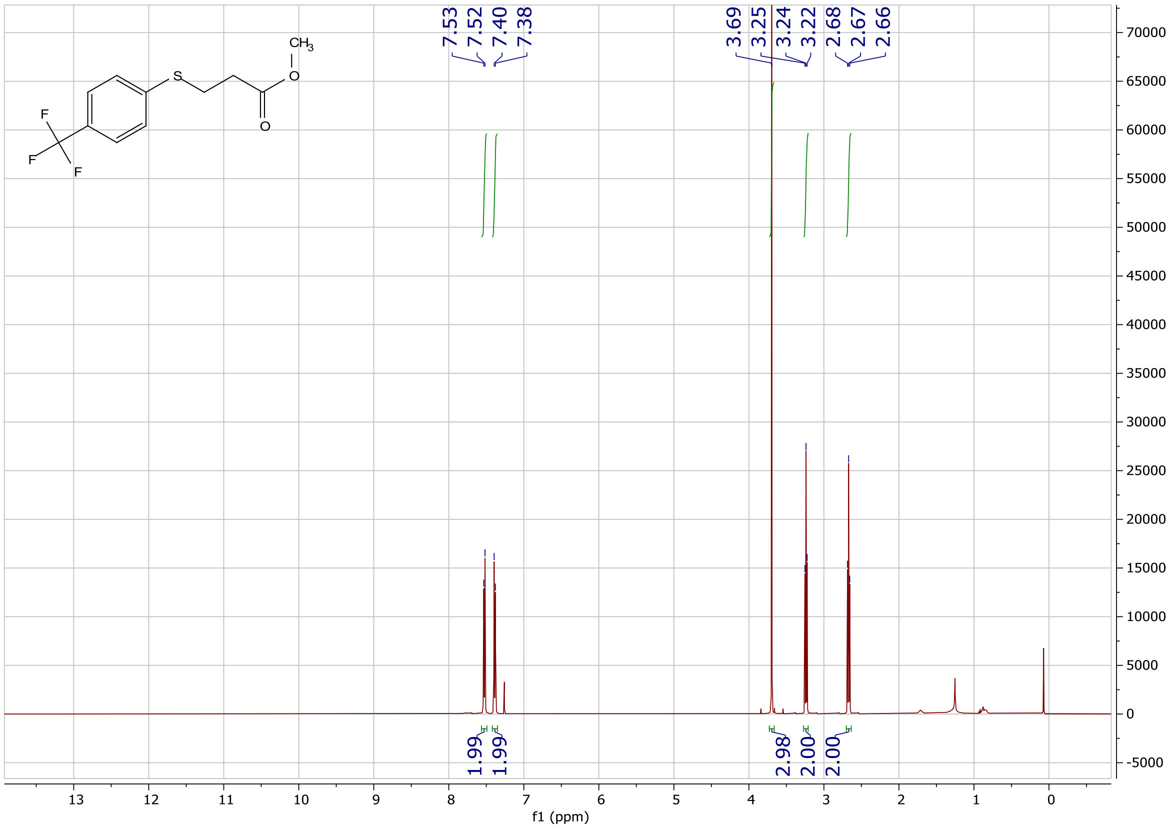
7.53
7.52
7.40
7.38
3.69
3.25
3.24
3.22
2.68
2.67
2.66
1.99
1.99
2.98
2.00
2.00
f1 (ppm)

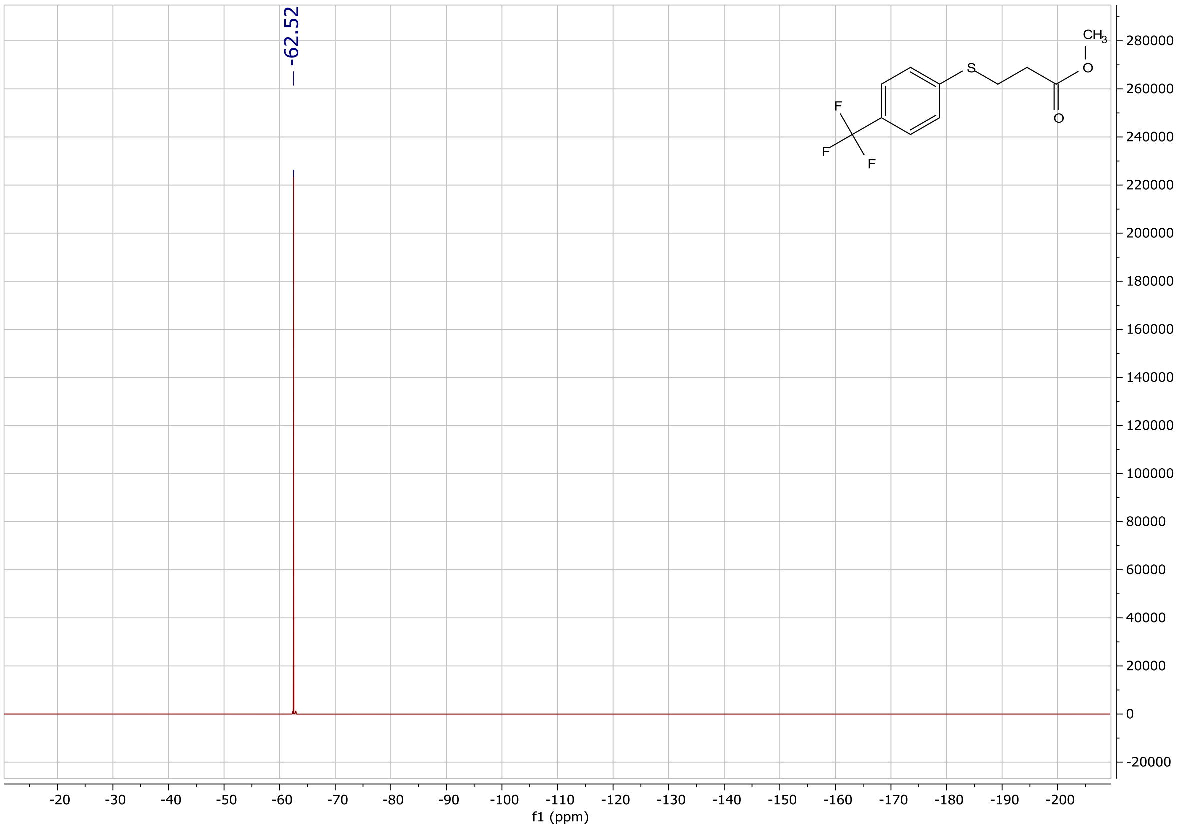
-62.52
f1 (ppm)

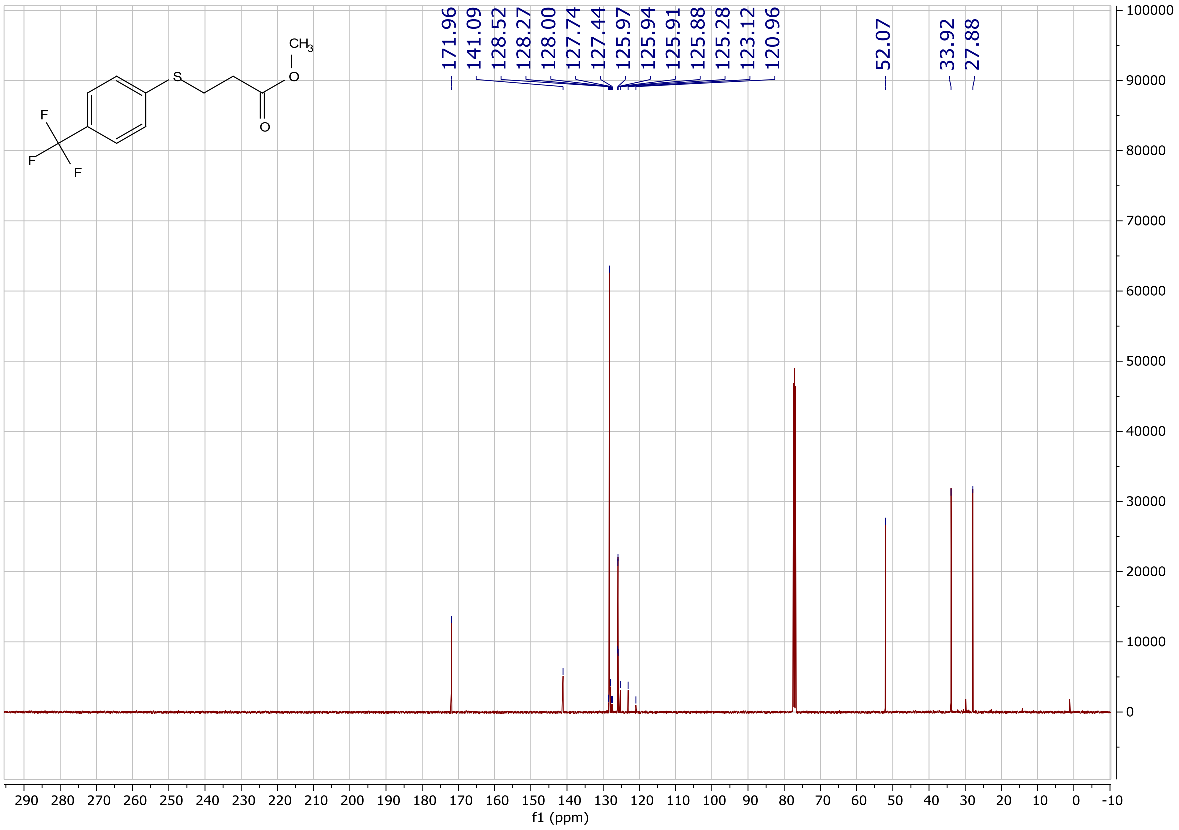
171.96
141.09
128.52
128.27
128.00
127.74
127.44
125.97
125.94
125.91
125.88
125.28
123.12
120.96
52.07
33.92
27.88
f1 (ppm)
